# Supplementary material for: BRain health and healthy AgeINg in retired rugby union players, the BRAIN Study: study protocol for an observational study in the UK
Source: BMJ Open. 2017 Dec 26;7(12):e017990. doi: 10.1136/bmjopen-2017-017990 (PMC5770902; doi:10.1136/bmjopen-2017-017990)
Supplement: Author's manuscript [file bmjopen-2017-017990.draft_revisions.pdf]

BMJ Open is committed to open peer review. As part of this commitment we make the peer review history of every article we publish publicly available.

When an article is published we post the peer reviewers' comments and the authors' responses online. We also post the versions of the paper that were used during peer review. These are the versions that the peer review comments apply to.

The versions of the paper that follow are the versions that were submitted during the peer review process. They are not the versions of record or the final published versions. They should not be cited or distributed as the published version of this manuscript.

BMJ Open is an open access journal and the full, final, typeset and author-corrected version of record of the manuscript is available on our site with no access controls, subscription charges or pay-per-view fees (http://bmjopen.bmj.com).

If you have any questions on BMJ Open's open peer review process please email editorial.bmjopen@bmj.com

BMJ Open

BRain health and healthy AgeINg in retired Rugby union players, the BRAIN study – study protocol

Journal:	*BMJ Open*
Manuscript ID	bmjopen-2017-017990
Article Type:	Protocol
Date Submitted by the Author:	05-Jun-2017

Complete List of Authors:	Gallo, Valentina; Queen Mary, University of London, Centre for Primary Care and Public Health<br>McElvenny, Damien; Institute of Occupational Medicine,<br>Hobbs, Catherine; London School of Hygiene and Tropical Medicine Faculty of Epidemiology and Population Health<br>Davoren, Donna; London School of Hygiene and Tropical Medicine Faculty of Epidemiology and Population Health<br>Morris, Huw ; UCL<br>Crutch, Sebastian; University College London Institute of Neurology<br>Zetterberg, Henrik; Institute of Neuroscience and Physiology, Sahlgrenska Academy, University of Gothenburg., Department of Psychiatry and Neurochemistry<br>Fox, Nick; UCL, Dementia Research Centre<br>Kemp, Simon<br>Cross, Matthew; Rugby Football Union<br>Arden, Nigel; Nuffield Department of Orthopaedics, ;<br>Davies, Madeleine; University of Oxford, Nuffield Department of Orthopaedics, Rheumatology and Musculoskeletal Science<br>Malaspina, Andrea; Queen Mary University of London<br>Pearce, Neil; London School of Hygiene and Tropical Medicine

**Primary Subject Heading**:	Sports and exercise medicine
Secondary Subject Heading:	Epidemiology, Neurology
Keywords:	Rugby, concussion, Rugby Union, cross-sectional study, cognitive function, healthy ageing

SCHOLARONE™
Manuscripts

BRain health and healthy AgeINg in retired Rugby union players, the BRAIN study – study protocol

Valentina Gallo[1,2,3], Damien McElvenny[4], Catherine Hobbs[3], Donna Davoren[3], Huw Morris[5], Sebastian Crutch[6], Henrik Zetterberg[7,8,9,10], Nick C Fox[6], Simon Kemp[11], Matt Cross[11], Nigel Arden[12], Madeline Davies[12], Andrea Malaspina[13], Neil Pearce[3]

1. Centre for Primary Care and Public Health, Queen Mary University of London, London, UK
2. School of Public Health, Imperial College London, London, UK
3. London School of Hygiene and Tropical Medicine, London, UK
4. Institute of Occupational Medicine, Edinburgh, UK
5. University College London, London, UK
6. Dementia Research Centre, UCL Institute of Neurology, University College London, London, UK
7. Department of molecular neuroscience, UCL Institute of Neurology, Queen Square, London
8. UK Dementia Research Institute, London, UK
9. Clinical Neurochemistry Laboratory, Sahlgrenska University Hospital, Mölndal, Sweden
10. Department of Psychiatry and Neurochemistry, Institute of Neuroscience and Physiology, the Sahlgrenska Academy at the University of Gothenburg, Mölndal, Sweden
11. Rugby Football Union (RFU), London, UK
12. Arthritis Research UK Centre for Sport, exercise and osteoarthritis, University of Oxford, Oxford, UK
13. Department of Neuroscience and Trauma, Blizard Institute, Queen Mary, University of London, London, UK

Please address all correspondence to:

Dr Valentina Gallo

Centre for Primary Care and Public Health
Queen Mary, University of London
Yvonne Carter Building
58, Turner Street
E1 2AB London (UK)
valentina.gallo@lshtm.ac.uk or v.gallo@qmul.ac.uk

For peer review only

Abstract

Introduction - Relatively little is known about the long-term health of former elite rugby players, or former sportspeople more generally. As well as the potential benefits of being former professional sportspersons, there may be potential health risks from exposures occurring during the playing career, as well as following retirement. Each contact sport has vastly different playing dynamics therefore exposing its players to different types of potential traumas. Current evidence suggests that these are not necessarily comparable in terms of pathophysiology, and their potential long-term adverse effects might also differ. There is currently limited but increasing evidence that poorer age-related and neurological health exists among former professional sportsmen exposed to repetitive concussions; however the evidence is limited on Rugby Union players, specifically.

Methods and analysis – We present the protocol for a cross-sectional study to assess the association between self-reported history of concussion during the playing career, and subsequent measures of healthy ageing and subtle neurological and cognitive impairment. We are recruiting a sample of approximately 200 retired rugby players (former Oxbridge Blues and members of the England Rugby International Club) aged 50 years or more and collecting a number of general and neurological health-related outcome measures via validated tests. Biomarkers of neurodegeneration (neurofilaments and tau) will be also be measured. Although the study is focusing on rugby union players specifically, the general study design and the methods for assessing neurological health are likely to be relevant to other studies of former professional sportspersons.

Ethics and dissemination – The study has been approved by the Ethical Committee of London School of Hygiene and Tropical Medicine (LSHTM) (reference: 11634 [illegible] 2). It is intended that results of this study will be published in peer reviewed medical journals, communicated to participants, the general public, and all relevant stakeholders.

For peer review only

Keywords:

Rugby, concussion, Rugby Union, cross-sectional study, cognitive function, healthy ageing, neurological health, sport

For peer review only - http://bmjopen.bmj.com/site/about/guidelines.xhtml

Introduction

Some of the authors have previously reviewed the evidence relating head trauma in sport to the subsequent risks of neurological disease and have concluded that sports involving repeated head trauma may have an increased risks of neurodegenerative disease in the long-term (1). Furthermore, there are now plausible mechanisms for these effects, and a recognition that these problems do not just occur in former boxers, but in a variety of sports involving repeated concussions (2), and possibly also in sports in which low-level head trauma is common (3). These neurodegenerative effects potentially include increased risks of impaired cognitive function and dementia (4-7), Parkinson's disease (PD) (8-13), and amyotrophic lateral sclerosis (ALS) (3, 14-19). The term *chronic traumatic encephalopathy* (CTE) was introduced as a clinical-pathological construct for the neurodegeneration associated with American football and wrestling (20) (see Table 1). Seminal work by Ann McKee (21) investigating of CTE led to important considerations on the overlapping of neurodegenerative lesions in this condition (22). However, each contact sport has vastly different playing dynamics therefore exposing its players to different types of potential traumas. Current evidence suggests that these are not necessarily comparable in terms of pathophysiology, and hence in terms of their potential long-term adverse effects on health. There is currently limited but increasing evidence that poorer general and neurological health exists among professional sportsmen exposed to repetitive concussions; however, there is little evidence from rugby union players (23-25).

Decq et al (23) investigated retired French-speaking high-level sportsmen, aged 45 to 65 years, who had played sports for at least 10 years. Mild cognitive disorder (measured as score at the modified Telephone Interview for Cognitive Status (TICS-m) $\leq$30) was lower in players of other sports (40.4%) than in former rugby players (56.6%) (p=0.005). However, after adjustment for smoking and higher education, no association was observed between TICS score and number of reported concussions (23). Furthermore, in New Zealand, 366 former players were tested on their engagement in sport, general health, sports injuries and concussion history, and demographic information. Cognitive functioning was assessed using the online CNS Vital Signs neuropsychological test battery. The elite-rugby group performed worse on tests of complex attention, processing speed, executive functioning, and cognitive flexibility than the non-contact-sport group, and worse than the community-rugby group on complex attention. Former players who recalled one or more concussions had worse scores on cognitive flexibility, executive functioning, and complex attention than players who did not recall experiencing a concussion (24). A recent Scottish study assessed 52 former Scottish International rugby players and 29 controls. Players performed worst on a test of verbal learning and of fine co-ordination of the dominant

For peer review only - http://bmjopen.bmj.com/site/about/guidelines.xhtml

hand; however not significant differences on other cognitive tests were observed. No significant association was found between number of concussions and performance on cognitive tests (25).

Ultimately, resolving these issues will require long-term prospective studies involving the repeated measurement of various ‘exposures’ to head trauma, and repeated tests of neurological health in large numbers of current and former players. Furthermore, identifying the risks, if any, of severe neurological problems such as PD and ALS will require large numbers of participants and long periods of follow-up. However, a first step in this process is to conduct cross-sectional studies in former players to assess the history of concussion during their rugby career, and subsequent measures of healthy ageing and subtle neurological and cognitive impairment. In particular, such cross-sectional studies can be conducted in a relatively short time and with ‘standard’ measures of cognitive function since impaired cognitive function is an important health outcome in itself, and may be a precursor of more serious long-term neurological effects (26). Furthermore, cross-sectional studies of this type can also be conducted in current players, and thus form the basis for prospective studies measuring changes in neurological health over time.

Definitions of trauma and concussion

Traumatic brain injury (TBI) is usually classified as mild, moderate, or severe, on the basis of the initial Glasgow coma scale (GCS) (27) score recorded in the Accident and Emergency departments; the duration of loss of consciousness; and duration of post-traumatic amnesia (i.e., loss of memory of events after the injury) (28). Chronic TBI represents a spectrum of disorders associated with long-term consequences of single or repetitive TBI (29). Conversely, there is still no consensus on the definition of concussion. The 2012 Zurich Consensus Statement on Concussion in Sport proposed that concussion and mild TBI should be viewed as distinct entities (30). The group defined concussion as a “complex pathophysiological process affecting the brain”, and despite allowing for the presence of neuropathological damage, they postulated that concussive symptoms largely reflected a functional disturbance, typically resolving spontaneously with no imaging abnormality. In contrast, recent American Academy of Neurology guidelines for sports concussion in 2013 do not separate concussion from mild TBI, defining concussion as “a clinical syndrome of biomechanically induced alteration of brain function, typically affecting memory and orientation, which may involve loss of consciousness” (31). A recent report (32) dissects why having two different pathological entities might be unhelpful and

For peer review only - http://bmjopen.bmj.com/site/about/guidelines.xhtml

suggests that the Mayo Clinic TBI Classification system should cover both of them (33). A glossary of definitions used in this protocol is found in Table 1, adapted from Jordan (2014) (34).

For the purpose of this study, we adapted the NIH definition of concussion. Participants will be asked to report their previous concussions according with the following definition:

Concussion is defined as an alteration in brain function, caused by an external force. Symptoms include:

- *A decreased level / loss of consciousness*
- *Memory Loss (before or after the injury)*
- *Weakness*
- *Temporary Paralysis*
- *Loss of balance*
- *Change in vision (e.g. blurriness, double vision)*
- *Co-ordination difficulties*
- *Numbness*
- *Decreased sense of smell*
- *Difficulty understanding what others are saying*
- *Difficulty communicating with others*
- *Confusion, disorientation, or slowed thinking*

Please note, loss of consciousness is not required for a concussion to be diagnosed.

Impaired cognitive function and dementia in sportspersons

While the neurological and cognitive effects of acute traumatic brain injuries (TBI) have been extensively studied (4-7), the association between TBI and delayed sequelae have been less studied because of the variable latency period before overt neurologic dysfunction, and the difficulty of retrospectively recalling relevant events (recall bias), in particular in presence of memory impairment. The neurocognitive effects of repetitive mild head injury in sport were initially recognised in boxers, a syndrome that was distinct from the clinical and pathological sequelae of single-incident severe TBI (35) (see also Table 1). The clinical syndrome of dementia pugilistica (punch-drunk syndrome) is associated with prominent tauopathy, with typical neurofibrillary tangles and neuropil threads, distributed in patches throughout the neocortex (36). Neuropathological case series have demonstrated primarily tau-related pathology in

For peer review only - http://bmjopen.bmj.com/site/about/guidelines.xhtml

For peer review only

the brains of individuals who have suffered from a clinical syndrome encompassing dementia and movement disorders after repeated head trauma (37). Also, post-mortem examination of the brains of several professional American football players and wrestlers has revealed the pathological underpinnings for the cognitive and neuropsychiatric decline seen in these men in later life (20). Although cognitive decline in long time professional American football players has been noted for some years, the first autopsy report from such a player appeared in the literature only recently (38). In all cases, cognitive decline began years after retirement from the game.

Cognitive function is an ideal outcome measure for this type of study as being a continuous measure maximizes power to detect an association; for using neurodegenerative disease as outcome measures a substantial larger sample would be needed. Cognitive function as measured with neurocognitive tests is also an aspecific measure, does not imply a clinical diagnosis, and it can be the prodrome manifestation for a number of different neurodegenerative diseases.

Biomarkers

Research in the field of TBI biomarkers has increased exponentially over the last 20 years (29, 39-41), with studies assessing biomarkers in TBI including markers that could provide diagnostic, prognostic, as well as monitoring information. S100β, glial fibrillary acidic protein (GFAP), neuron-specific enolase (NSE), tau, neurofilament light protein (NF-L), amyloid beta, brain-derived neurotrophic factor (BDNF), serum creatinine kinase (CK), and heart-type fatty acid binding protein (hFABP), prolactin, cortisol, and albumin are all biomarkers more frequently studied in relation to sport-related concussion (42, 43). However, to date, no biomarker of long-term effects of concussion has been identified, although recent studies on cerebrospinal fluid (CSF) from sportsmen with post-concussion syndrome suggest that a subset of these have biomarker signs of ongoing axonal injury and microglial activation (44-46).

Of the biomarkers measurable in serum or other body fluids, those more likely to be seen a long time after the concussion episode, are those related to the axonal injury and more in generally neuroinflammation, that is neurofilament and tau protein (43). Neurofilaments (NF) co-assemble from protein subunits to form NFs defined as light (light (NF-L), medium (NF-M), or heavy (NF-H) according to their relative molecular weights). NFs are one of the key structural elements of neurons, providing mechanical stability and determining axonal diameter. NFs are of particular interest because they are structural elements and therefore they might be more susceptible to mechanical deformation of the

For peer review only - http://bmjopen.bmj.com/site/about/guidelines.xhtml

brain tissue under condition of trauma. Abnormal NF aggregation may contribute to delayed or progressive neuronal death and dysfunction taking place in neurodegenerative diseases associated with NF aggregation, such as PD, ALS, and Lewy body dementia (LBD) (47-49). Acute perturbations of NFs have been demonstrated in experimental models of TBI that produce cortical contusion in combination with selective hippocampal neuronal death (50). A study compared serum NF-L, a biological marker of head trauma, in American football athletes with non-contact sport athletes and examined changes over the course of a season. Results suggest that a season of collegiate American football is associated with elevations in serum NF-L, which is indicative of axonal injury, as a result of head impacts (51). NF-L have also been associated with head trauma severity detected by CT scan, immediately after the episode (52) and with concussive and subconcussive head blows in boxing (46). Importantly, whilst plasma tau concentration increases and disappears rapidly (within hours to a few days) following concussion (53), NF-L has a prolonged increased levels lasting for many weeks (46, 54, 55).

Phosphorylation of tau is a normal event in healthy neurons, but hyperphosphorylation and aggregation into neurofibrillary tangles is a characteristic of Alzheimer's disease and CTE (56). Tau concentrations correlate with lesion size and outcome in severe TBI when measured in the ventricular CSF, while it is unchanged in TBI and other forms of acute brain injury, when measured CSF (43). Studies on mild TBI show increased CSF concentrations of both t-tau and NF-L, although the increase in CSF NF-L is greater than the increase in t-tau, suggesting that blows to the head have a greater impact on long, large-calibre axons that extend subcortically than on short, nonmyelinated axons in the cortex (43). After concussion, serum tau concentrations were found to be increased, but this increase did not correlate with severity of trauma and lesion load as measured using CT scan signs (43).

Genetics

A number of genes are involved in modulating the risk of developing dementia and other neurodegenerative diseases. Dementia risk is higher among the carriers of the epsilon4 allele at the Apolipoprotein E (APOE) gene (57). A number of GWASs have been performed so far enabling the identification of 24 loci as Parkinson's disease risk factors. These loci take part in numerous cellular processes that may contribute to PD pathology: protein aggregation (e.g. *SNCA* coding for α-synuclein; and *MAPT* encoding a microtubule-associated protein tau), protein, and membrane trafficking (e.g. *LRRK2* (leucine-rich repeat kinase 2) gene), lysosomal autophagy (e.g. GBA (beta acid glucosidase)), immune response (e.g. *BST1* (bone marrow stromal cell antigen-1)), synaptic function, endocytosis,

For peer review only

For peer review only - http://bmjopen.bmj.com/site/about/guidelines.xhtml

inflammation, and metabolic pathways are among the most important ones (58). The identified single nucleotide polymorphisms are usually located in the non-coding regions and their functionality remains to be determined, although they presumably influence gene expression. Similarly, since the discovery of mutations in SOD1 gene, which account for ~2% of ALS cases, increasing efforts have been made to understand the genetic component of ALS risk. Specifically mutation in the chromosome 9 open reading frame 72 (C9orf72) were associated to both ALS and Fronto-Temporal Dementia (FTD) (59).

Rationale of the BRAIN Study

Recently, World Rugby (formally known as the International Rugby Board), have developed a process to support team doctors in the recognition, assessment, and subsequent management of elite adult players who sustained a potential concussion. This process includes the development of a multimodal assessment: the Head Injury Assessment, formerly the Pitch Side Concussion Assessment (PSCA) tool (60).

However, there is little clear evidence, about the possible long-term effects of concussion in rugby union players and it is not clear if the findings from other sports are directly generalizable to rugby players, per se. Many sports involve concussion or repetitive low-level head trauma, but it can be argued that each sport should be viewed differently depending on the unique technical and physiological profile that a player is exposed to over the course of a career (61, 62). Thus, to determine the risk of long-term adverse health effects of playing rugby, in addition to the other research that is ongoing in different contact sports, specific studies of rugby players are needed. The BRAIN Study builds upon a recent questionnaire-based study conducted by members of the Arthritis Research UK sport, exercise and osteoarthritis centre, University of Oxford, which has assessed the general and musculoskeletal health of former elite rugby players. Rugby players from the Oxford and Cambridge University Football Clubs (Oxbridge Blues), and members of the England Rugby Internationals Club (ERIC) - an honorary association of all England and ex-England players - were recruited. Self-reported information on demographic factors, playing history (including head trauma), past medical history (including dementia, depression, and memory impairment), and perceived health were collected, in addition to detailed information on musculoskeletal health and pain.

For peer review only - http://bmjopen.bmj.com/site/about/guidelines.xhtml

A total of 320 participants have been surveyed in this study to date, and 205 of those aged 50 years or older have agreed to be re-contacted for further testing/questionnaires. The 205 participants aged 50+ years who agreed to be re-contacted will be invited to take part to the BRAIN Study.

Aim and objectives

The general aim of the BRAIN study is to investigate the possible associations between concussion in rugby and ageing – including physical and cognitive capabilities – as well intermediate neurological and musculoskeletal endpoints among former rugby players.

In order to achieve these aims the following objectives will be pursued:

1. Investigate the associations between self-reported concussion history and ageing, measured as physical and cognitive capabilities. This will be achieved by using the following outcome measures:
    a. Physical capability outcomes: grip strength, chair raise and walking speed
    b. Cognitive capability outcomes: memory, reasoning, speed of thinking and attention, and verbal and numerical skills.
2. Investigate the association between self-reported concussion history and intermediate neurological endpoints
    a. Neurological examination: a brief neurological examination will be video-recorded according to existing protocols and independently examined by two neurologists
    b. Intermediate neurological endpoints: information using tapping test (BRAIN), smell test, REM-behaviour disorder questionnaire will be collected in order to explore non motor symptoms of Parkinson's disease and related disorders
3. Investigate whether a history of concussion is associated with current tau protein and NF-L levels in blood (potential biomarkers of neurodegeneration), and how these biomarkers are in turn associated with the outcome measures (potential biomarkers of early detection of disease)
4. Assess if any other characteristic of rugby playing history, in addition to self-reported concussion (i.e. length of playing at elite level, role played, numbers of game played, age when started playing), or age at concussion is associated with any outcome measure (physical and cognitive capability and/or intermediate neurological outcomes)

For peer review only - http://bmjopen.bmj.com/site/about/guidelines.xhtml

5. Investigate the long-term musculoskeletal health outcomes of rugby players with particular emphasis on hip, knee, and hand osteoarthritis allowing changes over time (from the previous study to the current study) to be assessed

In addition, we will build a multimedia database (data from tests and questionnaire plus video-recorded neurological examination plus biobank of blood samples) that will serve as baseline for further tests and further follow-up if this is considered appropriate in the future

Study design

Participants will be invited to study clinics in London, Manchester, or Bath. For those who would prefer, a home visit will be arranged. Participants will be invited to bring with them any medication they are taking regularly for a more accurate recording of all medications.

All participants will be administered a ***Core Module*** interview including questions on lifestyle factors, potential confounders, and extensive information on the five domains of physical and cognitive ability, neurological examination, intermediate neurological outcomes and musculoskeletal health.

The lifestyle questionnaire will complement that already administered by our colleagues in Oxford, and will include questions on potential confounders of the association between concussion and physical and cognitive capability, and intermediate neurological outcomes. Participants will also be asked to donate a blood sample (a normal blood sample will be collected by participants seen in one of the clinics, a dry spot sample plus saliva swab for DNA will be collected from participants seen at home). At the end of the Core Module, all participants will be also asked to undergo some additional tests (the ***Additional Optional Module***) providing they have sufficient time (Figure 1). At the end of the interview, all participants will be asked some final questions on their concussion history. This will ensure that the interviewer is blind to the participant's concussion history during the entire duration of the interview and this will also act as validation check on data previously collected.

The ***Core Module*** includes: 1) a lifestyle questionnaire; 2) a set of tests covering essential information on physical (height, weight, grip strength, chair rise, walking speed and photo of the hands) and cognitive capability (Mini-Mental State Examination, Logical Memory, Digit-Symbol Substitution test, Matrix Reasoning, Task-set Shifting/Response Inhibition, Visuomotor Integration, 12-item Face-Name Associative Memory Exam, and the National Adult Reading Test); 3) a remote neurological clinical

For peer review only - http://bmjopen.bmj.com/site/about/guidelines.xhtml

examination (video recorded); and 4) a test for subtle movement disorders, the BRAIN tapping test. This Core Module should take no longer of 1 hour and 45 minutes to be completed (**Error! Reference source not found.**).

The ***Additional Optional Module*** includes: 1) a questionnaire investigating hand pain; 2) extra tests investigating the cognitive domain (Visual short-term memory binding, Irrelevant Distractor Paradigm); 2) the UPDRS part II scale to complete the neurological examination (not video-recorded); 3) additional tests for intermediate neurological outcomes (the smell test and the REM behaviour disorder questionnaire). This Additional Optional Module should take no longer than 1 hour and half to be completed (Figure 1 and **Error! Reference source not found.**).

General Questionnaires

Questionnaires will be used to collect relevant information from the participants.

Lifestyle and confounder questionnaire – all participants will be asked a number of questions on their lifestyle in order to collect information on possible confounders for the main analysis (smoking, alcohol, coffee, drugs, past medical history, sleep quality).

Concussion history questionnaire – at the end of the interview, participants will be re-asked some detailed information regarding their history of head trauma and concussion when playing.

Hand pain – A questionnaire on hand pain to identify possible hand osteoarthritis will be used using a hand mannequin (additional optional module).

Physical ability assessment

The methodology used in the 1946 birth cohort (63) will be leveraged to assess physical and cognitive capabilities in this study. This will enhance comparability of results and facilitate future collaborations. The most commonly used objective measures of physical capability for assessing healthy ageing, are tests of grip strength (64, 65), walking speed (64, 66, 67), chair rises (68) and standing balance (64); these aim to assess physical functioning, including the capacity to undertake the physical tasks of daily living (69). There is robust evidence that higher scores on these measures are associated with lower rates of mortality, and there is more limited evidence of lower risks of morbidity, and of age-related patterns of change (69).

For peer review only - http://bmjopen.bmj.com/site/about/guidelines.xhtml

For peer review only

Height and weight – height and weight will be collected in order to calculate the body mass index (BMI) according to the formula BMI = weight (Kg) / height $(m)^2$.

Grip strength - JAMAR Hydraulic Hand Dynamometer is the most widely used instrument with established test-retest, inter-rater and intra-rater reliability (70).

Chair rise - The 30-Second Chair Stand Test will be used to assess lower body strength (71). This test provides a reasonably reliable and valid indicator of lower body strength in generally active, community-dwelling older adults. Test-retest intra-class correlations of 0.84 for men and 0.92 for women, utilizing one-way analysis of variance procedures appropriate for a single trial, together with a non-significant change in scores from Day 1 testing to Day 2, indicate that the 30-s chair stand has good stability reliability (71). Moreover, a moderately high association between chair-stand performance and maximum weight-adjusted leg-press performance for both men and women (r = 0.78 and 0.71, respectively) supports the criterion-related validity of the chair stand as a measure of lower body strength (71). As expected, chair-stand performance decreased significantly across age groups in decades – from the 60s to the 70s to the 80s ($p < 0.01$) and was highly statistically significantly lower for low-active participants than for high-active participants ($p < 0.0001$) (71).

Walking speed – To assess normal comfortable walking speed and maximum walking speed, a Timed 10-Meter Walk Test will be used. This test has been validated in 230 healthy volunteers (66). Mean comfortable gait speed ranged from 127.2 cm/s for women in their seventies to 146.2 cm/s for men in their forties. Mean maximum gait speed ranged from 174.9 cm/s for women in their seventies to 253-3 cm/s for men in their twenties. Both gait speed measures were reliable (correlation coefficients > 0.903) and correlated significantly with age (r>-0.210), height (r>0.220) and the strengths of four measured lower extremity muscle actions (r = 0.190-0.500). These normative values give clinicians a reference against which patient performance can be compared in a variety of settings (66).

A photograph of the hand will be taken at the end of this section.

HOOS – The Hip Disability and Osteoarthritis Outcome Score (HOOS) measures patient's opinions about their hip and associated problems. It examined pain, symptoms, function in activities of daily living (ADL) and function in sport and recreation and has been used in subjects with hip disability with or without hip Osteoarthritis (OA) (72) (Additional optional module).

For peer review only - http://bmjopen.bmj.com/site/about/guidelines.xhtml

KOOS – The Knee Injury and Osteoarthritis Outcome Score (KOOS) examines pain frequency and severity during functional activities, symptoms such as the severity of knee stiffness and the presence of swelling, grinding or clicking, difficulty in ADL, with sport and recreation, and knee-related quality of life (QOL). It is intended for use in young and middle-aged populations with post-traumatic OA, in addition to those with injuries who may go on to develop secondary OA (73) (Additional optional module).

QuIKS – The Questionnaire to Identify Knee Symptoms (QuIKS) is aimed at early osteoarthritis, and understanding symptomology and potential adaptation to activity before osteoarthritis has been diagnosed. It has been produced in adults aged 40-65 with evidence of ongoing knee problems and recommended for use in studies exploring early osteoarthritis (74) (Additional optional module).

Cognitive ability assessment

Also for the cognitive capability assessment, the methodology used in the 1946 birth cohort will be followed (63). Tests and questionnaires will be described according their belonging to the Core Module or the Additional Optional Module, and by domain (Figure 1).

Mini-Mental State Examination (MMSE (75)) – The MMSE is a widely used 30-point screening tool for cognitive impairment within clinical practice, assessing multiple cognitive domains including: i) orientation to time and place (10 points); ii) registration (3 points), which involves repeating back to the examiner three items; iii) attention +/- calculation (5 points), which involves the participant spelling "WORLD" backwards or performing a serial subtraction task; iv) recall (3 points), which involves recalling the originally registered three items two minutes later; v) language (1 point) which involves a two item naming tasks; vi) repetition (1 point); vii) reading (1 point), which involves following a written command; viii) writing (1 point), which involves writing a grammatically correct sentence; ix) visuospatial function (1 point), tested by drawing interlocking pentagons; and x) following a 3-stage command (3 points), which assesses language function, executive function and praxis.

Logical Memory from the ***Wechsler Memory Scale-Revised*** (WMS-R (76)) - The Logical Memory test assesses free recall of a short story. The participant is asked to recall the story immediately and after a 20minute delay.

12-item Face-Name Associative Memory Exam (FNAME-12A) – The FNAME-12A is a modified version of the 16-item Face-Name Associative Memory Exam (FNAME-16). The FNAME-12A has fewer stimuli and additional learning trials which have been found to be well tolerated by those with Mild Cognitive

For peer review only - http://bmjopen.bmj.com/site/about/guidelines.xhtml

Impairment, whilst remaining challenging in cognitively normal older adults (77). The FNAME-12A has demonstrated psychometric equivalence with the FNAME-16, which has been shown to be related to beta-amyloid burden in cognitively normal elderly people (78). The FNAME-12A requires the participant to learn 12 face-name and face-occupation pairs. Participants are given two exposures to all 12 face-name/occupation pairs. After each exposure and following a 5 minute delay they are asked for name and occupation associated with each face. After a 30 minute delay they are shown 3 faces and asked to identify the face that they recognise and give the name and occupation. Given multiple options to pick from, they are then asked to select the name and/or occupation associated with the face.

Task-set Shifting/Response Inhibition (79, 80) – The task-set shifting and response inhibition task examines the relationship between executive tasks of task-switching/preparation time. In the arrow only condition, participants are shown the cue 'arrow'. Following a short delay they must respond to the direction of the arrow ('right' or 'left'). In the word only condition, participants are shown the cue 'word'. Following a short delay they must respond to the direction of the word ('right' or 'left'). There is a switching condition in which the participant is shown the cue 'arrow' or 'word'. Following a delay both a combined arrow and word stimulus appears. The stimulus is either congruent (left arrow and left word), or incongruent (left arrow and right word). Trials in the switching task are categorised into switch and non-switch. In a non-switch trial the cue is the same as for the immediately preceding trial. In a switch trial the cue differs from the immediately preceding trial.

Digit-Symbol Substitution Test, from the ***Wechsler Adult Intelligence Scale-Revised*** (WAIS-R (81)) - The Digit-Symbol Substitution test explores attention and psychomotor speed. Participants are given a code table displaying digits (from 1 to 9), each digit is paired with a symbol. The participant is required to fill in blank squares with the corresponding symbol for each digit as shown in the code table. They are given 90 seconds to fill in as many squares as possible.

Visual Short-term Memory Binding (82, 83) – This test requires the participant to view one or three fractal objects, presented simultaneously in random locations on the screen. The participant is asked to remember both the objects and their location. After a delay of 1 or 4 seconds they have to make a forced choice between one of the displayed fractals (the target) and a 'dummy' fractal. Participants are required to touch the object they think has been previously presented and 'drag' it on the touch screen to its remembered, original location. The binding of such featured information has been shown to be vulnerable in asymptomatic FAD mutation carriers (83).

For peer review only - http://bmjopen.bmj.com/site/about/guidelines.xhtml

National Adult Reading Test (NART) – The NART was specially designed to provide a means of estimating the premorbid intelligence levels of adult suspected of suffering from intellectual deterioration (84). The NART comprises a list of 50 words printed in order of increasing difficulty. The words are relatively short in order to avoid the possible adverse effects of stimulus complexity on the reading of dementing subjects, and they are all 'irregular' with respect to the common rules of pronunciation in order to minimise the possibility of reading by phonemic decoding rather than word recognition (84).

Visuo-motor Integration (85) – This is a circle tracing task which includes both direct and indirect visual feedback conditions. Continuous performance measures are provided including accuracy, speed and speed of error detection and correction. The test has revealed changes in speed and accuracy in Huntington Disease mutation carriers more than 10 years before expected age-of-onset (Additional optional module).

Matrix Reasoning from the ***Wechsler Abbreviated Scale of Intelligence*** (WASI (86)) – The Matrix Reasoning test assesses nonverbal reasoning. The participant is shown a matrix of geometric shapes with a section missing. They are required to select the option that completes the matrix (Additional optional module).

Irrelevant Distractor Paradigm (87) – Participants are given a computerised letter-search task and are required to make a rapid decision as to whether the target letter 'X' or 'N' has appeared in the search display (in either low or high load conditions). On some of the trials, a task-irrelevant distractor (a cartoon character) appears on the outside of the search display. The task evaluates the extent to which attention is captured and captivated by the distractor (Additional optional module).

For peer review only

Neurological clinical assessment

The neurological assessment of participants is needed for detecting any subtle neurological sign, and/or to set a baseline for the absence of one or more signs. A video-recorded standard neurological examination will be included as part of the Core Module. An additional test of self-reported impairment mainly due to movement disorders will be administered as part of the Additional Optional Module (Figure 1).

A standard video recording of each participant accessing the study at one of the clinics will be performed and evaluated by a clinical neurologist. The assessment includes examination of strength,

For peer review only - http://bmjopen.bmj.com/site/about/guidelines.xhtml

coordination, balance, ocular movements, cranial nerves, gait, and repeated movements. The examination includes also a test of knee functionality (88).

Information on potential movement disorders will be collected using the unified Parkinson's Disease Rating Scale (PDDRS) part II. This includes self-reported ratings on several motor domains including speech, saliva and drooling, chewing and swallowing, eating, dressing, hygiene, handwriting, hobbies, turning in bed, tremor, standing up, walking and balance, and freezing (Additional optional module).

Intermediate neurological endpoints

In addition to neurological signs and symptoms, some intermediated outcomes have been recognised as part of the complex clinical picture of movement disorders manifesting before the onset of the movement impairment itself. These will be investigated with three tests described below (Figure 1).

BRAIN tap test –This is a simple and rapid computerised keyboard test, based on the alternating finger tapping test, which has been developed to quantify upper limb motor function (89). The test generates several variables: 1) kinesia score: the number of keystrokes in 60 seconds; 2) akinesia time: cumulative time that keys are depressed; 3) dysmetria score: a weighted index calculated using the number of incorrectly hit keys corrected for speed; 4) in-coordination score: a measure of rhythmicity which corresponds to the variance of the time interval between keystrokes (89). The BRAIN TEST provides a simple, rapid, and objective assessment of upper limb motor function. It assesses speed, accuracy, and rhythmicity of upper limb movements regardless of their physiological basis. The results of the test correlate well with clinical rating scales in Parkinson's disease and cerebellar dysfunction. The BRAIN tap test is useful in clinical studies (89). The BRAIN tap test is available online at https://www.braintaptest.com/en_GB and V Gallo has obtained access for generating tokens for administering the test to participants (A Noyce, personal communication)

University of Pennsylvania Smell Test – This is a measurement of the individual's ability to detect odours at a supra-threshold level (90). The test takes only a few minutes. The test consists of 4 different 10 page booklets, with a total of 40 questions. On each page, there is a different "scratch and sniff" strip which is embedded with a microencapsulated odorant. There is also a four choice multiple choice question on each page. The scents are released using a pencil. After each scent is released, the patient smells the level and detects the odour from the four choices. There is an answer column on the back of

For peer review only - http://bmjopen.bmj.com/site/about/guidelines.xhtml

For peer review only

the test booklet, and the test is scored out of 40 items. The score is compared to scores in a normative database from 4000 normal individuals (91) (Additional optional module).

The REM-Behaviour Disorder Screening Questionnaire (RBDSQ) – This test is a validated 10-item patient self-rating questionnaire (maximum total score 13 points) covering the clinical features of REM Behavioral Disorders RBD (92). The RBDSQ was validated on 54 patients with polysomnographically confirmed RBD and 160 control subjects in whom RBD was excluded by history and polysomnography and 133 unselected healthy subjects. Applying a positivity threshold of 5 points, the RBDSQ had a sensitivity of 0.96 and a specificity of 0.56 comparing patients with RBD vs. patients with other sleep disturbances. A specificity of 0.92 was calculated when comparing patients with RBD vs. patients without sleep disturbances (92) (Additional optional module).

Blood and saliva sample collection, and bio-banking

Participants seen in the research clinics will be asked to donate a blood sample for testing for tau protein, neurofilament levels, full blood count analysis and to obtain data for a Genome Wide Association Study (GWAS). Samples of serum, plasma, and DNA will be collected and processed according to a pre-specified protocol and stored in a freezer at -80 °C at the Blizard Institute (Queen Mary, University of London). All participants will also asked to donate a blood sample through a dried blood spot test and a saliva sample (in order to get DNA if it is not possible to do a phlebotomy).

Samples will be tested to measure the expression of NF-L, a Nf-subunit showing consistent and reproducible measurements in plasma/serum using a newly developed sensitive methodology exploiting single molecule trapping and measurement by ELISA (SIMOA) (93-95). The use of both NFH and NFL allows for the determination of different markers whose dynamic of release into bio fluids following injury vary according to their particular chemical structure. NfL has shown good levels of linearity on dilution experiments suggesting that confounders like aggregation or immune response may not interfere with its measurements. NfH different phosphorylation states in blood may in turn provide more information on the systemic biological changes induced by trauma. The relative low abundance of these proteins in blood, in absence of CSF, which is normally more enriched of by-products of neuronal destruction, will not represent a major obstacle as analytical sensitivity has evolved with the development of novel assays which cover the lower end of these protein dynamic range. SIMOA, the proposed methodology for the target biomarkers, is now emerging as the core technique for the

For peer review only - http://bmjopen.bmj.com/site/about/guidelines.xhtml

measurement of structural proteins down to the fempto-molar end of the spectrum. A novel SIMOA-based assay for the measurement of Tau in blood is now available. This development opens a wide range of opportunities based on the possibility of overcoming the limitations imposed by CSF collection by lumbar puncture.

GWAS will be conducted at UCL in the lab led by Prof John Hardy.

Multimedia database set up and management

A multimedia database will be set up as part of this project. The final dataset will include data collected through the questionnaire and the tests (see Figure 1), and the video-recorded neurological examination. This data will be also linked to the stored blood samples through a unique identifier.

The data will be stored in mirror, anonymized datasets at LSHTM and QMUL on secure servers, access will be regulated by the study PIs who are the ultimate responsible for maintaining confidentiality and data protection. Only the researcher(s) employed on this study will have access to subject identifiable information. Identifiable information (name, DOB, and address) will be removed prior to analysis and subjects will be identified by pseudoanonymised codes. Personal data and key codes for pseudoanonymisation will be stored in a database with restricted access. Before conducting the statistical analyses, a number of checks will be run to ensure high quality of data.

Statistical analysis

Standard descriptive statistical methods (geometric means, geometric standard deviations, and 95% Confidence Intervals (95% C.I.) will be used to summarise the rugby history and lifestyle exposure data. Analyses linking history of head trauma with neurological symptoms (obtained by questionnaire and neurobehavioural tests) will be then performed. Parameters of physical and cognitive capability alongside with clinical neurological intermediated endpoints will initially be dichotomised (yes/no symptoms), therefore analysed with prevalence odds ratios using logistic regression (96). We will also analyse continuous outcome measures using multiple linear regression. In addition to analysing individual symptoms, we will assess associations with domains of symptoms (i.e. physical and cognitive capability). For this purpose we will use cut-points previously published in the international literature (97) particularly related to the 1946 Birth Cohort whose testing protocol was used. For those analyses

For peer review only - http://bmjopen.bmj.com/site/about/guidelines.xhtml

involving repeated measurements (neurobehavioural testing) assessing acute and chronic effects we will use multilevel models. Analyses will be adjusted for age, sex, and alcohol consumption.

Finally, we will compare neurological outcomes assessed by questionnaire with those assessed by computer-administered tests using Kappa-statistics (and in case of continuous outcomes linear regression analyses). We will also compare the strengths of the associations with exposure between both outcome measures.

Study size and power

We will invite about 205 participants to participate, half of whom have been exposed to concussion during their career (M Cross, personal communication). We conservatively assume that about 150 will participate. Based on previous studies, the standard deviations of the psychometric tests are in the range of 8-15% of the absolute value; assuming a conservative figure of 15% overall, the study will have more than 95% power to detect a 10% difference, and 80% power to detect a 7% difference, in psychometric test scores, between exposed (to concussion) and non-exposed participants.

Limitations

The main limitation of this study is the cross-sectional design. Information on exposure (history of concussion) and health outcomes is collected at one point in time making data subject to recall bias. Participants with a worst health might be prone to recollect more precisely the number of concussions they suffered if they blame them for their current poor health status.

However, this type of study design will provide us with meaningful data on the burden of ill-health and neurological ill-health among retired rugby players in only a two-year time frame. Depending on results (and availability of additional funding), this study could inform a better, more detailed, but more time-consuming cohort study aimed at assess the association between concussion in rugby players and health outcomes prospectively.

One of the direct consequences of the cross-sectional design is also an intrinsic risk of selection bias. Rugby players who have developed serious neurodegenerative conditions (or have died from them) in the meantime are less likely (or not able) to participate in the study thus limiting our sample to a subsample of healthier retired rugby players. However, this won't impede investigating the association between exposure to concussion and general and neurological health, despite the fact that a smaller

For peer review only - http://bmjopen.bmj.com/site/about/guidelines.xhtml

range of health outcomes will diminish the power for detecting an association as significant. Nonetheless, the power of this study is calculated on the basis of continuous outcome measures (i.e. neurocognitive tests or grip strength measurement) making this study adequately powered to detect the planned associations based on the expected number of recruited participants.

Also, it is worth noting that results from this study will be not immediately apply to current rugby players as in the last 30 years or so as playing rules and conditions have been changed to increase player safety.

Ethics and dissemination

Ethical approval has been sought from the London School of Hygiene and Tropical Medicine (LSHTM) Ethics Committee. The study in general is focussing on subclinical problems (e.g. slightly lower than average scores in cognitive function tests) which would not usually require any medical attention. If any problems are identified we will (with the permission of the study participant) refer them to their GP. The consent form includes the following statement:

- ☐ *I wish to inform my GP that I am taking part to this study*
- ☐ *I wish the study team to inform my GP of any unusual test result*

This study will provide a unique snapshot of physical and neurological health of retired rugby players who are now aged 50 years or more. This is valuable and precious information *per se*, potentially comparable with other occupational cohorts or the general population (accepting the possible bias discussed previously in comparing these samples). Moreover, within this study, data will be analysed in as a function of the history of traumatic brain injury and concussion during their careers so in order to assess associations between history of injuries and health-related outcomes.

Importantly, the neurological examination and the collection of information on intermediate neurological outcomes allow detection of subtle preclinical changes which might be associated with future risk of developing a neurodegenerative condition. Studying to what extent these changes are also associated with history of concussion would allow estimate the impact of concussion on the onset of neurodegenerative diseases in Rugby players posing thus providing the basis for future prospective studies.

For peer review only - http://bmjopen.bmj.com/site/about/guidelines.xhtml

A novel aspect of this project is the molecular component: clinical and neurocognitive findings will be particularly reinforced by the measurement of blood biomarkers. These will give a quantitative measurement of active neurodegeneration (NfL) and of possible CTE (tau) to be analysed conjunctly with the health-related outcomes and personal history of concussion.

Also, relying on a number characteristics of the rugby playing history collected (i.e. length of playing at elite level, role played, numbers of game played, age when started playing) it will be possible to identify potential categories of players at potentially increased risk of health related outcomes.

What this study is likely to identify (if it finds any effect at all) is a small deviation from normality on any of the continuous measures collected via cognitive and other tests (i.e. grip strength, memory function, walking speed, etc). This will be mainly investigated in association with a history of frequent concussions or other head traumas. The investigators will base the interpretation of the results on the continuum of distribution of the test outcomes, which do not have a clinical meaning *per se*. However, it should be stressed that although this study is unlikely to identify patterns suggesting a clinical disease in any of the participants, if these are identified, then the relevant participant(s) would be referred to appropriate medical services.

Conclusions

Here we have here presented the protocol for a cross-sectional study of history of concussion during a rugby union career, and subsequent measures of healthy ageing and subtle neurological and cognitive impairment. Although the study is focusing on rugby players, the general study design, and the methods for assessing neurological health, are likely to be relevant to other studies of former professional sportspersons. Furthermore, cross-sectional studies of this type can also be conducted in current players, and thus form the basis for prospective studies measuring changes in neurological health over time.

For peer review only - http://bmjopen.bmj.com/site/about/guidelines.xhtml

References

1. Pearce N, Gallo V, McElvenny D. Head trauma in sport and neurodegenerative disease: an issue whose time has come? Neurobiol Aging. 2015;36(3):1383-9.
2. Lehman EJ. Epidemiology of neurodegeneration in American-style professional football players. Alzheimers Res Ther. 2013;5(4):34.
3. Chio A, Benzi G, Dossena M, Mutani R, Mora G. Severely increased risk of amyotrophic lateral sclerosis among Italian professional football players. Brain. 2005;128(Pt 3):472-6.
4. Belanger HG, Vanderploeg RD. The neuropsychological impact of sports-related concussion: a meta-analysis. J Int Neuropsychol Soc. 2005;11(4):345-57.
5. Kokmen E, Beard CM, O'Brien PC, Kurland LT. Epidemiology of dementia in Rochester, Minnesota. Mayo Clin Proc. 1996;71(3):275-82.
6. Nemetz PN, Leibson C, Naessens JM, Beard M, Kokmen E, Annegers JF, et al. Traumatic brain injury and time to onset of Alzheimer's disease: a population-based study. Am J Epidemiol. 1999;149(1):32-40.
7. Schofield PW, Tang M, Marder K, Bell K, Dooneief G, Chun M, et al. Alzheimer's disease after remote head injury: an incidence study. J Neurol Neurosurg Psychiatry. 1997;62(2):119-24.
8. Harris MA, Shen H, Marion SA, Tsui JK, Teschke K. Head injuries and Parkinson's disease in a case-control study. Occup Environ Med. 2013;70(12):839-44.
9. Rugbjerg K, Ritz B, Korbo L, Martinussen N, Olsen JH. Risk of Parkinson's disease after hospital contact for head injury: population based case-control study. BMJ. 2008;337:a2494.
10. Goldman SM, Tanner CM, Oakes D, Bhudhikanok GS, Gupta A, Langston JW. Head injury and Parkinson's disease risk in twins. Ann Neurol. 2006;60(1):65-72.
11. Seidler A, Hellenbrand W, Robra BP, Vieregge P, Nischan P, Joerg J, et al. Possible environmental, occupational, and other etiologic factors for Parkinson's disease: a case-control study in Germany. Neurology. 1996;46(5):1275-84.
12. Kuopio AM, Marttila RJ, Helenius H, Rinne UK. Environmental risk factors in Parkinson's disease. Mov Disord. 1999;14(6):928-39.
13. Bower JH, Maraganore DM, Peterson BJ, McDonnell SK, Ahlskog JE, Rocca WA. Head trauma preceding PD: a case-control study. Neurology. 2003;60(10):1610-5.
14. Lehman EJ, Hein MJ, Baron SL, Gersic CM. Neurodegenerative causes of death among retired National Football League players. Neurology. 2012;79(19):1970-4.
15. Savica R, Parisi JE, Wold LE, Josephs KA, Ahlskog JE. High school football and risk of neurodegeneration: a community-based study. Mayo Clin Proc. 2012;87(4):335-40.
16. Pupillo E, Messina P, Giussani G, Logroscino G, Zoccolella S, Chio A, et al. Physical activity and amyotrophic lateral sclerosis: a European population-based case-control study. Ann Neurol. 2014;75(5):708-16.
17. Belli S, Vanacore N. Proportionate mortality of Italian soccer players: is amyotrophic lateral sclerosis an occupational disease? Eur J Epidemiol. 2005;20(3):237-42.
18. Longstreth WT, McGuire V, Koepsell TD, Wang Y, van Belle G. Risk of amyotrophic lateral sclerosis and history of physical activity: a population-based case-control study. Arch Neurol. 1998;55(2):201-6.
19. Scarmeas N, Shih T, Stern Y, Ottman R, Rowland LP. Premorbid weight, body mass, and varsity athletics in ALS. Neurology. 2002;59(5):773-5.
20. DeKosky ST, Ikonomovic MD, Gandy S. Traumatic brain injury--football, warfare, and long-term effects. N Engl J Med. 2010;363(14):1293-6.

For peer review only - http://bmjopen.bmj.com/site/about/guidelines.xhtml

21. McKee AC, Cantu RC, Nowinski CJ, Hedley-Whyte ET, Gavett BE, Budson AE, et al. Chronic traumatic encephalopathy in athletes: progressive tauopathy after repetitive head injury. J Neuropathol Exp Neurol. 2009;68(7):709-35.
22. McKee AC, Stern RA, Nowinski CJ, Stein TD, Alvarez VE, Daneshvar DH, et al. The spectrum of disease in chronic traumatic encephalopathy. Brain. 2013;136(Pt 1):43-64.
23. Decq P, Gault N, Blandeau M, Kerdraon T, Berkal M, ElHelou A, et al. Long-term consequences of recurrent sports concussion. Acta neurochirurgica. 2016;158(2):289-300.
24. Hume PA, Theadom A, Lewis GN, Quarrie KL, Brown SR, Hill R, et al. A Comparison of Cognitive Function in Former Rugby Union Players Compared with Former Non-Contact-Sport Players and the Impact of Concussion History. Sports Med. 2016.
25. McMillan TM, McSkimming P, Wainman-Lefley J, Maclean LM, Hay J, McConnachie A, et al. Long-term health outcomes after exposure to repeated concussion in elite level: rugby union players. J Neurol Neurosurg Psychiatry. 2016.
26. Dubois B, Hampel H, Feldman HH, Scheltens P, Aisen P, Andrieu S, et al. Preclinical Alzheimer's disease: Definition, natural history, and diagnostic criteria. Alzheimers Dement. 2016;12(3):292-323.
27. Teasdale G, Jennett B. Assessment of coma and impaired consciousness. A practical scale. Lancet. 1974;2(7872):81-4.
28. Levin HS, Diaz-Arrastia RR. Diagnosis, prognosis, and clinical management of mild traumatic brain injury. Lancet Neurol. 2015;14(5):506-17.
29. Jordan BD. The clinical spectrum of sport-related traumatic brain injury. Nat Rev Neurol. 2013;9(4):222-30.
30. McCrory P, Meeuwisse WH, Aubry M, Cantu B, Dvorak J, Echemendia RJ, et al. Consensus statement on concussion in sport: the 4th International Conference on Concussion in Sport held in Zurich, November 2012. J Am Coll Surg. 2013;216(5):e55-71.
31. Giza CC, Kutcher JS, Ashwal S, Barth J, Getchius TS, Gioia GA, et al. Summary of evidence-based guideline update: evaluation and management of concussion in sports: report of the Guideline Development Subcommittee of the American Academy of Neurology. Neurology. 2013;80(24):2250-7.
32. Sharp DJ, Jenkins PO. Concussion is confusing us all. Pract Neurol. 2015;15(3):172-86.
33. Malec JF, Brown AW, Leibson CL, Flaada JT, Mandrekar JN, Diehl NN, et al. The mayo classification system for traumatic brain injury severity. J Neurotrauma. 2007;24(9):1417-24.
34. Jordan BD. Chronic traumatic encephalopathy and other long-term sequelae. Continuum (Minneap Minn). 2014;20(6 Sports Neurology):1588-604.
35. Martland H. Punch drunk. JAMA. 1928;91:1103-7.
36. Hof PR, Bouras C, Buee L, Delacourte A, Perl DP, Morrison JH. Differential distribution of neurofibrillary tangles in the cerebral cortex of dementia pugilistica and Alzheimer's disease cases. Acta Neuropathol. 1992;85(1):23-30.
37. Gavett BE, Stern RA, Cantu RC, Nowinski CJ, McKee AC. Mild traumatic brain injury: a risk factor for neurodegeneration. Alzheimers Res Ther. 2010;2(3):18.
38. Omalu BI, Hamilton RL, Kamboh MI, DeKosky ST, Bailes J. Chronic traumatic encephalopathy (CTE) in a National Football League Player: Case report and emerging medicolegal practice questions. J Forensic Nurs. 2010;6(1):40-6.
39. Johnson VE, Stewart W, Graham DI, Stewart JE, Praestgaard AH, Smith DH. A neprilysin polymorphism and amyloid-beta plaques after traumatic brain injury. J Neurotrauma. 2009;26(8):1197-202.
40. Kilbourne M, Kuehn R, Tosun C, Caridi J, Keledjian K, Bochicchio G, et al. Novel model of frontal impact closed head injury in the rat. J Neurotrauma. 2009;26(12):2233-43.

For peer review only - http://bmjopen.bmj.com/site/about/guidelines.xhtml

41. Kirchhoff C, Leidel BA, Kirchhoff S, Braunstein V, Bogner V, Kreimeier U, et al. Analysis of N-terminal pro-B-type natriuretic peptide and cardiac index in multiple injured patients: a prospective cohort study. Crit Care. 2008;12(5):R118.
42. Papa L, Ramia MM, Edwards D, Johnson BD, Slobounov SM. Systematic review of clinical studies examining biomarkers of brain injury in athletes after sports-related concussion. J Neurotrauma. 2015;32(10):661-73.
43. Zetterberg H, Blennow K. Fluid biomarkers for mild traumatic brain injury and related conditions. Nat Rev Neurol. 2016;12(10):563-74.
44. Shahim P, Tegner Y, Gustafsson B, Gren M, Arlig J, Olsson M, et al. Neurochemical Aftermath of Repetitive Mild Traumatic Brain Injury. JAMA Neurol. 2016;73(11):1308-15.
45. Shahim P, Zetterberg H, Blennow K. Neurofilament Protein and Antineurofilament Antibodies Following Traumatic Brain Injury-Reply. JAMA Neurol. 2017;74(3):363-4.
46. Shahim P, Zetterberg H, Tegner Y, Blennow K. Serum neurofilament light as a biomarker for mild traumatic brain injury in contact sports. Neurology. 2017;88(19):1788-94.
47. Julien JP, Mushynski WE. Neurofilaments in health and disease. Prog Nucleic Acid Res Mol Biol. 1998;61:1-23.
48. Perrone Capano C, Pernas-Alonso R, di Porzio U. Neurofilament homeostasis and motoneurone degeneration. Bioessays. 2001;23(1):24-33.
49. Lu CH, Allen K, Oei F, Leoni E, Kuhle J, Tree T, et al. Systemic inflammatory response and neuromuscular involvement in amyotrophic lateral sclerosis. Neurol Neuroimmunol Neuroinflamm. 2016;3(4):e244.
50. Posmantur R, Hayes RL, Dixon CE, Taft WC. Neurofilament 68 and neurofilament 200 protein levels decrease after traumatic brain injury. J Neurotrauma. 1994;11(5):533-45.
51. Oliver J, Jones M, Kirk M, Gable D, Repshas J, Johnson T, et al. Serum Neurofilament Light in American Football Athletes Over the Course of a Season. J Neurotrauma. 2015.
52. Gatson JW, Barillas J, Hynan LS, Diaz-Arrastia R, Wolf SE, Minei JP. Detection of neurofilament-H in serum as a diagnostic tool to predict injury severity in patients who have suffered mild traumatic brain injury. J Neurosurg. 2014;121(5):1232-8.
53. Shahim P, Tegner Y, Wilson DH, Randall J, Skillback T, Pazooki D, et al. Blood biomarkers for brain injury in concussed professional ice hockey players. JAMA Neurol. 2014;71(6):684-92.
54. Shahim P, Tegner Y, Marklund N, Hoglund K, Portelius E, Brody DL, et al. Astroglial activation and altered amyloid metabolism in human repetitive concussion. Neurology. 2017;88(15):1400-7.
55. Shahim P, Gren M, Liman V, Andreasson U, Norgren N, Tegner Y, et al. Serum neurofilament light protein predicts clinical outcome in traumatic brain injury. Sci Rep. 2016;6:36791.
56. Morris M, Maeda S, Vossel K, Mucke L. The many faces of tau. Neuron. 2011;70(3):410-26.
57. Naj AC, Schellenberg GD, Alzheimer's Disease Genetics C. Genomic variants, genes, and pathways of Alzheimer's disease: An overview. Am J Med Genet B Neuropsychiatr Genet. 2017;174(1):5-26.
58. Redensek S, Trost M, Dolzan V. Genetic Determinants of Parkinson's Disease: Can They Help to Stratify the Patients Based on the Underlying Molecular Defect? Front Aging Neurosci. 2017;9:20.
59. Al-Chalabi A, van den Berg LH, Veldink J. Gene discovery in amyotrophic lateral sclerosis: implications for clinical management. Nat Rev Neurol. 2017;13(2):96-104.
60. Fuller GW, Kemp SP, Decq P. The International Rugby Board (IRB) Pitch Side Concussion Assessment trial: a pilot test accuracy study. British journal of sports medicine. 2015;49(8):529-35.
61. Hinton-Bayre AD, Geffen G, Friis P. Presentation and mechanisms of concussion in professional Rugby League Football. J Sci Med Sport. 2004;7(3):400-4.
62. Delaney JS, Al-Kashmiri A, Correa JA. Mechanisms of injury for concussions in university football, ice hockey, and soccer. Clin J Sport Med. 2014;24(3):233-7.

For peer review only - http://bmjopen.bmj.com/site/about/guidelines.xhtml

63. Wadsworth M, Kuh D, Richards M, Hardy R. Cohort Profile: The 1946 National Birth Cohort (MRC National Survey of Health and Development). Int J Epidemiol. 2006;35(1):49-54.
64. Nofuji Y, Shinkai S, Taniguchi Y, Amano H, Nishi M, Murayama H, et al. Associations of Walking Speed, Grip Strength, and Standing Balance With Total and Cause-Specific Mortality in a General Population of Japanese Elders. J Am Med Dir Assoc. 2016;17(2):184 e1-7.
65. Cooper R, Kuh D, Hardy R. Objectively measured physical capability levels and mortality: systematic review and meta-analysis. BMJ. 2010;341:c4467.
66. Bohannon RW. Comfortable and maximum walking speed of adults aged 20-79 years: reference values and determinants. Age and ageing. 1997;26(1):15-9.
67. Schoon Y, Bongers K, Van Kempen J, Melis R, Olde Rikkert M. Gait speed as a test for monitoring frailty in community-dwelling older people has the highest diagnostic value compared to step length and chair rise time. Eur J Phys Rehabil Med. 2014;50(6):693-701.
68. Guralnik JM, Simonsick EM, Ferrucci L, Glynn RJ, Berkman LF, Blazer DG, et al. A short physical performance battery assessing lower extremity function: association with self-reported disability and prediction of mortality and nursing home admission. J Gerontol. 1994;49(2):M85-94.
69. Kuh D, Karunananthan S, Bergman H, Cooper R. A life-course approach to healthy ageing: maintaining physical capability. Proc Nutr Soc. 2014;73(2):237-48.
70. Roberts HC, Denison HJ, Martin HJ, Patel HP, Syddall H, Cooper C, et al. A review of the measurement of grip strength in clinical and epidemiological studies: towards a standardised approach. Age and ageing. 2011;40(4):423-9.
71. Jones CJ, Rikli RE, Beam WC. A 30-s chair-stand test as a measure of lower body strength in community-residing older adults. Res Q Exerc Sport. 1999;70(2):113-9.
72. Nilsdotter A, Bremander A. Measures of hip function and symptoms: Harris Hip Score (HHS), Hip Disability and Osteoarthritis Outcome Score (HOOS), Oxford Hip Score (OHS), Lequesne Index of Severity for Osteoarthritis of the Hip (LISOH), and American Academy of Orthopedic Surgeons (AAOS) Hip and Knee Questionnaire. Arthritis Care Res (Hoboken). 2011;63 Suppl 11:S200-7.
73. Collins NJ, Misra D, Felson DT, Crossley KM, Roos EM. Measures of knee function: International Knee Documentation Committee (IKDC) Subjective Knee Evaluation Form, Knee Injury and Osteoarthritis Outcome Score (KOOS), Knee Injury and Osteoarthritis Outcome Score Physical Function Short Form (KOOS-PS), Knee Outcome Survey Activities of Daily Living Scale (KOS-ADL), Lysholm Knee Scoring Scale, Oxford Knee Score (OKS), Western Ontario and McMaster Universities Osteoarthritis Index (WOMAC), Activity Rating Scale (ARS), and Tegner Activity Score (TAS). Arthritis Care Res (Hoboken). 2011;63 Suppl 11:S208-28.
74. Hamilton CB, Maly MR, Giffin JR, Clark JM, Speechley M, Petrella RJ, et al. Validation of the Questionnaire to Identify Knee Symptoms (QuIKS) using Rasch analysis. Health Qual Life Outcomes. 2015;13:157.
75. Folstein MF, Folstein SE, McHugh PR. "Mini-mental state". A practical method for grading the cognitive state of patients for the clinician. J Psychiatr Res. 1975;12(3):189-98.
76. Wechsler D. Wechsler Memory Scale - Revised Manual. Sant Antonio, TX: Psychological Corporation; 1987.
77. Papp KV, Amariglio RE, Dekhtyar M, Roy K, Wigman S, Bamfo R, et al. Development of a psychometrically equivalent short form of the Face-Name Associative Memory Exam for use along the early Alzheimer's disease trajectory. Clin Neuropsychol. 2014;28(5):771-85.
78. Rentz DM, Amariglio RE, Becker JA, Frey M, Olson LE, Frishe K, et al. Face-name associative memory performance is related to amyloid burden in normal elderly. Neuropsychologia. 2011;49(9):2776-83.
79. Aron AR, Monsell S, Sahakian BJ, Robbins TW. A componential analysis of task-switching deficits associated with lesions of left and right frontal cortex. Brain. 2004;127(Pt 7):1561-73.

For peer review only - http://bmjopen.bmj.com/site/about/guidelines.xhtml

80. Shallice T, Stuss DT, Picton TW, Alexander MP, Gillingham S. Multiple effects of prefrontal lesions on task-switching. Front Hum Neurosci. 2007;1:2.
81. Wechsler D. Wechsler Adult Intelligence Scale Sant Antonio, TX: Psychological Corporation; 1981.
82. Pertzov Y, Miller TD, Gorgoraptis N, Caine D, Schott JM, Butler C, et al. Binding deficits in memory following medial temporal lobe damage in patients with voltage-gated potassium channel complex antibody-associated limbic encephalitis. Brain. 2013;136(Pt 8):2474-85.
83. Parra MA, Abrahams S, Logie RH, Mendez LG, Lopera F, Della Sala S. Visual short-term memory binding deficits in familial Alzheimer's disease. Brain. 2010;133(9):2702-13.
84. Nelson HE, Willison J. The National Adult Reading Test (NART).
85. Say MJ, Jones R, Scahill RI, Dumas EM, Coleman A, Santos RC, et al. Visuomotor integration deficits precede clinical onset in Huntington's disease. Neuropsychologia. 2011;49(2):264-70.
86. Wechsler D. The Wechsler abbreviated scale of intelligence. San Antonio, TX: The Psychological Corporation; 1999.
87. Forster S, Lavie N. Entirely irrelevant distractors can capture and captivate attention. Psychon Bull Rev. 2011;18(6):1064-70.
88. Bremander AB, Dahl LL, Roos EM. Validity and reliability of functional performance tests in meniscectomized patients with or without knee osteoarthritis. Scand J Med Sci Sports. 2007;17(2):120-7.
89. Giovannoni G, van Schalkwyk J, Fritz VU, Lees AJ. Bradykinesia akinesia inco-ordination test (BRAIN TEST): an objective computerised assessment of upper limb motor function. J Neurol Neurosurg Psychiatry. 1999;67(5):624-9.
90. Doty RL, Shaman P, Dann M. Development of the University of Pennsylvania Smell Identification Test: a standardized microencapsulated test of olfactory function. Physiol Behav. 1984;32(3):489-502.
91. Doty RL, Frye RE, Agrawal U. Internal consistency reliability of the fractionated and whole University of Pennsylvania Smell Identification Test. Percept Psychophys. 1989;45(5):381-4.
92. Stiasny-Kolster K, Mayer G, Schafer S, Moller JC, Heinzel-Gutenbrunner M, Oertel WH. The REM sleep behavior disorder screening questionnaire--a new diagnostic instrument. Mov Disord. 2007;22(16):2386-93.
93. Gaiottino J, Norgren N, Dobson R, Topping J, Nissim A, Malaspina A, et al. Increased neurofilament light chain blood levels in neurodegenerative neurological diseases. PLoS One. 2013;8(9):e75091.
94. Kuhle J, Barro C, Andreasson U, Derfuss T, Lindberg R, Sandelius A, et al. Comparison of three analytical platforms for quantification of the neurofilament light chain in blood samples: ELISA, electrochemiluminescence immunoassay and Simoa. Clin Chem Lab Med. 2016;54(10):1655-61.
95. Rohrer JD, Woollacott IO, Dick KM, Brotherhood E, Gordon E, Fellows A, et al. Serum neurofilament light chain protein is a measure of disease intensity in frontotemporal dementia. Neurology. 2016;87(13):1329-36.
96. Pearce N. Effect measures in prevalence studies. Environ Health Perspect. 2004;112(10):1047-50.
97. Kaukiainen A, Hyvarinen HK, Akila R, Sainio M. Symptoms of chronic solvent encephalopathy: Euroquest questionnaire study. Neurotoxicology. 2009;30(6):1187-94.

For peer review only

For peer review only - http://bmjopen.bmj.com/site/about/guidelines.xhtml

Table 1: Glossary of definitions, adapted from (34)

**Chronic traumatic brain injury (TBI)**	A spectrum of disorders associated with long-term consequences of single or repetitive TBI
**Chronic traumatic encephalopathy (CTE)^**	Prototypic chronic TBI, long-term neurologic consequences of repetitive mild TBI
**Dementia pugilistica**	A subtype of CTE that is typically reserved for cases of severe end-stage dementia secondary to a long boxing career
**Chronic post-concussion syndrome^**	A condition in those athletes in whom post-concussion symptoms do not appear to resolve
**Chronic neurocognitive impairment^**	A rather diverse classification of chronic neurocognitive signs and symptoms secondary to head impact exposures and recurrent concussions that is theoretically distinctive from CTE
**Post-traumatic dementia***	Cases in which the athlete meets clinical criteria for dementia secondary to a single moderate to severe TBI
**Post-traumatic cognitive impairment***	Individuals who sustain long-term neurocognitive deficits from a single moderate to severe brain injury and do not meet clinical criteria for dementia, but instead mild cognitive impairment
**Post-traumatic parkinsonism**	A parkinsonian-like syndrome secondary to a single moderate to severe or repetitive TBI, occurring solely or as a component of CTE

^ the most clinically pertinent examples of sports-related chronic TBI ; *consequence of a single brain injury

For peer review only - http://bmjopen.bmj.com/site/about/guidelines.xhtml

Figure 1: Framework for data collection in the BRAIN study by domain

	Questionnaire	Physical ability	Cognitive ability	Neurological examination	Intermediate neurological outcomes	Blood sample
**'Core' Module**	• Lifestyle and confounders questionnaire<br>• Concussion history questionnaire ≯	• Height<br>• Weight<br>• Grip strength<br>• Chair rise^<br>• Walking speed#<br>• Photograph of hands	• MMSE<br>• WMS-R logical memory<br>• FNAME-12A<br>• Task-set shifting/Response inhibition<br>• WAIS-R digit symbol<br>• Visual short-term memory binding<br>• NART	• Video-recording~	• BRAIN test	• DNA*<br>• Plasma*
**Additional Optional Module**	• Pain mannequin (hand)	• HOOS<br>• KOOS<br>• QuIKS	• Visuomotor integration<br>• Matrix reasoning (WASI)<br>• Irrelevant Distractor Paradigm	• UPDRS-II	• Smell test<br>• RBD questionnaire	

*not to be collected if the participant is seen at home; ^subject to suitable chair availability for the participants seen at home; ~including knee bending test; #subject to space availability of the participant is seen at home; ≯to be administered as last item in all cases (after all 'Core' and Additional Optional module tests)

review only

For peer review only - http://bmjopen.bmj.com/site/about/guidelines.xhtml

Authors' contributions: state how each author was involved in writing the protocol.

Author's contributions

Valentina Gallo – co-Principal Investigator. Drafted the protocol, collected comments, and act as corresponding author

Damien McElvenny – co-Principal Investigator. Contributed designing the protocol, reviewed intellectual content of this paper

Huw Morris – Co-investigator. Advised on cognitive testing, biomarkers, and clinical definitions, reviewed this paper for intellectual content

Sebastian Crutch – Co-investigator. Advised on cognitive testing, and clinical definitions, reviewed this paper for intellectual content

Simon Kemp – Co-investigator. Advised on recruitment strategy, patient and public involvement (PPI) and reviewed this paper for intellectual content

Catherine Hobbs – Research assistant of the BRAIN Study. Recruits and collects informations on the study participants

Donna Davoren - Project administrator of the BRAIN Study. Coordinates the logistics of the study

Nigel Arden – Co-investigator. Advised on recruitment strategy, patient and public involvement (PPI) and reviewed this paper for intellectual content

Andrea Malaspina – Co-investigator. Advised on biomarkers

Matt Cross - Co-investigator. Advised on recruitment strategy, patient and public involvement (PPI) and reviewed this paper for intellectual content

Madeline Davies - Co-investigator. Advised on recruitment strategy, patient and public involvement (PPI), and reviewed this paper for intellectual content

Henrik Zetterberg – Co-investigator. Advised on biomarkers and reviewed this paper for intellectual content

Nick Fox - Co-investigator. Reviewed this paper for intellectual content

Neil Pearce – co-Principal Investigator. Contributed designing the protocol, reviewed intellectual content of this paper

Funding statement:

This study is funded by a grant from the Drake Foundation (www.drakefoundation.org/) to the London School of Hygiene and Tropical Medicine, with subcontracts to Queen Mary University of London (QMUL) and the Institute of Medicine (IoM).

For peer review only - http://bmjopen.bmj.com/site/about/guidelines.xhtml

Competing interests statement

None

For peer review only

For peer review only - http://bmjopen.bmj.com/site/about/guidelines.xhtml

STROBE Statement—Checklist of items that should be included in reports of ***cross-sectional studies***

	Item No	Recommendation	
**Title and abstract**	1	(*a*) Indicate the study's design with a commonly used term in the title or the abstract	OK
		(*b*) Provide in the abstract an informative and balanced summary of what was done and what was found	OK
**Introduction**			
Background/rationale	2	Explain the scientific background and rationale for the investigation being reported	OK, pp. 3-9
Objectives	3	State specific objectives, including any prespecified hypotheses	OK, pp. 9-10
**Methods**			
Study design	4	Present key elements of study design early in the paper	OK, pp. 10-11
Setting	5	Describe the setting, locations, and relevant dates, including periods of recruitment, exposure, follow-up, and data collection	OK, p. 10 as part of study design
Participants	6	(*a*) Give the eligibility criteria, and the sources and methods of selection of participants	OK, p.9
Variables	7	Clearly define all outcomes, exposures, predictors, potential confounders, and effect modifiers. Give diagnostic criteria, if applicable	OK, pp.10-18
Data sources/ measurement	8*	For each variable of interest, give sources of data and details of methods of assessment (measurement). Describe comparability of assessment methods if there is more than one group	OK, pp.10-18
Bias	9	Describe any efforts to address potential sources of bias	
Study size	10	Explain how the study size was arrived at	OK, pp.19-20
Quantitative variables	11	Explain how quantitative variables were handled in the analyses. If applicable, describe which groupings were chosen and why	OK, pp18-19
Statistical methods	12	(*a*) Describe all statistical methods, including those used to control for confounding	pp.18-19
		(*b*) Describe any methods used to examine subgroups and interactions	pp.18-19
		(*c*) Explain how missing data were addressed	TBD
		(*d*) If applicable, describe analytical methods taking account of sampling strategy	NA
		(*e*) Describe any sensitivity analyses	TBD
**Results**			
Participants	13*	(a) Report numbers of individuals at each stage of study—eg numbers potentially eligible, examined for eligibility, confirmed eligible, included in the study, completing follow-up, and analysed	NA
		(b) Give reasons for non-participation at each stage	NA
		(c) Consider use of a flow diagram	NA
Descriptive data	14*	(a) Give characteristics of study participants (eg demographic, clinical, social) and information on exposures and potential confounders	NA
		(b) Indicate number of participants with missing data for each variable of interest	NA
Outcome data	15*	Report numbers of outcome events or summary measures	NA

For peer review only

Main results	16	(*a*) Give unadjusted estimates and, if applicable, confounder-adjusted estimates and their precision (eg, 95% confidence interval). Make clear which confounders were adjusted for and why they were included	NA
		(*b*) Report category boundaries when continuous variables were categorized	NA
		(*c*) If relevant, consider translating estimates of relative risk into absolute risk for a meaningful time period	NA
Other analyses	17	Report other analyses done—eg analyses of subgroups and interactions, and sensitivity analyses	NA
**Discussion**			
Key results	18	Summarise key results with reference to study objectives	NA
Limitations	19	Discuss limitations of the study, taking into account sources of potential bias or imprecision. Discuss both direction and magnitude of any potential bias	pp.19-20
Interpretation	20	Give a cautious overall interpretation of results considering objectives, limitations, multiplicity of analyses, results from similar studies, and other relevant evidence	NA
Generalisability	21	Discuss the generalisability (external validity) of the study results	P. 21
**Other information**			
Funding	22	Give the source of funding and the role of the funders for the present study and, if applicable, for the original study on which the present article is based	P.29

*Give information separately for exposed and unexposed groups.

Note: An Explanation and Elaboration article discusses each checklist item and gives methodological background and published examples of transparent reporting. The STROBE checklist is best used in conjunction with this article (freely available on the Web sites of PLoS Medicine at http://www.plosmedicine.org/, Annals of Internal Medicine at http://www.annals.org/, and Epidemiology at http://www.epidem.com/). Information on the STROBE Initiative is available at www.strobe-statement.org.

For peer review only

For peer review only - http://bmjopen.bmj.com/site/about/guidelines.xhtml

BMJ Open

BRain health and healthy AgeINg in retired Rugby union players, the BRAIN study – study protocol

Journal:	*BMJ Open*
Manuscript ID	bmjopen-2017-017990.R1
Article Type:	Protocol
Date Submitted by the Author:	06-Oct-2017
Complete List of Authors:	Gallo, Valentina; Queen Mary, University of London, Centre for Primary Care and Public Health<br>McElvenny, Damien; Institute of Occupational Medicine,<br>Hobbs, Catherine; London School of Hygiene and Tropical Medicine Faculty of Epidemiology and Population Health<br>Davoren, Donna; London School of Hygiene and Tropical Medicine Faculty of Epidemiology and Population Health<br>Morris, Huw ; UCL<br>Crutch, Sebastian; University College London Institute of Neurology<br>Zetterberg, Henrik; Institute of Neuroscience and Physiology, Sahlgrenska Academy, University of Gothenburg., Department of Psychiatry and Neurochemistry<br>Fox, Nick; UCL, Dementia Research Centre<br>Kemp, Simon<br>Cross, Matthew; Rugby Football Union<br>Arden, Nigel; Nuffield Department of Orthopaedics, ;<br>Davies, Madeleine; University of Oxford, Nuffield Department of Orthopaedics, Rheumatology and Musculoskeletal Science<br>Malaspina, Andrea; Queen Mary University of London<br>Pearce, Neil; London School of Hygiene and Tropical Medicine
**Primary Subject Heading**:	Sports and exercise medicine
Secondary Subject Heading:	Epidemiology, Neurology
Keywords:	Rugby, concussion, Rugby Union, cross-sectional study, cognitive function, healthy ageing

SCHOLARONE™ Manuscripts

BRain health and healthy AgeINg in retired Rugby union players, the BRAIN study – study protocol

Valentina Gallo[1,2,3], Damien McElvenny[4], Catherine Hobbs[3], Donna Davoren[3], Huw Morris[5], Sebastian Crutch[6], Henrik Zetterberg[7,8,9,10], Nick C Fox[6], Simon Kemp[11], Matt Cross[11], Nigel Arden[12], Madeleine A.M. Davies[12], Andrea Malaspina[13], Neil Pearce[3]

1. Centre for Primary Care and Public Health, Queen Mary University of London, London, UK
2. School of Public Health, Imperial College London, London, UK
3. London School of Hygiene and Tropical Medicine, London, UK
4. Institute of Occupational Medicine, Edinburgh, UK
5. University College London, London, UK
6. Dementia Research Centre, UCL Institute of Neurology, University College London, London, UK
7. Department of molecular neuroscience, UCL Institute of Neurology, Queen Square, London
8. UK Dementia Research Institute, London, UK
9. Clinical Neurochemistry Laboratory, Sahlgrenska University Hospital, Mölndal, Sweden
10. Department of Psychiatry and Neurochemistry, Institute of Neuroscience and Physiology, the Sahlgrenska Academy at the University of Gothenburg, Mölndal, Sweden
11. Rugby Football Union (RFU), London, UK
12. Arthritis Research UK Centre for Sport, exercise and osteoarthritis, University of Oxford, Oxford, UK
13. Department of Neuroscience and Trauma, Blizard Institute, Queen Mary, University of London, London, UK

Please address all correspondence to:

Dr Valentina Gallo

Centre for Primary Care and Public Health
Queen Mary, University of London
Yvonne Carter Building
58, Turner Street
E1 2AB London (UK)
valentina.gallo@lshtm.ac.uk or v.gallo@qmul.ac.uk

For peer review only - http://bmjopen.bmj.com/site/about/guidelines.xhtml

Abstract

Introduction - Relatively little is known about the long-term health of former elite rugby players, or former sportspeople more generally. As well as the potential benefits of being former professional sportspersons, there may be potential health risks from exposures occurring during an individual's playing career, as well as following retirement. Each contact sport has vastly different playing dynamics, therefore exposing its players to different types of potential traumas. Current evidence suggests that these are not necessarily comparable in terms of pathophysiology, and their potential long-term adverse effects might also differ. There is currently limited but increasing evidence that poorer age-related and neurological health exists among former professional sportsmen exposed to repetitive concussions; however the evidence is limited on rugby union players, specifically.

Methods and analysis – We present the protocol for a cross-sectional study to assess the association between self-reported history of concussion during the playing career, and subsequent measures of healthy ageing and subtle neurological and cognitive impairment. We are recruiting a sample of approximately 200 retired rugby players (former Oxford and Cambridge University rugby players and members of the England Rugby International Club) aged 50 years or more, and collecting a number of general and neurological health-related outcome measures though validated assessments. Biomarkers of neurodegeneration (neurofilaments and tau) will be also be measured. Although the study is focusing on rugby union players specifically, the general study design and the methods for assessing neurological health are likely to be relevant to other studies of former professional sportspersons.

Ethics and dissemination – The study has been approved by the Ethical Committee of London School of Hygiene and Tropical Medicine (LSHTM) (reference: 11634 [illegible] 2). It is intended that results of this study will be published in peer reviewed medical journals, communicated to participants, the general public, and all relevant stakeholders.

Keywords:

Rugby, concussion, Rugby Union, cross-sectional study, cognitive function, healthy ageing, neurological health, sport

For peer review only - http://bmjopen.bmj.com/site/about/guidelines.xhtml

Introduction

The evidence relating to head trauma in sport and the subsequent risks of neurological disease have previously reviewed, and it has been established that sports involving repeated head trauma may have an increased risks of neurodegenerative disease in the long-term (1). Furthermore, there are now plausible mechanisms for these effects, and a recognition that these problems do not just occur in former boxers, but in a variety of sports involving repeated concussions (2), and possibly also in sports in which low-level head trauma is common (3). These neurodegenerative effects include potentially increased risks of impaired cognitive function and dementia (4-7), Parkinson's disease (PD) (8-13), and amyotrophic lateral sclerosis (ALS) (3, 14-19). The term *chronic traumatic encephalopathy* (CTE) was introduced as a clinical-pathological construct for the neurodegeneration associated with American football and wrestling (20) (see Table 1). Seminal work by Ann McKee (21) investigating CTE has led to important deliberations on the overlapping neurodegenerative lesions in this condition (22). However, each contact sport has vastly different playing dynamics therefore exposing its players to different types of potential traumas. Current evidence suggests that these are not necessarily comparable in terms of pathophysiology, and hence in terms of their potential long-term adverse effects on health. There is currently limited but increasing evidence that poorer general and neurological health exists among professional sportsmen exposed to repetitive concussions; however, there is little evidence from rugby union players (23-25).

Decq et al (23) investigated retired French-speaking high-level sportsmen, aged 45 to 65 years, who had played sports for at least 10 years. Mild cognitive disorder (measured as score at the modified Telephone Interview for Cognitive Status (TICS-m) $\leq$30) was lower in players of other sports (40.4%) than in former rugby players (56.6%) (p=0.005). However, after adjustment for smoking and higher education, no association was observed between TICS-m score and number of reported concussions (23). Furthermore, in New Zealand, 366 former players were tested on their engagement in sport, general health, sports injuries and concussion history, and demographic information. Cognitive functioning was assessed using the online CNS Vital Signs neuropsychological test battery. The elite rugby group performed worse on tests of complex attention, processing speed, executive functioning, and cognitive flexibility than the non-contact-sport group, and worse than the community rugby group on complex attention. Former players who recalled one or more concussions had worse scores on cognitive flexibility, executive functioning, and complex attention than players who did not recall experiencing a concussion (24). A recent Scottish study assessed 52 former Scottish international rugby players and 29 controls. Players performed worse on a test of verbal learning, and of fine co-ordination

For peer review only - http://bmjopen.bmj.com/site/about/guidelines.xhtml

of the dominant hand; however, no statistically significant differences were observed on other cognitive tests. Additionally, no significant association was found between the number of concussions and cognitive test performance (25).

Ultimately, establishing the extent of these potential issues will require long-term prospective studies involving the repeated measurement of various to head trauma exposures and repeated tests of neurological health in large numbers of current and former players. Furthermore, identifying the risks, if any, of severe neurological problems, such as PD and ALS, will require large numbers of participants and long periods of follow-up. However, a first step in this process is to conduct cross-sectional studies in former players to assess whether there is an association between the history of concussion during their rugby career, and subsequent measures of healthy ageing and subtle neurological and cognitive impairment. In particular, such cross-sectional studies can be conducted in a relatively short time and with ‘standard’ measures of cognitive function since impaired cognitive function is an important health outcome in itself, and may be a precursor of more serious long-term neurological effects (26). Furthermore, cross-sectional studies of this type can also be conducted in current players, and thus form the basis for prospective studies measuring changes in neurological health over time.

Definitions of trauma and concussion

Traumatic brain injury (TBI) is usually classified as mild, moderate, or severe, on the basis of the initial Glasgow Coma Scale (GCS) (27) recorded in the Emergency Departments including the duration of any loss of consciousness; and duration of post-traumatic amnesia (i.e., loss of memory of events after the injury) (28). Chronic TBI represents a spectrum of disorders associated with long-term consequences after single or repetitive TBI (29). Conversely, there remains no consensus on the definition of concussion. The 2012 Zurich Consensus Statement on Concussion in Sport proposed that concussion and mild TBI should be viewed as distinct entities (30). The group defined concussion as a “complex pathophysiological process affecting the brain”, and despite allowing for the presence of neuropathological damage, they postulated that concussive symptoms largely reflected a functional disturbance, typically resolving spontaneously with no imaging abnormality. In contrast, recent guidelines from the American Academy of Neurology for sports concussion in 2013, do not separate concussion from mild TBI, defining concussion as “a clinical syndrome of biomechanically induced alteration of brain function, typically affecting memory and orientation, which may involve loss of

For peer review only - http://bmjopen.bmj.com/site/about/guidelines.xhtml

consciousness" (31). A recent report (32) examines why having two different pathological entities might be unhelpful and suggests that the Mayo Clinic TBI Classification system should cover both definitions(33). A glossary of definitions used in this protocol is found in Table 1, adapted from Jordan (2014) (34).

For the purpose of this study, we adapted the NIH definition of concussion (35). Participants will be asked to report their previous concussions according with the following definition:

Concussion is defined as an alteration in brain function, caused by an external force. Symptoms include:

- *A decreased level / loss of consciousness*
- *Memory Loss (before or after the injury)*
- *Weakness*
- *Temporary Paralysis*
- *Loss of balance*
- *Change in vision (e.g. blurriness, double vision)*
- *Co-ordination difficulties*
- *Numbness*
- *Decreased sense of smell*
- *Difficulty understanding what others are saying*
- *Difficulty communicating with others*
- *Confusion, disorientation, or slowed thinking*

Please note, loss of consciousness is not required for a concussion to be diagnosed.

For peer review only

Impaired cognitive function and dementia in sportspersons

While the neurological and cognitive effects of acute traumatic brain injuries (TBI) have been extensively studied (4-7), the association between TBI and delayed sequelae have been less studied because of the variable latency period before overt neurologic dysfunction, and the difficulty of retrospectively recalling relevant events (recall bias), in particular in the presence of memory impairment. The neurocognitive effects of repetitive mild head injury in sport were initially observed in boxers, a syndrome that was distinct from the clinical and pathological sequelae of single-incident severe TBI (36) (see also Table 1). The clinical syndrome of dementia pugilistica (punch-drunk syndrome) is associated with prominent

For peer review only - http://bmjopen.bmj.com/site/about/guidelines.xhtml

tauopathy, with typical neurofibrillary tangles and neuropil threads, distributed in patches throughout the neocortex (37). Neuropathological case series have demonstrated primarily tau-related pathology in the brains of individuals who have suffered from a clinical syndrome encompassing dementia and movement disorders after repeated head trauma (38). Also, post-mortem examination of the brains of several professional American football players and wrestlers has revealed the pathological underpinnings of cognitive and neuropsychiatric decline seen in these men in later life (20). Although cognitive decline in professional American football players has been suggested for some years, the first autopsy report from such a player was published only recently (39). In all cases, cognitive decline began years after retirement from formal sport participation.

Cognitive function is an ideal outcome measure for this type of study, as being a continuous measure maximizes power to identify an association; and using neurodegenerative disease as an outcome measure would require a substantially larger sample size. Cognitive function, as measured with neurocognitive tests, is also an aspecific measure, which does not imply a clinical diagnosis, and it can be the prodrome manifestation for a number of different neurodegenerative diseases.

Biomarkers

Research in the field of TBI biomarkers has increased exponentially over the last 20 years (29, 40-42), with studies assessing biomarkers that could provide diagnostic and prognostic, as well as monitoring information. S100β, glial fibrillary acidic protein (GFAP), neuron-specific enolase (NSE), tau, neurofilament light protein (NF-L), amyloid beta, brain-derived neurotrophic factor (BDNF), serum creatinine kinase (CK), and heart-type fatty acid binding protein (hFABP), prolactin, cortisol, and albumin are all biomarkers more frequently studied in relation to sport-related concussion (43, 44). However, to date, no biomarker for the long-term effects of concussion has been identified, although recent studies on cerebrospinal fluid (CSF) from sportsmen with post-concussion syndrome suggest that a subset of these have biomarker signs of ongoing axonal injury and microglial activation (45-47).

Of the biomarkers measurable in serum or other body fluids, those more likely to be detectable for longer time periods after the concussion episode, are those related to the axonal injury and more in generally neuroinflammation, that is neurofilament and tau protein (44). Neurofilaments (NF) co-assemble from protein subunits to form NFs defined as light (light (NF-L), medium (NF-M), or heavy (NF-H) according to their relative molecular weights). NFs are one of the key structural elements of neurons,

For peer review only - http://bmjopen.bmj.com/site/about/guidelines.xhtml

providing mechanical stability and determining axonal diameter. NFs are of particular interest because as they are structural elements, they may be more susceptible to mechanical deformation under the condition of trauma. Abnormal NF aggregation may contribute to the delayed or progressive neuronal death and dysfunction taking place in neurodegenerative diseases associated with NF aggregation, such as PD, ALS, and Lewy body dementia (LBD) (48-50). Acute perturbations of NFs have been demonstrated in experimental models of TBI that produce cortical contusion in combination with selective hippocampal neuronal death (51). A study compared serum NF-L, a biological marker of head trauma, in American football athletes and non-contact sport athletes and examined changes over the course of a season. Results suggest that a season of collegiate American football is associated with elevations in serum NF-L, which is indicative of axonal injury, resultant of head impacts (52). NF-L has also been associated with head trauma severity detected by CT scan, immediately after the episode (53) and with concussive and subconcussive head impacts in boxing (47). Importantly, whilst plasma tau concentration increases and disappears rapidly (within hours to a few days) following concussion (54), NF-L has a prolonged increased levels lasting for many weeks (47, 55, 56).

Phosphorylation of tau is a normal event in healthy neurons, but hyperphosphorylation and aggregation into neurofibrillary tangles is a characteristic of Alzheimer's disease and CTE (57). Tau concentrations correlate with lesion size and outcome in severe TBI when measured in the ventricular CSF, while remaining unchanged in TBI and other forms of acute brain injury (44). Studies on mild TBI show increased CSF concentrations of both t□tau and NF□L, although the increase in CSF NF-L is greater than the increase in t-tau, suggesting that head impacts have a greater effect on long, large-calibre axons that extend subcortically than on short, nonmyelinated axons in the cortex (44). After concussion, serum tau concentrations were found to be increased, but this increase did not correlate with severity of trauma and lesion load as measured using CT scan signs (44).

Genetics

Few hypotheses on the potential role of genetics in modulating the possible association between concussion and cognitive decline have been formulated. Specific genes might be associated with an increased risk of concussion via worse attention or executive function, or more vulnerable brain anatomy (58), or via personality trait (59). Conversely, a number of genes are involved in modulating the risk of developing dementia and other neurodegenerative diseases, irrespective of concussion. Dementia risk is higher amongst carriers of the epsilon4 allele at the Apolipoprotein E (APOE) gene (60).

For peer review only - http://bmjopen.bmj.com/site/about/guidelines.xhtml

A number of Genome Wide Association Studies (GWAS) has been performed to date, enabling the identification of 24 loci as risk factors for Parkinson's disease. These loci take part in numerous cellular processes that may contribute to PD pathology, and protein aggregation (e.g. *SNCA* coding for α-synuclein; and *MAPT* encoding a microtubule-associated protein tau), protein, and membrane trafficking (e.g. *LRRK2* (leucine-rich repeat kinase 2) gene), lysosomal autophagy (e.g. GBA (beta acid glucosidase)), immune response (e.g. *BST1* (bone marrow stromal cell antigen-1)), synaptic function, endocytosis, inflammation, and metabolic pathways are among the most important (61). The identified single nucleotide polymorphisms are usually located in the non-coding regions and their functionality remains to be determined, although they presumably influence gene expression. Similarly, since the discovery of mutations in SOD1 gene, which account for ~2% of ALS cases, increasing efforts have been made to understand the genetic component of ALS risk. Specifically mutation in the chromosome 9 open reading frame 72 (C9orf72) have been associated with both ALS and Fronto-Temporal Dementia (FTD) (62).

Rationale of the BRAIN Study

Recently, World Rugby (formally known as the International Rugby Board), has developed a process to support team clinicians in the recognition, assessment, and subsequent management of elite adult players who have sustained a potential concussion. This process includes the development of a multimodal assessment: the Head Injury Assessment, formerly the Pitch Side Concussion Assessment (PSCA) tool (63).

However, there is little clear evidence, about the possible long-term effects of concussion in rugby union players, and it is not clear if findings from other sports are directly generalizable to rugby players. Many sports involve exposure to concussion or repetitive low-level head trauma, and it can be argued that each sport should be considered independently, due to the unique technical and physiological profile that a player develops over the course of a career (64, 65). Thus, to determine the potential risk of long-term adverse health effects of playing rugby specific studies of rugby players are needed.

The BRAIN Study builds upon a recent cross-sectional questionnaire-based study conducted by University of Oxford as part of the Arthritis Research UK sport, exercise and osteoarthritis centre, who have assessed the general and musculoskeletal health of former elite rugby players (66). Rugby players from the Oxford and Cambridge University Football Clubs (Oxbridge Blues), and members of the England Rugby Internationals Club (ERIC) – a membership organization of all current and former England players

For peer review only - http://bmjopen.bmj.com/site/about/guidelines.xhtml

- were recruited. Self-reported demographic factors, playing history (including head trauma), past medical history (including dementia, depression, and memory impairment), and perceived health were collected, in addition to detailed information on musculoskeletal health and pain.

A total of 319 participants have participated in this study to date, and 205 of those aged 50 years or older have agreed to be re-contacted for further studies. The 205 participants aged 50+ years who agreed to be re-contacted will be invited to take part to the BRAIN Study.

Aim and objectives

The overall aim of the BRAIN study is to investigate the possible associations between concussion in rugby and ageing, including physical and cognitive capabilities, as well intermediate neurological and musculoskeletal endpoints among former rugby players.

In order to achieve these aims the following objectives will be pursued:

1. To investigate the associations between self-reported concussion history and ageing, measured as physical and cognitive capabilities. This will be achieved by using the following outcome measures:
    a. Physical capability outcomes: grip strength, chair raise and walking speed
    b. Cognitive capability outcomes: memory, reasoning, speed of thinking and attention, and verbal and numerical skills.
2. To investigate the association between self-reported concussion history and intermediate neurological endpoints
    a. Neurological examination: a brief neurological examination will be video-recorded according to existing protocols and independently examined by two neurologists
    b. Intermediate neurological endpoints: information using tapping test (BRAIN), smell test, REM-behaviour disorder questionnaire will be collected in order to explore non motor symptoms of Parkinson's disease and related disorders
3. To investigate whether a history of concussion is associated with current tau protein and NF-L levels in blood (potential biomarkers of neurodegeneration), and how these biomarkers are in turn associated with the outcome measures (potential biomarkers of early detection of disease)
4. To assess if any other characteristic of rugby playing history, in addition to self-reported concussion (i.e. length of playing at elite level, position of play, numbers of game played, age

For peer review only - http://bmjopen.bmj.com/site/about/guidelines.xhtml

when started playing), or age at concussion is associated with any outcome measure (physical and cognitive capability and/or intermediate neurological outcomes)

5. Investigate the long-term musculoskeletal health outcomes of rugby players with particular emphasis on hip, knee, and hand osteoarthritis allowing changes over time (from the previous study to the current study) to be assessed

In addition, we will develop a multimedia database (data from tests and questionnaire plus video-recorded neurological examination plus biobank of blood samples) that will serve as baseline for further tests and further follow-up.

Study design

Participants will be invited to study clinics in London, Manchester, or Bath. A home visit can also be arranged by request. Participants will be invited to bring with them any medication they are taking regularly for an accurate recording of current medication usage.

All participants will be administered a ***Core Module*** interview including questions on lifestyle factors, potential confounders, and extensive information on the five domains of physical and cognitive ability, neurological examination, intermediate neurological outcomes and musculoskeletal health.

The lifestyle questionnaire will complement that already administered to participants of the Oxford-base cross-sectional study, and will include questions on potential confounders of the association between concussion and physical and cognitive capability, and intermediate neurological outcomes. Participants will also be asked to donate a blood sample (a normal blood sample will be collected by participants seen in one of the clinics, a dry spot sample plus saliva swab for DNA will be collected from participants seen at home). At the end of the Core Module, all participants will be also asked to undergo some additional tests (the ***Additional Optional Module***) providing they have sufficient time (Figure 1). At the end of the interview, all participants will be asked some final questions on their concussion history. This will ensure that the interviewer is blind to the participant's concussion history during the entire duration of the interview, and will also act as validation to confirm data previously collected. Participants who disclose information about their concussion history during the interview, will be noted in order to undertake a sensitivity analysis excluding them.

For peer review only - http://bmjopen.bmj.com/site/about/guidelines.xhtml

The ***Core Module*** includes: 1) a lifestyle questionnaire; 2) a set of tests covering essential information on physical (height, weight, grip strength, chair rise, walking speed and photo of the hands) and cognitive capability (Mini-Mental State Examination, Logical Memory, Digit-Symbol Substitution test, Matrix Reasoning, Task-set Shifting/Response Inhibition, Visuomotor Integration, 12-item Face-Name Associative Memory Exam, and the National Adult Reading Test); 3) a remote neurological clinical examination (video recorded); and 4) a test for subtle movement disorders, the BRAIN tapping test. This Core Module should take no longer of 1 hour and 45 minutes to be completed.

The ***Additional Optional Module*** includes: 1) a questionnaire investigating hand pain; 2) extra tests investigating the cognitive domain (Visual short-term memory binding, Irrelevant Distractor Paradigm); 2) the UPDRS part II scale to complete the neurological examination (not video-recorded); 3) additional tests for intermediate neurological outcomes (the smell test and the REM behaviour disorder questionnaire). This Additional Optional Module should take no longer than 1 hour and half to be completed (Figure 1). The full test/interview including core and additional module should not take more than 3 hours and 15 minutes to complete.

General Questionnaires

Questionnaires will be used to collect relevant information on:

Lifestyle and confounders– all participants will be asked a number of questions on their lifestyle in order to collect information on possible confounders for the main analysis (smoking, alcohol, coffee, drugs, past medical history, sleep quality).

Concussion history– at the end of the interview, participants will be re-asked some detailed information regarding their history of head trauma and concussion whilst playing.

Musculoskeletal hand pain – A questionnaire on hand pain to identify possible hand osteoarthritis will be utilized involving a hand mannequin (additional optional module).

Physical ability assessment

The methodology used in the 1946 birth cohort (67) will be leveraged to assess physical and cognitive capabilities in this study. This will enhance comparability of results and facilitate future collaborations. The most commonly used objective measures of physical capability for assessing healthy ageing, are

For peer review only - http://bmjopen.bmj.com/site/about/guidelines.xhtml

For peer review only

tests of grip strength (68, 69), walking speed (68, 70, 71), chair rises (72) and standing balance (68); these aim to assess physical functioning, including the capacity to undertake the physical tasks of daily living (73). There is robust evidence that higher scores on these measures are associated with lower rates of mortality, and there is more limited evidence of lower risks of morbidity, and of age-related patterns of change (73).

Height and weight – height and weight will be collected in order to calculate the body mass index (BMI) according to the formula BMI = weight (Kg) / height $(m)^2$.

Grip strength - JAMAR Hydraulic Hand Dynamometer is the most widely used instrument with established test-retest, inter-rater and intra-rater reliability (74).

Chair rise - The 30-Second Chair Stand Test will be used to assess lower body strength (75). This test provides a reasonably reliable and valid indicator of lower body strength in generally active, community-dwelling older adults. Test-retest intra-class correlations of 0.84 for men and 0.92 for women, utilizing one-way analysis of variance procedures appropriate for a single trial, together with a non-significant change in scores from Day 1 testing to Day 2, indicate that the 30-s chair stand has good stability reliability (75). Moreover, a moderately high association between chair-stand performance and maximum weight-adjusted leg-press performance for both men and women (r = 0.78 and 0.71, respectively) supports the criterion-related validity of the chair stand as a measure of lower body strength (75). As expected, chair-stand performance decreased significantly across age groups in decades – from the 60s to the 70s to the 80s ($p < 0.01$) and was highly statistically significantly lower for low-active participants than for high-active participants ($p < 0.0001$) (75).

Walking speed – To assess normal comfortable walking speed and maximum walking speed, a Timed 10-Meter Walk Test will be used. This test has been validated in 230 healthy volunteers (70). Mean comfortable gait speed ranged from 127.2 cm/s for women in their seventies to 146.2 cm/s for men in their forties. Mean maximum gait speed ranged from 174.9 cm/s for women in their seventies to 253-3 cm/s for men in their twenties. Both gait speed measures were reliable (correlation coefficients > 0.903) and correlated significantly with age (r>-0.210), height (r>0.220) and the strengths of four measured lower extremity muscle actions (r = 0.190-0.500). These normative values give clinicians a reference against which patient performance can be compared in a variety of settings (70).

For peer review only - http://bmjopen.bmj.com/site/about/guidelines.xhtml

A photograph of the hands will be taken at the end of this section to assess inflammation, finger-nodes, and other rheumatological and osteoarthritis-related changes at the hand.

HOOS – The Hip Disability and Osteoarthritis Outcome Score (HOOS) measures patient's opinions about their hip and associated problems. It examines pain, symptoms, function in activities of daily living (ADL) and function in sport and recreation. It has been used in subjects with hip disability with or without hip osteoarthritis (OA) (76) (Additional optional module).

KOOS – The Knee Injury and Osteoarthritis Outcome Score (KOOS) examines pain frequency and severity during functional activities, and symptoms such as the severity of knee stiffness, the presence of swelling, grinding or clicking, difficulty in ADL, with sport and recreation, and knee-related quality of life. It is intended for use in young and middle-aged populations with post-traumatic OA, in addition to those with injuries who may go on to develop secondary OA (77) (Additional optional module).

QuIKS – The Questionnaire to Identify Knee Symptoms (QuIKS) is used for early osteoarthritis, and understanding symptomology and potential adaptation to activity before osteoarthritis has been clinically diagnosed. It has been developed in adults aged 40-65 with evidence of ongoing knee problems and recommended for use in studies exploring early osteoarthritis (78) (Additional optional module).

Cognitive ability assessment

The methodology used in the 1946 birth cohort will also be followed for the assessment of cognitive capability (67). Tests and questionnaires will be described according their belonging to the Core Module or the Additional Optional Module, and by domain (Figure 1).

Mini-Mental State Examination (MMSE (79)) – The MMSE is a widely used 30-point screening tool for cognitive impairment within clinical practice, assessing multiple cognitive domains including orientation to time and place; registration; attention +/- calculation; recall; language; repetition; reading; writing; visuospatial function; and executive function and praxis.

Logical Memory from the ***Wechsler Memory Scale-Revised*** (WMS-R (80)) - The Logical Memory test assesses free recall of a short story. The participant is asked to recall the story immediately and after a 20minute delay.

12-item Face-Name Associative Memory Exam (FNAME-12A) – The FNAME-12A is a modified version of the 16-item Face-Name Associative Memory Exam (FNAME-16). The FNAME-12A has fewer stimuli and

For peer review only - http://bmjopen.bmj.com/site/about/guidelines.xhtml

additional learning trials which have been found to be well tolerated by those with Mild Cognitive Impairment, whilst remaining challenging in cognitively normal older adults (81). The FNAME-12A has demonstrated psychometric equivalence with the FNAME-16, which has been shown to be related to beta-amyloid burden in cognitively normal elderly people (82). The FNAME-12A requires the participant to learn 12 face-name and face-occupation pairs. Participants are given two exposures to all 12 face-name/occupation pairs. After each exposure, and following a 5-minute delay, participants are asked for the name and occupation associated with each face. After a 30-minute delay they are shown 3 faces and asked to identify the face that they recognise and give the name and occupation. Given multiple options to choose from, they are then asked to select the name and/or occupation associated with the face.

Task-set Shifting/Response Inhibition (83, 84) – The task-set shifting and response inhibition task examines the relationship between executive tasks of task-switching/preparation time. In the arrow only condition, participants are shown the cue 'arrow'. Following a short delay they must respond to the direction of the arrow ('right' or 'left'). In the word only condition, participants are shown the cue 'word'. Following a short delay they must respond to the direction of the word ('right' or 'left'). There is a switching condition in which the participant is shown the cue 'arrow' or 'word'. Following a delay, both a combined arrow and word stimulus appears. The stimulus is either congruent (left arrow and left word), or incongruent (left arrow and right word). Trials in the switching task are categorised into switch and non-switch. In a non-switch trial the cue is the same as for the immediately preceding trial. In a switch trial the cue differs from the immediately preceding trial.

Digit-Symbol Substitution Test, from the ***Wechsler Adult Intelligence Scale-Revised*** (WAIS-R (85)) - The Digit-Symbol Substitution test explores attention and psychomotor speed. Participants are given a code table displaying digits (from 1 to 9); each digit is paired with a symbol. The participant is required to complete in blank squares with the corresponding symbol for each digit as shown in the code table. They are given 90 seconds to fill in as many squares as possible.

Visual Short-term Memory Binding (86, 87) – This test requires the participant to view one or three fractal objects, presented simultaneously in random locations on the screen. The participant is asked to remember both the objects and their location. After a delay of 1 or 4 seconds they have to make a forced choice between one of the displayed fractals (the target) and a 'dummy' fractal. Participants are required to touch the object they think has been previously presented and 'drag' it on the touch screen to its remembered, original location. The binding of such featured information has been shown to be vulnerable in asymptomatic FAD mutation carriers (87).

For peer review only - http://bmjopen.bmj.com/site/about/guidelines.xhtml

National Adult Reading Test (NART) – The NART was specially designed to provide a means of estimating the premorbid intelligence levels of adults suspected of suffering from intellectual deterioration (88). The NART comprises a list of 50 words printed in order of increasing difficulty. The words are relatively short in order to avoid the possible adverse effects of stimulus complexity on the reading of dementing subjects, and they are all 'irregular' with respect to the common rules of pronunciation in order to minimise the possibility of reading by phonemic decoding rather than word recognition (88).

Visuo-motor Integration (89) – This is a circle tracing task which includes both direct and indirect visual feedback conditions. Continuous performance measures are provided including accuracy, speed, and speed of error detection and correction. The test has revealed changes in speed and accuracy in Huntington Disease mutation carriers more than 10 years before expected age-of-onset (Additional optional module).

Matrix Reasoning from the ***Wechsler Abbreviated Scale of Intelligence*** (WASI (90)) – The Matrix Reasoning test assesses nonverbal reasoning. The participant is shown a matrix of geometric shapes with a section missing. They are required to select the option that completes the matrix (Additional optional module).

Irrelevant Distractor Paradigm (91) – Participants are given a computerised letter-search task and are required to make a rapid decision as to whether the target letter 'X' or 'N' has appeared in the search display (in either low or high load conditions). On some of the trials, a task-irrelevant distractor (a cartoon character) appears on the outside of the search display. The task evaluates the extent to which attention is captured and captivated by the distractor (Additional optional module).

Neurological clinical assessment

The neurological assessment of participants is needed for detecting any subtle neurological sign, and to establish a baseline for the absence of one or more signs. A video-recorded standard neurological examination will be included as part of the Core Module. An additional test of self-reported impairment, mainly due to movement disorders, will be administered as part of the Additional Optional Module (Figure 1).

A standard video recording of each participant accessing the study at one of the clinics will be performed and evaluated by a clinical neurologist. The assessment includes examination of strength,

For peer review only

For peer review only - http://bmjopen.bmj.com/site/about/guidelines.xhtml

coordination, balance, ocular movements, cranial nerves, gait, and repeated movements. Theexamination includes also a test of knee functionality (92).

Information on potential movement disorders will be collected using the unified Parkinson's Disease Rating Scale (PDDRS) part II. This includes self-reported ratings on several motor domains, including speech, saliva and drooling, chewing and swallowing, eating, dressing, hygiene, handwriting, hobbies, turning in bed, tremor, standing up, walking and balance, and freezing (Additional optional module).

Intermediate neurological endpoints

In addition to neurological signs and symptoms, some intermediate outcomes have been recognised as part of the complex clinical picture of movement disorders, manifesting before the onset of the movement impairment itself. These will be investigated with three tests described below (Figure 1).

BRAIN tap test –This is a simple and rapid computerised keyboard test, based on the alternating finger tapping test, which has been developed to quantify upper limb motor function (93). The test generates several variables: 1) kinesia score: the number of keystrokes in 60 seconds; 2) akinesia time: cumulative time that keys are depressed; 3) dysmetria score: a weighted index calculated using the number of incorrectly hit keys corrected for speed; 4) in-coordination score: a measure of rhythmicity which corresponds to the variance of the time interval between keystrokes (93). The BRAIN test provides a simple, rapid, and objective assessment of upper limb motor function. It assesses speed, accuracy, and rhythmicity of upper limb movements regardless of their physiological basis. The results of the test correlate well with clinical rating scales in Parkinson's disease and cerebellar dysfunction. The BRAIN tap test is useful in clinical studies (93). The BRAIN tap test is available online at https://www.braintaptest.com/en_GB and VG has obtained access for generating tokens for administering the test to participants (A Noyce, personal communication)

University of Pennsylvania Smell Test – This is a measurement of the individual's ability to detect odours at a supra-threshold level (94). The test takes only a few minutes. The test consists of 4 different 10-page booklets, with a total of 40 questions. On each page, there is a different "scratch and sniff" strip, which is embedded with a microencapsulated odorant. There is also a four choice multiple choice question on each page. The scents are released using a pencil. After each scent is released, the patient smells the level, and detects the odour from the four choices. There is an answer column on the back of

For peer review only - http://bmjopen.bmj.com/site/about/guidelines.xhtml

the test booklet, and the test is scored out of 40 items. The score is compared to scores in a normative database from 4000 normal individuals (95) (Additional optional module).

The REM-Behaviour Disorder Screening Questionnaire (RBDSQ) – This test is a validated 10-item patient self-rating questionnaire (maximum total score 13 points) covering the clinical features of REM Behavioral Disorders (RBD) (96). The RBDSQ was validated on 54 patients with polysomnographically confirmed RBD, 160 control subjects in whom RBD was excluded by history and polysomnography, and 133 unselected healthy subjects. Applying a positivity threshold of 5 points, the RBDSQ had a sensitivity of 0.96 and a specificity of 0.56 comparing patients with RBD with patients with other sleep disturbances. A specificity of 0.92 was calculated when comparing patients with RBD with patients without sleep disturbances (96) (Additional optional module).

Blood and saliva sample collection, and bio-banking

Participants seen in the research clinics will be asked to donate a blood sample for testing for tau protein, neurofilament (Nf) levels, full blood count analysis, and to obtain data for a GWAS. Samples of serum, plasma, and DNA will be collected and processed according to a pre-specified protocol and stored in a freezer at -80 °C at the Blizard Institute (Queen Mary, University of London). All participants will also asked to donate a blood sample through a dried blood spot test and a saliva sample (in order to obtain DNA where phlebotomy is not possible).

Samples will be tested to measure the expression of NfL (Neurofilament light) , a Nf-subunit showing consistent and reproducible measurements in plasma/serum using a newly developed sensitive methodology exploiting single molecule trapping and measurement by ELISA (SIMOA) (97-99). The use of both NfL and NfH (heavy) allows for the determination of different markers whose dynamic of release into bio fluids following injury vary according to their particular chemical structure. NfL has shown good levels of linearity on dilution experiments, suggesting that confounders like aggregation or immune response may not interfere with its measurements. NfH different phosphorylation states in blood may in turn provide more information on the systemic biological changes induced by trauma. The relative low abundance of these proteins in blood, in absence of CSF, which is normally more enriched in by-products of neuronal destruction, will not represent a major obstacle, as analytical sensitivity has evolved with the development of novel assays which cover the lower end of these protein dynamic range. SIMOA, the proposed methodology for the target biomarkers, is now emerging as the core

For peer review only - http://bmjopen.bmj.com/site/about/guidelines.xhtml

technique for the measurement of structural proteins, down to the fempto-molar end of the spectrum. A novel SIMOA-based assay for the measurement of Tau in blood is now available. This development offers a wide range of opportunities based on the possibility of overcoming the limitations imposed by CSF collection by lumbar puncture.

GWAS will be conducted at UCL in the lab led by Prof John Hardy.

Multimedia database set up and management

A multimedia database will be established as part of this project. The final dataset will include questionnaire and tests data (see Figure 1), in addition to the video-recorded neurological examination. This data will be also linked to the stored blood samples through a unique identifier.

The data will be stored in mirror, anonymised datasets at LSHTM and QMUL on secure servers, and access will be regulated by the study PIs who are ultimately responsible for maintaining confidentiality and data protection. Only the researcher(s) employed on this study will have access to subject identifiable information. Identifiable information (name, date of birth, and address) will be removed prior to analysis, and subjects will be identified by pseudoanonymised codes. Personal data and key codes for pseudoanonymisation will be stored in a database with restricted access. Before conducting the statistical analyses, checks will undertaken to ensure data quality.

Statistical analysis

Descriptive statistical methods (geometric means, geometric standard deviations, and 95% Confidence Intervals (95% C.I.) will be used to summarise the rugby history and lifestyle exposure data. Analyses linking a history of head trauma with neurological symptoms (obtained by questionnaire and neurobehavioural tests) will then be performed. Parameters of physical and cognitive capability, alongside clinical neurological intermediate endpoints, will initially be dichotomised (yes/no symptoms), and analysed with prevalence odds ratios using logistic regression (100). We will also analyse continuous outcome measures using multiple linear regression. In addition to analysing individual symptoms, we will assess associations with domains of symptoms (i.e. physical and cognitive capability). For this purpose we will use cut-points previously published in the literature (101), particularly related to the 1946 Birth Cohort whose testing protocol was used. For analyses involving repeated

For peer review only - http://bmjopen.bmj.com/site/about/guidelines.xhtml

measurements (neurobehavioural testing) assessing acute and chronic effects, we will use multilevel models. Analyses will be adjusted for age, sex, and alcohol consumption.

Finally, we will compare neurological outcomes assessed by questionnaire with those assessed by computer-administered tests using Kappa-statistics (and in case of continuous outcomes linear regression analyses). We will also compare the strengths of the associations with exposure between both outcome measures.

Study size and power

We aim to invite 205 participants to participate, approximately half of whom have been exposed to concussion during their career (M Cross, personal communication). We conservatively estimate that about 150 former players will participate. Based on previous studies, the standard deviations of the psychometric tests are in the range of 8-15% of the absolute value; assuming a conservative figure of 15% overall, the study will have more than 95% power to detect a 10% difference, and 80% power to detect a 7% difference, in psychometric test scores, between exposed (to concussion) and non-exposed participants.

Limitations

The main limitation of this study is the cross-sectional design. Data on lifetime exposure (history of concussion) and health outcomes are collected at one time point, making data subject to recall bias. Participants with a poorer health, or sentiment of discontent with their health status, may overestimate concussive exposure, if they attribute their poor health to it.

However, this cross-sectional study design will provide meaningful data on the burden of ill health and neurological health among retired rugby players, in a relatively short two-year time frame. Depending on results (and availability of additional funding), this study could inform a, more detailed, and time intensive cohort study, aimed at assessing the association between concussion in rugby players and health outcomes prospectively.

A direct consequence of the cross-sectional design is an intrinsic risk of selection bias. Rugby players who have developed serious neurodegenerative conditions (or have died from them) in the meantime are less likely (or not able) to participate in the study thus limiting our sample to a subsample of healthier retired rugby players. On the other hand, it is possible that former players who experience

For peer review only - http://bmjopen.bmj.com/site/about/guidelines.xhtml

some cognitive symptoms will be more keen to participate in the study, to seek reassurance and be examined in more detail.

Irrespective of these considerations, our investigation of the association between the exposure to sporting concussion and later-life general and neurological health outcomes remains valid, despite a smaller range of health outcomes diminishing the power for detecting an association as significant. Nonetheless, the power calculation for this study is calculated on the basis of continuous outcome measures (i.e. neurocognitive tests or grip strength measurement) making this study adequately powered to detect the potential associations, based on the expected number of recruited participants.

It is worth noting that results from this study will be not immediately apply to current rugby players, due to this cohort's former playing status, history of amateur playing exposure and alterations to rules and conditions, aimed at increasing plaer safety over recent years, which will have altered player's overall exposure to concussive events..

Ethics and dissemination

Ethical approval has been granted from the London School of Hygiene and Tropical Medicine (LSHTM) Ethics Committee. The study in general is focusing on subclinical problems (e.g. slightly lower than average scores in cognitive function tests) which would not usually require any medical attention. If any problems are identified, we will (with the permission of the study participant) refer participants to their primary care physician. The consent form includes the following statement:

- ☐ *I wish to inform my GP that I am taking part to this study*
- ☐ *I wish the study team to inform my GP of any unusual test result*

This study will provide unique insight into the physical and neurological health of retired rugby players who are now aged 50 years or more. This is valuable information *per se*, potentially comparable with other occupational cohorts or the general population (accepting the potential for bias discussed previously in comparing these samples). Moreover, within this study, data will be analysed as a function of the history of traumatic brain injury and concussion during their careers, and therefore assess associations between the history of reported concussive injuries and health-related outcomes.

For peer review only - http://bmjopen.bmj.com/site/about/guidelines.xhtml

Importantly, the neurological examination and collection of intermediate neurological outcomes, allows the detection of subtle preclinical changes that might be associated with future risk of developing a neurodegenerative condition. Studying to what extent these changes are also associated with history of concussion will allow an estimation of the impact of concussion on the onset of neurodegenerative diseases in rugby players, thus providing the basis for future prospective studies.

A novel aspect of this project is the molecular component: clinical and neurocognitive findings will be particularly reinforced by the measurement of blood biomarkers. These will give a quantitative measurement of active neurodegeneration (NfL) and of possible CTE (tau), to be analysed conjunctly with the health-related outcomes and personal history of concussion.

Also, relying on a number characteristics of the rugby playing history collected (i.e. length of playing at elite level, position played, numbers of game played, age when started playing), it will be possible to identify potential categories of players at potentially increased risks of health-related outcomes.

This study may identify a small deviation from normality in any of the continuous measures collected via cognitive and other tests (i.e. grip strength, memory function, walking speed, etc). This will be mainly investigated in association with a history of frequent concussions, or other head traumas. The investigators will interpret the results based on the continuum of distribution of the test outcomes, which will not have specific clinical translation. However, it should be emphasised that although this study is unlikely to identify patterns suggesting a clinical disease in any of the participants, if these are identified, then the relevant participant(s) will be referred to appropriate medical services.

These data will be disseminated initially to players and sports governing bodies, in addition to through peer reviewed publication and conference presentation, in order to begin to establish an evidence basis for the association between exposure to sports-related concussion, and potential later life cognitive and neurological impairment.

Conclusions

Here we have here presented the protocol for a cross-sectional study of history of concussion during a rugby union career, and subsequent measures of healthy ageing and subtle neurological and cognitive impairment. Although the study is focusing on rugby players, the study design, and methodology for the assessment of neurological health, are likely to be relevant for other studies of former professional sportspersons. Furthermore, cross-sectional studies of this type can also be conducted in current

For peer review only - http://bmjopen.bmj.com/site/about/guidelines.xhtml

players, and thus form the basis for prospective studies measuring alterations in neurological health over time.

For peer review only

For peer review only - http://bmjopen.bmj.com/site/about/guidelines.xhtml

References

1. Pearce N, Gallo V, McElvenny D. Head trauma in sport and neurodegenerative disease: an issue whose time has come? Neurobiol Aging. 2015;36(3):1383-9.
2. Lehman EJ. Epidemiology of neurodegeneration in American-style professional football players. Alzheimers Res Ther. 2013;5(4):34.
3. Chio A, Benzi G, Dossena M, Mutani R, Mora G. Severely increased risk of amyotrophic lateral sclerosis among Italian professional football players. Brain. 2005;128(Pt 3):472-6.
4. Belanger HG, Vanderploeg RD. The neuropsychological impact of sports-related concussion: a meta-analysis. J Int Neuropsychol Soc. 2005;11(4):345-57.
5. Kokmen E, Beard CM, O'Brien PC, Kurland LT. Epidemiology of dementia in Rochester, Minnesota. Mayo Clin Proc. 1996;71(3):275-82.
6. Nemetz PN, Leibson C, Naessens JM, Beard M, Kokmen E, Annegers JF, et al. Traumatic brain injury and time to onset of Alzheimer's disease: a population-based study. Am J Epidemiol. 1999;149(1):32-40.
7. Schofield PW, Tang M, Marder K, Bell K, Dooneief G, Chun M, et al. Alzheimer's disease after remote head injury: an incidence study. J Neurol Neurosurg Psychiatry. 1997;62(2):119-24.
8. Harris MA, Shen H, Marion SA, Tsui JK, Teschke K. Head injuries and Parkinson's disease in a case-control study. Occup Environ Med. 2013;70(12):839-44.
9. Rugbjerg K, Ritz B, Korbo L, Martinussen N, Olsen JH. Risk of Parkinson's disease after hospital contact for head injury: population based case-control study. BMJ. 2008;337:a2494.
10. Goldman SM, Tanner CM, Oakes D, Bhudhikanok GS, Gupta A, Langston JW. Head injury and Parkinson's disease risk in twins. Ann Neurol. 2006;60(1):65-72.
11. Seidler A, Hellenbrand W, Robra BP, Vieregge P, Nischan P, Joerg J, et al. Possible environmental, occupational, and other etiologic factors for Parkinson's disease: a case-control study in Germany. Neurology. 1996;46(5):1275-84.
12. Kuopio AM, Marttila RJ, Helenius H, Rinne UK. Environmental risk factors in Parkinson's disease. Mov Disord. 1999;14(6):928-39.
13. Bower JH, Maraganore DM, Peterson BJ, McDonnell SK, Ahlskog JE, Rocca WA. Head trauma preceding PD: a case-control study. Neurology. 2003;60(10):1610-5.
14. Lehman EJ, Hein MJ, Baron SL, Gersic CM. Neurodegenerative causes of death among retired National Football League players. Neurology. 2012;79(19):1970-4.
15. Savica R, Parisi JE, Wold LE, Josephs KA, Ahlskog JE. High school football and risk of neurodegeneration: a community-based study. Mayo Clin Proc. 2012;87(4):335-40.
16. Pupillo E, Messina P, Giussani G, Logroscino G, Zoccolella S, Chio A, et al. Physical activity and amyotrophic lateral sclerosis: a European population-based case-control study. Ann Neurol. 2014;75(5):708-16.
17. Belli S, Vanacore N. Proportionate mortality of Italian soccer players: is amyotrophic lateral sclerosis an occupational disease? Eur J Epidemiol. 2005;20(3):237-42.
18. Longstreth WT, McGuire V, Koepsell TD, Wang Y, van Belle G. Risk of amyotrophic lateral sclerosis and history of physical activity: a population-based case-control study. Arch Neurol. 1998;55(2):201-6.
19. Scarmeas N, Shih T, Stern Y, Ottman R, Rowland LP. Premorbid weight, body mass, and varsity athletics in ALS. Neurology. 2002;59(5):773-5.
20. DeKosky ST, Ikonomovic MD, Gandy S. Traumatic brain injury--football, warfare, and long-term effects. N Engl J Med. 2010;363(14):1293-6.

For peer review only - http://bmjopen.bmj.com/site/about/guidelines.xhtml

21. McKee AC, Cantu RC, Nowinski CJ, Hedley-Whyte ET, Gavett BE, Budson AE, et al. Chronic traumatic encephalopathy in athletes: progressive tauopathy after repetitive head injury. J Neuropathol Exp Neurol. 2009;68(7):709-35.
22. McKee AC, Stern RA, Nowinski CJ, Stein TD, Alvarez VE, Daneshvar DH, et al. The spectrum of disease in chronic traumatic encephalopathy. Brain. 2013;136(Pt 1):43-64.
23. Decq P, Gault N, Blandeau M, Kerdraon T, Berkal M, ElHelou A, et al. Long-term consequences of recurrent sports concussion. Acta Neurochir (Wien). 2016;158(2):289-300.
24. Hume PA, Theadom A, Lewis GN, Quarrie KL, Brown SR, Hill R, et al. A Comparison of Cognitive Function in Former Rugby Union Players Compared with Former Non-Contact-Sport Players and the Impact of Concussion History. Sports Med. 2016.
25. McMillan TM, McSkimming P, Wainman-Lefley J, Maclean LM, Hay J, McConnachie A, et al. Long-term health outcomes after exposure to repeated concussion in elite level: rugby union players. J Neurol Neurosurg Psychiatry. 2016.
26. Dubois B, Hampel H, Feldman HH, Scheltens P, Aisen P, Andrieu S, et al. Preclinical Alzheimer's disease: Definition, natural history, and diagnostic criteria. Alzheimers Dement. 2016;12(3):292-323.
27. Teasdale G, Jennett B. Assessment of coma and impaired consciousness. A practical scale. Lancet. 1974;2(7872):81-4.
28. Levin HS, Diaz-Arrastia RR. Diagnosis, prognosis, and clinical management of mild traumatic brain injury. Lancet Neurol. 2015;14(5):506-17.
29. Jordan BD. The clinical spectrum of sport-related traumatic brain injury. Nat Rev Neurol. 2013;9(4):222-30.
30. McCrory P, Meeuwisse WH, Aubry M, Cantu B, Dvorak J, Echemendia RJ, et al. Consensus statement on concussion in sport: the 4th International Conference on Concussion in Sport held in Zurich, November 2012. J Am Coll Surg. 2013;216(5):e55-71.
31. Giza CC, Kutcher JS, Ashwal S, Barth J, Getchius TS, Gioia GA, et al. Summary of evidence-based guideline update: evaluation and management of concussion in sports: report of the Guideline Development Subcommittee of the American Academy of Neurology. Neurology. 2013;80(24):2250-7.
32. Sharp DJ, Jenkins PO. Concussion is confusing us all. Pract Neurol. 2015;15(3):172-86.
33. Malec JF, Brown AW, Leibson CL, Flaada JT, Mandrekar JN, Diehl NN, et al. The mayo classification system for traumatic brain injury severity. J Neurotrauma. 2007;24(9):1417-24.
34. Jordan BD. Chronic traumatic encephalopathy and other long-term sequelae. Continuum (Minneap Minn). 2014;20(6 Sports Neurology):1588-604.
35. NIH. Traumatic Brain Injury (TBI) Definition.
36. Martland H. Punch drunk. JAMA. 1928;91:1103-7.
37. Hof PR, Bouras C, Buee L, Delacourte A, Perl DP, Morrison JH. Differential distribution of neurofibrillary tangles in the cerebral cortex of dementia pugilistica and Alzheimer's disease cases. Acta Neuropathol. 1992;85(1):23-30.
38. Gavett BE, Stern RA, Cantu RC, Nowinski CJ, McKee AC. Mild traumatic brain injury: a risk factor for neurodegeneration. Alzheimers Res Ther. 2010;2(3):18.
39. Omalu BI, Hamilton RL, Kamboh MI, DeKosky ST, Bailes J. Chronic traumatic encephalopathy (CTE) in a National Football League Player: Case report and emerging medicolegal practice questions. J Forensic Nurs. 2010;6(1):40-6.
40. Johnson VE, Stewart W, Graham DI, Stewart JE, Praestgaard AH, Smith DH. A neprilysin polymorphism and amyloid-beta plaques after traumatic brain injury. J Neurotrauma. 2009;26(8):1197-202.
41. Kilbourne M, Kuehn R, Tosun C, Caridi J, Keledjian K, Bochicchio G, et al. Novel model of frontal impact closed head injury in the rat. J Neurotrauma. 2009;26(12):2233-43.

For peer review only

For peer review only - http://bmjopen.bmj.com/site/about/guidelines.xhtml

42. Kirchhoff C, Leidel BA, Kirchhoff S, Braunstein V, Bogner V, Kreimeier U, et al. Analysis of N-terminal pro-B-type natriuretic peptide and cardiac index in multiple injured patients: a prospective cohort study. Crit Care. 2008;12(5):R118.
43. Papa L, Ramia MM, Edwards D, Johnson BD, Slobounov SM. Systematic review of clinical studies examining biomarkers of brain injury in athletes after sports-related concussion. J Neurotrauma. 2015;32(10):661-73.
44. Zetterberg H, Blennow K. Fluid biomarkers for mild traumatic brain injury and related conditions. Nat Rev Neurol. 2016;12(10):563-74.
45. Shahim P, Tegner Y, Gustafsson B, Gren M, Arlig J, Olsson M, et al. Neurochemical Aftermath of Repetitive Mild Traumatic Brain Injury. JAMA Neurol. 2016;73(11):1308-15.
46. Shahim P, Zetterberg H, Blennow K. Neurofilament Protein and Antineurofilament Antibodies Following Traumatic Brain Injury-Reply. JAMA Neurol. 2017;74(3):363-4.
47. Shahim P, Zetterberg H, Tegner Y, Blennow K. Serum neurofilament light as a biomarker for mild traumatic brain injury in contact sports. Neurology. 2017;88(19):1788-94.
48. Julien JP, Mushynski WE. Neurofilaments in health and disease. Prog Nucleic Acid Res Mol Biol. 1998;61:1-23.
49. Perrone Capano C, Pernas-Alonso R, di Porzio U. Neurofilament homeostasis and motoneurone degeneration. Bioessays. 2001;23(1):24-33.
50. Lu CH, Allen K, Oei F, Leoni E, Kuhle J, Tree T, et al. Systemic inflammatory response and neuromuscular involvement in amyotrophic lateral sclerosis. Neurol Neuroimmunol Neuroinflamm. 2016;3(4):e244.
51. Posmantur R, Hayes RL, Dixon CE, Taft WC. Neurofilament 68 and neurofilament 200 protein levels decrease after traumatic brain injury. J Neurotrauma. 1994;11(5):533-45.
52. Oliver J, Jones M, Kirk M, Gable D, Repshas J, Johnson T, et al. Serum Neurofilament Light in American Football Athletes Over the Course of a Season. J Neurotrauma. 2015.
53. Gatson JW, Barillas J, Hynan LS, Diaz-Arrastia R, Wolf SE, Minei JP. Detection of neurofilament-H in serum as a diagnostic tool to predict injury severity in patients who have suffered mild traumatic brain injury. J Neurosurg. 2014;121(5):1232-8.
54. Shahim P, Tegner Y, Wilson DH, Randall J, Skillback T, Pazooki D, et al. Blood biomarkers for brain injury in concussed professional ice hockey players. JAMA Neurol. 2014;71(6):684-92.
55. Shahim P, Tegner Y, Marklund N, Hoglund K, Portelius E, Brody DL, et al. Astroglial activation and altered amyloid metabolism in human repetitive concussion. Neurology. 2017;88(15):1400-7.
56. Shahim P, Gren M, Liman V, Andreasson U, Norgren N, Tegner Y, et al. Serum neurofilament light protein predicts clinical outcome in traumatic brain injury. Sci Rep. 2016;6:36791.
57. Morris M, Maeda S, Vossel K, Mucke L. The many faces of tau. Neuron. 2011;70(3):410-26.
58. Panenka WJ, Gardner AJ, Dretsch MN, Crynen GC, Crawford FC, Iverson GL. Systematic Review of Genetic Risk Factors for Sustaining a Mild Traumatic Brain Injury. J Neurotrauma. 2017;34(13):2093-9.
59. Mc Fie S, Abrahams S, Patricios J, Suter J, Posthumus M, September AV. The association between harm avoidance personality traits and self-reported concussion history in South African rugby union players. J Sci Med Sport. 2017.
60. Naj AC, Schellenberg GD, Alzheimer's Disease Genetics C. Genomic variants, genes, and pathways of Alzheimer's disease: An overview. Am J Med Genet B Neuropsychiatr Genet. 2017;174(1):5-26.
61. Redensek S, Trost M, Dolzan V. Genetic Determinants of Parkinson's Disease: Can They Help to Stratify the Patients Based on the Underlying Molecular Defect? Front Aging Neurosci. 2017;9:20.
62. Al-Chalabi A, van den Berg LH, Veldink J. Gene discovery in amyotrophic lateral sclerosis: implications for clinical management. Nat Rev Neurol. 2017;13(2):96-104.

For peer review only - http://bmjopen.bmj.com/site/about/guidelines.xhtml

63. Fuller GW, Kemp SP, Decq P. The International Rugby Board (IRB) Pitch Side Concussion Assessment trial: a pilot test accuracy study. British journal of sports medicine. 2015;49(8):529-35.
64. Hinton-Bayre AD, Geffen G, Friis P. Presentation and mechanisms of concussion in professional Rugby League Football. J Sci Med Sport. 2004;7(3):400-4.
65. Delaney JS, Al-Kashmiri A, Correa JA. Mechanisms of injury for concussions in university football, ice hockey, and soccer. Clin J Sport Med. 2014;24(3):233-7.
66. Davies MAM, A DJ, Delmestri A, S PTK, Stokes KA, Arden NK, et al. Health amongst former rugby union players: A cross-sectional study of morbidity and health-related quality of life. Sci Rep. 2017;7(1):11786.
67. Wadsworth M, Kuh D, Richards M, Hardy R. Cohort Profile: The 1946 National Birth Cohort (MRC National Survey of Health and Development). International journal of epidemiology. 2006;35(1):49-54.
68. Nofuji Y, Shinkai S, Taniguchi Y, Amano H, Nishi M, Murayama H, et al. Associations of Walking Speed, Grip Strength, and Standing Balance With Total and Cause-Specific Mortality in a General Population of Japanese Elders. J Am Med Dir Assoc. 2016;17(2):184 e1-7.
69. Cooper R, Kuh D, Hardy R. Objectively measured physical capability levels and mortality: systematic review and meta-analysis. BMJ. 2010;341:c4467.
70. Bohannon RW. Comfortable and maximum walking speed of adults aged 20-79 years: reference values and determinants. Age and ageing. 1997;26(1):15-9.
71. Schoon Y, Bongers K, Van Kempen J, Melis R, Olde Rikkert M. Gait speed as a test for monitoring frailty in community-dwelling older people has the highest diagnostic value compared to step length and chair rise time. Eur J Phys Rehabil Med. 2014;50(6):693-701.
72. Guralnik JM, Simonsick EM, Ferrucci L, Glynn RJ, Berkman LF, Blazer DG, et al. A short physical performance battery assessing lower extremity function: association with self-reported disability and prediction of mortality and nursing home admission. J Gerontol. 1994;49(2):M85-94.
73. Kuh D, Karunananthan S, Bergman H, Cooper R. A life-course approach to healthy ageing: maintaining physical capability. Proc Nutr Soc. 2014;73(2):237-48.
74. Roberts HC, Denison HJ, Martin HJ, Patel HP, Syddall H, Cooper C, et al. A review of the measurement of grip strength in clinical and epidemiological studies: towards a standardised approach. Age and ageing. 2011;40(4):423-9.
75. Jones CJ, Rikli RE, Beam WC. A 30-s chair-stand test as a measure of lower body strength in community-residing older adults. Res Q Exerc Sport. 1999;70(2):113-9.
76. Nilsdotter A, Bremander A. Measures of hip function and symptoms: Harris Hip Score (HHS), Hip Disability and Osteoarthritis Outcome Score (HOOS), Oxford Hip Score (OHS), Lequesne Index of Severity for Osteoarthritis of the Hip (LISOH), and American Academy of Orthopedic Surgeons (AAOS) Hip and Knee Questionnaire. Arthritis Care Res (Hoboken). 2011;63 Suppl 11:S200-7.
77. Collins NJ, Misra D, Felson DT, Crossley KM, Roos EM. Measures of knee function: International Knee Documentation Committee (IKDC) Subjective Knee Evaluation Form, Knee Injury and Osteoarthritis Outcome Score (KOOS), Knee Injury and Osteoarthritis Outcome Score Physical Function Short Form (KOOS-PS), Knee Outcome Survey Activities of Daily Living Scale (KOS-ADL), Lysholm Knee Scoring Scale, Oxford Knee Score (OKS), Western Ontario and McMaster Universities Osteoarthritis Index (WOMAC), Activity Rating Scale (ARS), and Tegner Activity Score (TAS). Arthritis Care Res (Hoboken). 2011;63 Suppl 11:S208-28.
78. Hamilton CB, Maly MR, Giffin JR, Clark JM, Speechley M, Petrella RJ, et al. Validation of the Questionnaire to Identify Knee Symptoms (QuIKS) using Rasch analysis. Health Qual Life Outcomes. 2015;13:157.
79. Folstein MF, Folstein SE, McHugh PR. "Mini-mental state". A practical method for grading the cognitive state of patients for the clinician. J Psychiatr Res. 1975;12(3):189-98.

For peer review only - http://bmjopen.bmj.com/site/about/guidelines.xhtml

80. Wechsler D. Wechsler Memory Scale - Revised Manual. Sant Antonio, TX: Psychological Corporation; 1987.
81. Papp KV, Amariglio RE, Dekhtyar M, Roy K, Wigman S, Bamfo R, et al. Development of a psychometrically equivalent short form of the Face-Name Associative Memory Exam for use along the early Alzheimer's disease trajectory. Clin Neuropsychol. 2014;28(5):771-85.
82. Rentz DM, Amariglio RE, Becker JA, Frey M, Olson LE, Frishe K, et al. Face-name associative memory performance is related to amyloid burden in normal elderly. Neuropsychologia. 2011;49(9):2776-83.
83. Aron AR, Monsell S, Sahakian BJ, Robbins TW. A componential analysis of task-switching deficits associated with lesions of left and right frontal cortex. Brain. 2004;127(Pt 7):1561-73.
84. Shallice T, Stuss DT, Picton TW, Alexander MP, Gillingham S. Multiple effects of prefrontal lesions on task-switching. Front Hum Neurosci. 2007;1:2.
85. Wechsler D. Wechsler Adult Intelligence Scale Sant Antonio, TX: Psychological Corporation; 1981.
86. Pertzov Y, Miller TD, Gorgoraptis N, Caine D, Schott JM, Butler C, et al. Binding deficits in memory following medial temporal lobe damage in patients with voltage-gated potassium channel complex antibody-associated limbic encephalitis. Brain. 2013;136(Pt 8):2474-85.
87. Parra MA, Abrahams S, Logie RH, Mendez LG, Lopera F, Della Sala S. Visual short-term memory binding deficits in familial Alzheimer's disease. Brain. 2010;133(9):2702-13.
88. Nelson HE, Willison J. The National Adult Reading Test (NART).
89. Say MJ, Jones R, Scahill RI, Dumas EM, Coleman A, Santos RC, et al. Visuomotor integration deficits precede clinical onset in Huntington's disease. Neuropsychologia. 2011;49(2):264-70.
90. Wechsler D. The Wechsler abbreviated scale of intelligence. San Antonio, TX: The Psychological Corporation; 1999.
91. Forster S, Lavie N. Entirely irrelevant distractors can capture and captivate attention. Psychon Bull Rev. 2011;18(6):1064-70.
92. Bremander AB, Dahl LL, Roos EM. Validity and reliability of functional performance tests in meniscectomized patients with or without knee osteoarthritis. Scand J Med Sci Sports. 2007;17(2):120-7.
93. Giovannoni G, van Schalkwyk J, Fritz VU, Lees AJ. Bradykinesia akinesia inco-ordination test (BRAIN TEST): an objective computerised assessment of upper limb motor function. J Neurol Neurosurg Psychiatry. 1999;67(5):624-9.
94. Doty RL, Shaman P, Dann M. Development of the University of Pennsylvania Smell Identification Test: a standardized microencapsulated test of olfactory function. Physiol Behav. 1984;32(3):489-502.
95. Doty RL, Frye RE, Agrawal U. Internal consistency reliability of the fractionated and whole University of Pennsylvania Smell Identification Test. Percept Psychophys. 1989;45(5):381-4.
96. Stiasny-Kolster K, Mayer G, Schafer S, Moller JC, Heinzel-Gutenbrunner M, Oertel WH. The REM sleep behavior disorder screening questionnaire--a new diagnostic instrument. Mov Disord. 2007;22(16):2386-93.
97. Gaiottino J, Norgren N, Dobson R, Topping J, Nissim A, Malaspina A, et al. Increased neurofilament light chain blood levels in neurodegenerative neurological diseases. PLoS One. 2013;8(9):e75091.
98. Kuhle J, Barro C, Andreasson U, Derfuss T, Lindberg R, Sandelius A, et al. Comparison of three analytical platforms for quantification of the neurofilament light chain in blood samples: ELISA, electrochemiluminescence immunoassay and Simoa. Clin Chem Lab Med. 2016;54(10):1655-61.
99. Rohrer JD, Woollacott IO, Dick KM, Brotherhood E, Gordon E, Fellows A, et al. Serum neurofilament light chain protein is a measure of disease intensity in frontotemporal dementia. Neurology. 2016;87(13):1329-36.

For peer review only - http://bmjopen.bmj.com/site/about/guidelines.xhtml

100. Pearce N. Effect measures in prevalence studies. Environ Health Perspect. 2004;112(10):1047-50.

101. Kaukiainen A, Hyvarinen HK, Akila R, Sainio M. Symptoms of chronic solvent encephalopathy: Euroquest questionnaire study. Neurotoxicology. 2009;30(6):1187-94.

For peer review only

Table 1: Glossary of definitions, adapted from (34)

**Chronic traumatic brain injury (TBI)**	A spectrum of disorders associated with long-term consequences of single or repetitive TBI
**Chronic traumatic encephalopathy (CTE)^**	Prototypic chronic TBI, long-term neurologic consequences of repetitive mild TBI
**Dementia pugilistica**	A subtype of CTE that is typically reserved for cases of severe end-stage dementia secondary to a long boxing career
**Chronic post-concussion syndrome^**	A condition in those athletes in whom post-concussion symptoms do not appear to resolve
**Chronic neurocognitive impairment^**	A rather diverse classification of chronic neurocognitive signs and symptoms secondary to head impact exposures and recurrent concussions that is theoretically distinctive from CTE
**Post-traumatic dementia***	Cases in which the athlete meets clinical criteria for dementia secondary to a single moderate to severe TBI
**Post-traumatic cognitive impairment***	Individuals who sustain long-term neurocognitive deficits from a single moderate to severe brain injury and do not meet clinical criteria for dementia, but instead mild cognitive impairment
**Post-traumatic parkinsonism**	A parkinsonian-like syndrome secondary to a single moderate to severe or repetitive TBI, occurring solely or as a component of CTE

^ the most clinically pertinent examples of sports-related chronic TBI ; *consequence of a single brain injury

For peer review only

For peer review only - http://bmjopen.bmj.com/site/about/guidelines.xhtml

Figure 1: Framework for data collection in the BRAIN study by domain

Footnote of Figure1:

*not to be collected if the participant is seen at home; ^subject to suitable chair availability for the participants seen at home; ~including knee bending test; #subject to space availability of the participant is seen at home; ≯to be administered as last item in all cases (after all 'Core' and Additional Optional module tests)

For peer review only

Authors' contributions: state how each author was involved in writing the protocol.

Author's contributions

Valentina Gallo – co-Principal Investigator. Drafted the protocol, collected comments, and act as corresponding author

Damien McElvenny – co-Principal Investigator. Contributed designing the protocol, reviewed intellectual content of this paper

Huw Morris – Co-investigator. Advised on cognitive testing, biomarkers, and clinical definitions, reviewed this paper for intellectual content

Sebastian Crutch – Co-investigator. Advised on cognitive testing, and clinical definitions, reviewed this paper for intellectual content

Simon Kemp – Co-investigator. Advised on recruitment strategy, patient and public involvement (PPI) and reviewed this paper for intellectual content

Catherine Hobbs – Research assistant of the BRAIN Study. Recruits and collects informations on the study participants

Donna Davoren - Project administrator of the BRAIN Study. Coordinates the logistics of the study

Nigel K. Arden – Co-investigator. Advised on recruitment strategy, patient and public involvement (PPI) and reviewed this paper for intellectual content

Andrea Malaspina – Co-investigator. Advised on biomarkers

Matt Cross - Co-investigator. Advised on recruitment strategy, patient and public involvement (PPI) and reviewed this paper for intellectual content

Madeleine A.M.Davies - Co-investigator. Advised on recruitment strategy, patient and public involvement (PPI), and reviewed this paper for intellectual content

Henrik Zetterberg – Co-investigator. Advised on biomarkers and reviewed this paper for intellectual content

Nick Fox - Co-investigator. Reviewed this paper for intellectual content

Neil Pearce – co-Principal Investigator. Contributed designing the protocol, reviewed intellectual content of this paper

Funding statement:

This study is funded by a grant from the Drake Foundation (www.drakefoundation.org/) to the London School of Hygiene and Tropical Medicine, with subcontracts to Queen Mary University of London (QMUL) and the Institute of Medicine (IoM).

For peer review only

Competing interest statement

None

Data sharing statement

The full protocol including all tests is available upon request to researchers intending conducting a similar study

For peer review only

For peer review only - http://bmjopen.bmj.com/site/about/guidelines.xhtml

	Questionnaire	Physical ability	Cognitive ability	Neurological examination	Intermediate neurological outcomes	Blood sample
**'Core' Module**	•Lifestyle and confounders questionnaire<br>•Concussion history questionnaire≭	•Height<br>•Weight<br>•Grip strength<br>•Chair rise^<br>•Walking speed#<br>•Photograph of hands	•MMSE<br>•WMS-R logical memory<br>•FNAME-12A<br>•Task-set shifting/Response inhibition<br>•WAIS-R digit symbol<br>•Visual short-term memory binding<br>•NART	•Video-recording~	•BRAIN test	•DNA*<br>•Plasma*
**Additional Optional Module**	•Pain mannequin (hand)	• HOOS<br>•KOOS<br>•QuIKS	•Visuomotor integration<br>•Matrix reasoning (WASI)<br>•Irrelevant Distractor Paradigm	•UPDRS-II	•Smell test<br>•RBD questionnaire	

Framework for data collection in the BRAIN study by domain

1122x640mm (120 x 120 DPI)

review only

STROBE Statement—Checklist of items that should be included in reports of ***cross-sectional studies***

	Item No	Recommendation	
**Title and abstract**	1	(*a*) Indicate the study's design with a commonly used term in the title or the abstract	OK
		(*b*) Provide in the abstract an informative and balanced summary of what was done and what was found	OK
**Introduction**			
Background/rationale	2	Explain the scientific background and rationale for the investigation being reported	OK, pp. 3-9
Objectives	3	State specific objectives, including any prespecified hypotheses	OK, pp. 9-10
**Methods**			
Study design	4	Present key elements of study design early in the paper	OK, pp. 10-11
Setting	5	Describe the setting, locations, and relevant dates, including periods of recruitment, exposure, follow-up, and data collection	OK, p. 10 as part of study design
Participants	6	(*a*) Give the eligibility criteria, and the sources and methods of selection of participants	OK, p.9
Variables	7	Clearly define all outcomes, exposures, predictors, potential confounders, and effect modifiers. Give diagnostic criteria, if applicable	OK, pp.10-18
Data sources/ measurement	8*	For each variable of interest, give sources of data and details of methods of assessment (measurement). Describe comparability of assessment methods if there is more than one group	OK, pp.10-18
Bias	9	Describe any efforts to address potential sources of bias	
Study size	10	Explain how the study size was arrived at	OK, pp.19-20
Quantitative variables	11	Explain how quantitative variables were handled in the analyses. If applicable, describe which groupings were chosen and why	OK, pp18-19
Statistical methods	12	(*a*) Describe all statistical methods, including those used to control for confounding	pp.18-19
		(*b*) Describe any methods used to examine subgroups and interactions	pp.18-19
		(*c*) Explain how missing data were addressed	TBD
		(*d*) If applicable, describe analytical methods taking account of sampling strategy	NA
		(*e*) Describe any sensitivity analyses	TBD
**Results**			
Participants	13*	(a) Report numbers of individuals at each stage of study—eg numbers potentially eligible, examined for eligibility, confirmed eligible, included in the study, completing follow-up, and analysed	NA
		(b) Give reasons for non-participation at each stage	NA
		(c) Consider use of a flow diagram	NA
Descriptive data	14*	(a) Give characteristics of study participants (eg demographic, clinical, social) and information on exposures and potential confounders	NA
		(b) Indicate number of participants with missing data for each variable of interest	NA
Outcome data	15*	Report numbers of outcome events or summary measures	NA

For peer review only

Main results	16	(*a*) Give unadjusted estimates and, if applicable, confounder-adjusted estimates and their precision (eg, 95% confidence interval). Make clear which confounders were adjusted for and why they were included	NA
		(*b*) Report category boundaries when continuous variables were categorized	NA
		(*c*) If relevant, consider translating estimates of relative risk into absolute risk for a meaningful time period	NA
Other analyses	17	Report other analyses done—eg analyses of subgroups and interactions, and sensitivity analyses	NA
**Discussion**			
Key results	18	Summarise key results with reference to study objectives	NA
Limitations	19	Discuss limitations of the study, taking into account sources of potential bias or imprecision. Discuss both direction and magnitude of any potential bias	pp.19-20
Interpretation	20	Give a cautious overall interpretation of results considering objectives, limitations, multiplicity of analyses, results from similar studies, and other relevant evidence	NA
Generalisability	21	Discuss the generalisability (external validity) of the study results	P. 21
**Other information**			
Funding	22	Give the source of funding and the role of the funders for the present study and, if applicable, for the original study on which the present article is based	P.29

*Give information separately for exposed and unexposed groups.

Note: An Explanation and Elaboration article discusses each checklist item and gives methodological background and published examples of transparent reporting. The STROBE checklist is best used in conjunction with this article (freely available on the Web sites of PLoS Medicine at http://www.plosmedicine.org/, Annals of Internal Medicine at http://www.annals.org/, and Epidemiology at http://www.epidem.com/). Information on the STROBE Initiative is available at www.strobe-statement.org.

For peer review only

BRain health and healthy AgeINg in retired Rugby union players, the BRAIN study – study protocol for an observational study in the UK

Journal:	*BMJ Open*
Manuscript ID	bmjopen-2017-017990.R2
Article Type:	Protocol
Date Submitted by the Author:	20-Oct-2017

Complete List of Authors:	Gallo, Valentina; Queen Mary, University of London, Centre for Primary Care and Public Health<br>McElvenny, Damien; Institute of Occupational Medicine,<br>Hobbs, Catherine; London School of Hygiene and Tropical Medicine Faculty of Epidemiology and Population Health<br>Davoren, Donna; London School of Hygiene and Tropical Medicine Faculty of Epidemiology and Population Health<br>Morris, Huw ; UCL<br>Crutch, Sebastian; University College London Institute of Neurology<br>Zetterberg, Henrik; Institute of Neuroscience and Physiology, Sahlgrenska Academy, University of Gothenburg., Department of Psychiatry and Neurochemistry<br>Fox, Nick; UCL, Dementia Research Centre<br>Kemp, Simon<br>Cross, Matthew; Rugby Football Union<br>Arden, Nigel; Nuffield Department of Orthopaedics, ;<br>Davies, Madeleine; University of Oxford, Nuffield Department of Orthopaedics, Rheumatology and Musculoskeletal Science<br>Malaspina, Andrea; Queen Mary University of London<br>Pearce, Neil; London School of Hygiene and Tropical Medicine

**Primary Subject Heading**:	Sports and exercise medicine
Secondary Subject Heading:	Epidemiology, Neurology
Keywords:	Rugby, concussion, Rugby Union, cross-sectional study, cognitive function, healthy ageing

SCHOLARONE™ Manuscripts

BRain health and healthy AgeINg in retired Rugby union players, the BRAIN study – study protocol for an observational study in the UK

Valentina Gallo[1,2,3], Damien McElvenny[4] , Catherine Hobbs[3], Donna Davoren[3], Huw Morris[5], Sebastian Crutch[6], Henrik Zetterberg[7,8,9,10], Nick C Fox[6], Simon Kemp[11], Matt Cross[11], Nigel Arden[12], Madeleine A.M. Davies[12], Andrea Malaspina[13], Neil Pearce[3]

1. Centre for Primary Care and Public Health, Queen Mary University of London, London, UK
2. School of Public Health, Imperial College London, London, UK
3. London School of Hygiene and Tropical Medicine, London, UK
4. Institute of Occupational Medicine, Edinburgh, UK
5. University College London, London, UK
6. Dementia Research Centre, UCL Institute of Neurology, University College London, London, UK
7. Department of molecular neuroscience, UCL Institute of Neurology, Queen Square, London
8. UK Dementia Research Institute, London, UK
9. Clinical Neurochemistry Laboratory, Sahlgrenska University Hospital, Mölndal, Sweden
10. Department of Psychiatry and Neurochemistry, Institute of Neuroscience and Physiology, the Sahlgrenska Academy at the University of Gothenburg, Mölndal, Sweden
11. Rugby Football Union (RFU), London, UK
12. Arthritis Research UK Centre for Sport, exercise and osteoarthritis, University of Oxford, Oxford, UK
13. Department of Neuroscience and Trauma, Blizard Institute, Queen Mary, University of London, London, UK

Please address all correspondence to:

Dr Valentina Gallo

Centre for Primary Care and Public Health
Queen Mary, University of London
Yvonne Carter Building
58, Turner Street
E1 2AB London (UK)
valentina.gallo@lshtm.ac.uk or v.gallo@qmul.ac.uk

For peer review only

For peer review only - http://bmjopen.bmj.com/site/about/guidelines.xhtml

Abstract

Introduction - Relatively little is known about the long-term health of former elite rugby players, or former sportspeople more generally. As well as the potential benefits of being former professional sportspersons, there may be potential health risks from exposures occurring during an individual's playing career, as well as following retirement. Each contact sport has vastly different playing dynamics, therefore exposing its players to different types of potential traumas. Current evidence suggests that these are not necessarily comparable in terms of pathophysiology, and their potential long-term adverse effects might also differ. There is currently limited but increasing evidence that poorer age-related and neurological health exists among former professional sportsmen exposed to repetitive concussions; however the evidence is limited on rugby union players, specifically.

Methods and analysis – We present the protocol for a cross-sectional study to assess the association between self-reported history of concussion during the playing career, and subsequent measures of healthy ageing and subtle neurological and cognitive impairment. We are recruiting a sample of approximately 200 retired rugby players (former Oxford and Cambridge University rugby players and members of the England Rugby International Club) aged 50 years or more, and collecting a number of general and neurological health-related outcome measures though validated assessments. Biomarkers of neurodegeneration (neurofilaments and tau) will be also be measured. Although the study is focusing on rugby union players specifically, the general study design and the methods for assessing neurological health are likely to be relevant to other studies of former professional sportspersons.

Ethics and dissemination – The study has been approved by the Ethical Committee of London School of Hygiene and Tropical Medicine (LSHTM) (reference: 11634 [illegible] 2). It is intended that results of this study will be published in peer reviewed medical journals, communicated to participants, the general public, and all relevant stakeholders.

Strengths and limitations

- The study will provide meaningful data on the burden of health and neurological health among retired rugby players (in only two-year's time)
- The cross-sectional design does not allow preventing from the potential for recall bias and selection bias
- Results will not be immediately generalizable to current players as in the last 30 years playing rules and conditions are changed, increasing the player safety

For peer review only - http://bmjopen.bmj.com/site/about/guidelines.xhtml

Keywords:

Rugby, concussion, Rugby Union, cross-sectional study, cognitive function, healthy ageing, neurological health, sport

For peer review only

Introduction

The evidence relating to head trauma in sport and the subsequent risks of neurological disease have previously reviewed, and it has been established that sports involving repeated head trauma may have an increased risks of neurodegenerative disease in the long-term (1). Furthermore, there are now plausible mechanisms for these effects, and a recognition that these problems do not just occur in former boxers, but in a variety of sports involving repeated concussions (2), and possibly also in sports in which low-level head trauma is common (3). These neurodegenerative effects include potentially increased risks of impaired cognitive function and dementia (4-7), Parkinson's disease (PD) (8-13), and amyotrophic lateral sclerosis (ALS) (3, 14-19). The term *chronic traumatic encephalopathy* (CTE) was introduced as a clinical-pathological construct for the neurodegeneration associated with American football and wrestling (20) (see Table 1).

Each contact sport has vastly different playing dynamics therefore exposing its players to different types of potential traumas. Current evidence suggests that these are not necessarily comparable in terms of pathophysiology, and hence in terms of their potential long-term adverse effects on health. There is currently limited but increasing evidence that poorer general and neurological health exists among professional sportsmen exposed to repetitive concussions; however, there is little evidence from rugby union players (21-23).

Decq et al (21) investigated retired French-speaking high-level sportsmen, aged 45 to 65 years, who had played sports for at least 10 years. Mild cognitive disorder was lower in players of other sports (40.4%) than in former rugby players (56.6%) (p=0.005). However, after adjustment for smoking and higher education, no association was observed between cognitive function and number of reported concussions (21). In New Zealand, 366 former players were tested on their engagement in sport, general health, sports injuries and concussion history, demographic information and cognitive functioning. The elite rugby group performed worse on tests of complex attention, processing speed, executive functioning, and cognitive flexibility than the non-contact-sport group, and worse than the community rugby group on complex attention. Former players who recalled one or more concussions had worse scores on cognitive flexibility, executive functioning, and complex attention than players who did not recall experiencing a concussion (22). A recent Scottish study assessed 52 former Scottish international rugby players and 29 controls. Players performed worse on a test of verbal learning, and of fine co-ordination of the dominant hand; however, no statistically significant differences were observed on

For peer review only - http://bmjopen.bmj.com/site/about/guidelines.xhtml

other cognitive tests. Additionally, no significant association was found between the number of concussions and cognitive test performance (23).

Ultimately, establishing the extent of these potential issues will require long-term prospective studies involving the repeated measurement to head trauma exposures and repeated tests of neurological health in large numbers of current and former players. However, a first step in this process is to conduct cross-sectional studies in former players to assess whether there is an association between the history of concussion during their rugby career, and subsequent measures of healthy ageing and subtle neurological and cognitive impairment. In particular, such cross-sectional studies can be conducted in a relatively short time and with 'standard' measures of cognitive function since impaired cognitive function is an important health outcome in itself, and may be a precursor of more serious long-term neurological effects (24).

Definitions of trauma and concussion

Traumatic brain injury (TBI) is usually classified as mild, moderate, or severe, on the basis of the initial Glasgow Coma Scale (GCS) (25) recorded in the Emergency Departments including the duration of any loss of consciousness; and duration of post-traumatic amnesia (i.e., loss of memory of events after the injury) (26). Chronic TBI represents a spectrum of disorders associated with long-term consequences after single or repetitive TBI (27). Conversely, there remains no consensus on the definition of concussion. The 2012 Zurich Consensus Statement on Concussion in Sport proposed that concussion and mild TBI should be viewed as distinct entities (28). The group defined concussion as a "complex pathophysiological process affecting the brain", and despite allowing for the presence of neuropathological damage, they postulated that concussive symptoms largely reflected a functional disturbance, typically resolving spontaneously with no imaging abnormality. In contrast, recent guidelines from the American Academy of Neurology for sports concussion in 2013, do not separate concussion from mild TBI, defining concussion as "a clinical syndrome of biomechanically induced alteration of brain function, typically affecting memory and orientation, which may involve loss of consciousness" (29). A recent report (30) examines why having two different pathological entities might be unhelpful and suggests that the Mayo Clinic TBI Classification system should cover both definitions(31). A glossary of definitions used in this protocol is found in Table 1, adapted from Jordan (2014) (32).

For peer review only - http://bmjopen.bmj.com/site/about/guidelines.xhtml

For the purpose of this study, we adapted the NIH definition of concussion (33). Participants will be asked to report their previous concussions according with the following definition:

Concussion is defined as an alteration in brain function, caused by an external force. Symptoms include:

- *A decreased level / loss of consciousness*
- *Memory Loss (before or after the injury)*
- *Weakness*
- *Temporary Paralysis*
- *Loss of balance*
- *Change in vision (e.g. blurriness, double vision)*
- *Co-ordination difficulties*
- *Numbness*
- *Decreased sense of smell*
- *Difficulty understanding what others are saying*
- *Difficulty communicating with others*
- *Confusion, disorientation, or slowed thinking*

Please note, loss of consciousness is not required for a concussion to be diagnosed.

Biomarkers

Research in the field of TBI biomarkers has increased exponentially over the last 20 years (27, 34-36), with studies assessing biomarkers that could provide diagnostic and prognostic, as well as monitoring information(37, 38). However, to date, no biomarker for the long-term effects of concussion has been identified, although recent studies on cerebrospinal fluid (CSF) from sportsmen with post-concussion syndrome suggest that a subset of these have biomarker signs of ongoing axonal injury and microglial activation (39-41).

Of the biomarkers measurable in serum or other body fluids, those more likely to be detectable for longer time periods after the concussion episode, are those related to the axonal injury and more in generally neuroinflammation, that is neurofilament and tau protein (38). Neurofilaments (NF) co-assemble from protein subunits to form NFs defined as light (light (NF-L), medium (NF-M), or heavy (NF-H) according to their relative molecular weights). NFs are one of the key structural elements of neurons,

For peer review only - http://bmjopen.bmj.com/site/about/guidelines.xhtml

providing mechanical stability and determining axonal diameter. NFs are of particular interest because as they are structural elements, they may be more susceptible to mechanical deformation under the condition of trauma. Abnormal NF aggregation may contribute to the delayed or progressive neuronal death and dysfunction taking place in neurodegenerative diseases associated with NF aggregation, such as PD, ALS, and Lewy body dementia (LBD) (42-44). Acute perturbations of NFs have been demonstrated in experimental models of TBI that produce cortical contusion in combination with selective hippocampal neuronal death (45). A study compared serum NF-L, a biological marker of head trauma, in American football athletes and non-contact sport athletes and examined changes over the course of a season. Results suggest that a season of collegiate American football is associated with elevations in serum NF-L, which is indicative of axonal injury, resultant of head impacts (46). NF-L has also been associated with head trauma severity detected by CT scan, immediately after the episode (47) and with concussive and subconcussive head impacts in boxing (41). Importantly, whilst plasma tau concentration increases and disappears rapidly (within hours to a few days) following concussion (48), NF-L has a prolonged increased levels lasting for many weeks (41, 49, 50).

Phosphorylation of tau is a normal event in healthy neurons, but hyperphosphorylation and aggregation into neurofibrillary tangles is a characteristic of Alzheimer's disease and CTE (51). Tau concentrations correlate with lesion size and outcome in severe TBI when measured in the ventricular CSF, while remaining unchanged in TBI and other forms of acute brain injury (38). Studies on mild TBI show increased CSF concentrations of both t[illegible]tau and NF[illegible]L, although the increase in CSF NF-L is greater than the increase in t-tau, suggesting that head impacts have a greater effect on long, large-calibre axons that extend subcortically than on short, nonmyelinated axons in the cortex (38). After concussion, serum tau concentrations were found to be increased, but this increase did not correlate with severity of trauma and lesion load as measured using CT scan signs (38).

Genetics

Few hypotheses on the potential role of genetics in modulating the possible association between concussion and cognitive decline have been formulated. Specific genes might be associated with an increased risk of concussion via worse attention or executive function, or more vulnerable brain anatomy (52), or via personality trait (53). Conversely, a number of genes are involved in modulating the risk of developing dementia and other neurodegenerative diseases, irrespective of concussion. Dementia risk is higher amongst carriers of the epsilon4 allele at the Apolipoprotein E (APOE) gene (54).

For peer review only - http://bmjopen.bmj.com/site/about/guidelines.xhtml

A number of Genome Wide Association Studies (GWAS) has been performed to date, enabling the identification of 24 loci as risk factors for Parkinson's disease.

Rationale of the BRAIN Study

Recently, World Rugby (formally known as the International Rugby Board), has developed a process to support team clinicians in the recognition, assessment, and subsequent management of elite adult players who have sustained a potential concussion. This process includes the development of a multimodal assessment: the Head Injury Assessment, formerly the Pitch Side Concussion Assessment (PSCA) tool (55).

However, there is little clear evidence, about the possible long-term effects of concussion in rugby union players, and it is not clear if findings from other sports are directly generalizable to rugby players. Many sports involve exposure to concussion or repetitive low-level head trauma, and it can be argued that each sport should be considered independently, due to the unique technical and physiological profile that a player develops over the course of a career (56, 57). Thus, to determine the potential risk of long-term adverse health effects of playing rugby specific studies of rugby players are needed.

The BRAIN Study builds upon a recent cross-sectional questionnaire-based study conducted by University of Oxford as part of the Arthritis Research UK sport, exercise and osteoarthritis centre, who have assessed the general and musculoskeletal health of former elite rugby players (58). Rugby players from the Oxford and Cambridge University Football Clubs (Oxbridge Blues), and members of the England Rugby Internationals Club (ERIC) – a membership organization of all current and former England players - were recruited. Self-reported demographic factors, playing history (including head trauma), past medical history (including dementia, depression, and memory impairment), and perceived health were collected, in addition to detailed information on musculoskeletal health and pain.

A total of 319 participants have participated in this study to date, and 205 of those aged 50 years or older have agreed to be re-contacted for further studies. The 205 participants aged 50+ years who agreed to be re-contacted will be invited to take part to the BRAIN Study.

For peer review only - http://bmjopen.bmj.com/site/about/guidelines.xhtml

Aim and objectives

The overall aim of the BRAIN study is to investigate the possible associations between concussion in rugby and ageing, including physical and cognitive capabilities, as well intermediate neurological and musculoskeletal endpoints among former rugby players.

In order to achieve these aims the following objectives will be pursued:

1. To investigate the associations between self-reported concussion history and ageing, measured as physical and cognitive capabilities. This will be achieved by using the following outcome measures:
    a. Physical capability outcomes: grip strength, chair raise and walking speed
    b. Cognitive capability outcomes: memory, reasoning, speed of thinking and attention, and verbal and numerical skills.
2. To investigate the association between self-reported concussion history and intermediate neurological endpoints
    a. Neurological examination: a brief neurological examination will be video-recorded according to existing protocols and independently examined by two neurologists
    b. Intermediate neurological endpoints: information using tapping test (BRAIN), smell test, REM-behaviour disorder questionnaire will be collected in order to explore non motor symptoms of Parkinson's disease and related disorders
3. To investigate whether a history of concussion is associated with current tau protein and NF-L levels in blood (potential biomarkers of neurodegeneration), and how these biomarkers are in turn associated with the outcome measures (potential biomarkers of early detection of disease)
4. To assess if any other characteristic of rugby playing history, in addition to self-reported concussion (i.e. length of playing at elite level, position of play, numbers of game played, age when started playing), or age at concussion is associated with any outcome measure (physical and cognitive capability and/or intermediate neurological outcomes)
5. Investigate the long-term musculoskeletal health outcomes of rugby players with particular emphasis on hip, knee, and hand osteoarthritis allowing changes over time (from the previous study to the current study) to be assessed

For peer review only - http://bmjopen.bmj.com/site/about/guidelines.xhtml

In addition, we will develop a multimedia database (data from tests and questionnaire plus video-recorded neurological examination plus biobank of blood samples) that will serve as baseline for further tests and further follow-up.

Study design

Participants will be invited to study clinics in London, Manchester, or Bath. A home visit can also be arranged by request. Participants will be invited to bring with them any medication they are taking regularly for an accurate recording of current medication usage.

All participants will be administered a ***Core Module*** interview including questions on lifestyle factors, potential confounders, and extensive information on the five domains of physical and cognitive ability, neurological examination, intermediate neurological outcomes and musculoskeletal health.

The lifestyle questionnaire will complement that already administered to participants of the Oxford-base cross-sectional study, and will include questions on potential confounders of the association between concussion and physical and cognitive capability, and intermediate neurological outcomes. Participants will also be asked to donate a blood sample (a normal blood sample will be collected by participants seen in one of the clinics, a dry spot sample plus saliva swab for DNA will be collected from participants seen at home). At the end of the Core Module, all participants will be also asked to undergo some additional tests (the ***Additional Optional Module***) providing they have sufficient time (Figure 1). At the end of the interview, all participants will be asked some final questions on their concussion history. This will ensure that the interviewer is blind to the participant's concussion history during the entire duration of the interview, and will also act as validation to confirm data previously collected. Participants who disclose information about their concussion history during the interview, will be noted in order to undertake a sensitivity analysis excluding them.

The ***Core Module*** includes: 1) a lifestyle questionnaire; 2) a set of tests covering essential information on physical (height, weight, grip strength, chair rise, walking speed and photo of the hands) and cognitive capability (Mini-Mental State Examination, Logical Memory, Digit-Symbol Substitution test, Matrix Reasoning, Task-set Shifting/Response Inhibition, Visuomotor Integration, 12-item Face-Name Associative Memory Exam, and the National Adult Reading Test); 3) a remote neurological clinical

For peer review only - http://bmjopen.bmj.com/site/about/guidelines.xhtml

examination (video recorded); and 4) a test for subtle movement disorders, the BRAIN tapping test. This Core Module should take no longer of 1 hour and 45 minutes to be completed.

The ***Additional Optional Module*** includes: 1) a questionnaire investigating hand pain; 2) extra tests investigating the cognitive domain (Visual short-term memory binding, Irrelevant Distractor Paradigm); 2) the UPDRS part II scale to complete the neurological examination (not video-recorded); 3) additional tests for intermediate neurological outcomes (the smell test and the REM behaviour disorder questionnaire). This Additional Optional Module should take no longer than 1 hour and half to be completed (Figure 1). The full test/interview including core and additional module should not take more than 3 hours and 15 minutes to complete.

General Questionnaires

Questionnaires will be used to collect relevant information on:

Lifestyle and confounders– all participants will be asked a number of questions on their lifestyle in order to collect information on possible confounders for the main analysis (smoking, alcohol, coffee, drugs, past medical history, sleep quality).

Concussion history– at the end of the interview, participants will be re-asked some detailed information regarding their history of head trauma and concussion whilst playing.

Musculoskeletal hand pain – A questionnaire on hand pain to identify possible hand osteoarthritis will be utilized involving a hand mannequin (additional optional module).

Physical ability assessment

The methodology used in the 1946 birth cohort (59) will be leveraged to assess physical and cognitive capabilities in this study. This will enhance comparability of results and facilitate future collaborations. The most commonly used objective measures of physical capability for assessing healthy ageing, are tests of grip strength (60, 61), walking speed (60, 62, 63), chair rises (64) and standing balance (60); these aim to assess physical functioning, including the capacity to undertake the physical tasks of daily living (65). There is robust evidence that higher scores on these measures are associated with lower rates of mortality, and there is more limited evidence of lower risks of morbidity, and of age-related patterns of change (65).

For peer review only - http://bmjopen.bmj.com/site/about/guidelines.xhtml

Height and weight – height and weight will be collected in order to calculate the body mass index (BMI) according to the formula BMI = weight (Kg) / height $(m)^2$.

Grip strength - JAMAR Hydraulic Hand Dynamometer is the most widely used instrument with established test-retest, inter-rater and intra-rater reliability (66).

Chair rise - The 30-Second Chair Stand Test will be used to assess lower body strength (67). This test provides a reasonably reliable and valid indicator of lower body strength in generally active, community-dwelling older adults. Test-retest intra-class correlations of 0.84 for men and 0.92 for women, utilizing one-way analysis of variance procedures appropriate for a single trial, together with a non-significant change in scores from Day 1 testing to Day 2, indicate that the 30-s chair stand has good stability reliability (67). Moreover, a moderately high association between chair-stand performance and maximum weight-adjusted leg-press performance for both men and women (r = 0.78 and 0.71, respectively) supports the criterion-related validity of the chair stand as a measure of lower body strength (67). As expected, chair-stand performance decreased significantly across age groups in decades – from the 60s to the 70s to the 80s ($p < 0.01$) and was highly statistically significantly lower for low-active participants than for high-active participants ($p < 0.0001$) (67).

Walking speed – To assess normal comfortable walking speed and maximum walking speed, a Timed 10-Meter Walk Test will be used. This test has been validated in 230 healthy volunteers (62). Mean comfortable gait speed ranged from 127.2 cm/s for women in their seventies to 146.2 cm/s for men in their forties. Mean maximum gait speed ranged from 174.9 cm/s for women in their seventies to 253-3 cm/s for men in their twenties. Both gait speed measures were reliable (correlation coefficients > 0.903) and correlated significantly with age (r>-0.210), height (r>0.220) and the strengths of four measured lower extremity muscle actions (r = 0.190-0.500). These normative values give clinicians a reference against which patient performance can be compared in a variety of settings (62).

A photograph of the hands will be taken at the end of this section to assess inflammation, finger-nodes, and other rheumatological and osteoarthritis-related changes at the hand.

HOOS – The Hip Disability and Osteoarthritis Outcome Score (HOOS) measures patient's opinions about their hip and associated problems. It examines pain, symptoms, function in activities of daily living (ADL)

For peer review only

For peer review only - http://bmjopen.bmj.com/site/about/guidelines.xhtml

and function in sport and recreation. It has been used in subjects with hip disability with or without hip osteoarthritis (OA) (68) (Additional optional module).

KOOS – The Knee Injury and Osteoarthritis Outcome Score (KOOS) examines pain frequency and severity during functional activities, and symptoms such as the severity of knee stiffness, the presence of swelling, grinding or clicking, difficulty in ADL, with sport and recreation, and knee-related quality of life. It is intended for use in young and middle-aged populations with post-traumatic OA, in addition to those with injuries who may go on to develop secondary OA (69) (Additional optional module).

QuIKS – The Questionnaire to Identify Knee Symptoms (QuIKS) is used for early osteoarthritis, and understanding symptomology and potential adaptation to activity before osteoarthritis has been clinically diagnosed. It has been developed in adults aged 40-65 with evidence of ongoing knee problems and recommended for use in studies exploring early osteoarthritis (70) (Additional optional module).

Cognitive ability assessment

The methodology used in the 1946 birth cohort will also be followed for the assessment of cognitive capability (59). Tests and questionnaires will be described according their belonging to the Core Module or the Additional Optional Module, and by domain (Figure 1).

Mini-Mental State Examination (MMSE (71)) – The MMSE is a widely used 30-point screening tool for cognitive impairment within clinical practice, assessing multiple cognitive domains including orientation to time and place; registration; attention +/- calculation; recall; language; repetition; reading; writing; visuospatial function; and executive function and praxis.

Logical Memory from the ***Wechsler Memory Scale-Revised*** (WMS-R (72)) - The Logical Memory test assesses free recall of a short story. The participant is asked to recall the story immediately and after a 20minute delay.

12-item Face-Name Associative Memory Exam (FNAME-12A) – The FNAME-12A is a modified version of the 16-item Face-Name Associative Memory Exam (FNAME-16). The FNAME-12A has fewer stimuli and additional learning trials which have been found to be well tolerated by those with Mild Cognitive Impairment, whilst remaining challenging in cognitively normal older adults (73). The FNAME-12A has demonstrated psychometric equivalence with the FNAME-16, which has been shown to be related to beta-amyloid burden in cognitively normal elderly people (74). The FNAME-12A requires the participant

For peer review only - http://bmjopen.bmj.com/site/about/guidelines.xhtml

to learn 12 face-name and face-occupation pairs. Participants are given two exposures to all 12 face-name/occupation pairs. After each exposure, and following a 5-minute delay, participants are asked for the name and occupation associated with each face. After a 30-minute delay they are shown 3 faces and asked to identify the face that they recognise and give the name and occupation. Given multiple options to choose from, they are then asked to select the name and/or occupation associated with the face.

Task-set Shifting/Response Inhibition (75, 76) – The task-set shifting and response inhibition task examines the relationship between executive tasks of task-switching/preparation time. In the arrow only condition, participants are shown the cue 'arrow'. Following a short delay they must respond to the direction of the arrow ('right' or 'left'). In the word only condition, participants are shown the cue 'word'. Following a short delay they must respond to the direction of the word ('right' or 'left'). There is a switching condition in which the participant is shown the cue 'arrow' or 'word'. Following a delay, both a combined arrow and word stimulus appears. The stimulus is either congruent (left arrow and left word), or incongruent (left arrow and right word). Trials in the switching task are categorised into switch and non-switch. In a non-switch trial the cue is the same as for the immediately preceding trial. In a switch trial the cue differs from the immediately preceding trial.

Digit-Symbol Substitution Test, from the ***Wechsler Adult Intelligence Scale-Revised*** (WAIS-R (77)) - The Digit-Symbol Substitution test explores attention and psychomotor speed. Participants are given a code table displaying digits (from 1 to 9); each digit is paired with a symbol. The participant is required to complete in blank squares with the corresponding symbol for each digit as shown in the code table. They are given 90 seconds to fill in as many squares as possible.

Visual Short-term Memory Binding (78, 79) – This test requires the participant to view one or three fractal objects, presented simultaneously in random locations on the screen. The participant is asked to remember both the objects and their location. After a delay of 1 or 4 seconds they have to make a forced choice between one of the displayed fractals (the target) and a 'dummy' fractal. Participants are required to touch the object they think has been previously presented and 'drag' it on the touch screen to its remembered, original location. The binding of such featured information has been shown to be vulnerable in asymptomatic FAD mutation carriers (79).

National Adult Reading Test (NART) – The NART was specially designed to provide a means of estimating the premorbid intelligence levels of adults suspected of suffering from intellectual deterioration (80). The NART comprises a list of 50 words printed in order of increasing difficulty. The

For peer review only - http://bmjopen.bmj.com/site/about/guidelines.xhtml

words are relatively short in order to avoid the possible adverse effects of stimulus complexity on the reading of dementing subjects, and they are all 'irregular' with respect to the common rules of pronunciation in order to minimise the possibility of reading by phonemic decoding rather than word recognition (80).

Visuo-motor Integration (81) – This is a circle tracing task which includes both direct and indirect visual feedback conditions. Continuous performance measures are provided including accuracy, speed, and speed of error detection and correction. The test has revealed changes in speed and accuracy in Huntington Disease mutation carriers more than 10 years before expected age-of-onset (Additional optional module).

Matrix Reasoning from the ***Wechsler Abbreviated Scale of Intelligence*** (WASI (82)) – The Matrix Reasoning test assesses nonverbal reasoning. The participant is shown a matrix of geometric shapes with a section missing. They are required to select the option that completes the matrix (Additional optional module).

Irrelevant Distractor Paradigm (83) – Participants are given a computerised letter-search task and are required to make a rapid decision as to whether the target letter 'X' or 'N' has appeared in the search display (in either low or high load conditions). On some of the trials, a task-irrelevant distractor (a cartoon character) appears on the outside of the search display. The task evaluates the extent to which attention is captured and captivated by the distractor (Additional optional module).

Neurological clinical assessment

The neurological assessment of participants is needed for detecting any subtle neurological sign, and to establish a baseline for the absence of one or more signs. A video-recorded standard neurological examination will be included as part of the Core Module. An additional test of self-reported impairment, mainly due to movement disorders, will be administered as part of the Additional Optional Module (Figure 1).

A standard video recording of each participant accessing the study at one of the clinics will be performed and evaluated by a clinical neurologist. The assessment includes examination of strength, coordination, balance, ocular movements, cranial nerves, gait, and repeated movements. Theexamination includes also a test of knee functionality (84).

For peer review only - http://bmjopen.bmj.com/site/about/guidelines.xhtml

Information on potential movement disorders will be collected using the unified Parkinson's Disease Rating Scale (PDDRS) part II. This includes self-reported ratings on several motor domains, including speech, saliva and drooling, chewing and swallowing, eating, dressing, hygiene, handwriting, hobbies, turning in bed, tremor, standing up, walking and balance, and freezing (Additional optional module).

Intermediate neurological endpoints

In addition to neurological signs and symptoms, some intermediate outcomes have been recognised as part of the complex clinical picture of movement disorders, manifesting before the onset of the movement impairment itself. These will be investigated with three tests described below (Figure 1).

BRAIN tap test –This is a simple and rapid computerised keyboard test, based on the alternating finger tapping test, which has been developed to quantify upper limb motor function (85). The test generates several variables: 1) kinesia score: the number of keystrokes in 60 seconds; 2) akinesia time: cumulative time that keys are depressed; 3) dysmetria score: a weighted index calculated using the number of incorrectly hit keys corrected for speed; 4) in-coordination score: a measure of rhythmicity which corresponds to the variance of the time interval between keystrokes (85). The BRAIN test provides a simple, rapid, and objective assessment of upper limb motor function. It assesses speed, accuracy, and rhythmicity of upper limb movements regardless of their physiological basis. The results of the test correlate well with clinical rating scales in Parkinson's disease and cerebellar dysfunction. The BRAIN tap test is useful in clinical studies (85). The BRAIN tap test is available online at https://www.braintaptest.com/en_GB and VG has obtained access for generating tokens for administering the test to participants (A Noyce, personal communication)

University of Pennsylvania Smell Test – This is a measurement of the individual's ability to detect odours at a supra-threshold level (86). The test takes only a few minutes. The test consists of 4 different 10-page booklets, with a total of 40 questions. On each page, there is a different "scratch and sniff" strip, which is embedded with a microencapsulated odorant. There is also a four choice multiple choice question on each page. The scents are released using a pencil. After each scent is released, the patient smells the level, and detects the odour from the four choices. There is an answer column on the back of the test booklet, and the test is scored out of 40 items. The score is compared to scores in a normative database from 4000 normal individuals (87) (Additional optional module).

For peer review only - http://bmjopen.bmj.com/site/about/guidelines.xhtml

The REM-Behaviour Disorder Screening Questionnaire (RBDSQ) – This test is a validated 10-item patient self-rating questionnaire (maximum total score 13 points) covering the clinical features of REM Behavioral Disorders (RBD) (88). The RBDSQ was validated on 54 patients with polysomnographically confirmed RBD, 160 control subjects in whom RBD was excluded by history and polysomnography, and 133 unselected healthy subjects. Applying a positivity threshold of 5 points, the RBDSQ had a sensitivity of 0.96 and a specificity of 0.56 comparing patients with RBD with patients with other sleep disturbances. A specificity of 0.92 was calculated when comparing patients with RBD with patients without sleep disturbances (88) (Additional optional module).

Blood and saliva sample collection, and bio-banking

Participants seen in the research clinics will be asked to donate a blood sample for testing for tau protein, neurofilament (Nf) levels, full blood count analysis, and to obtain data for a GWAS. Samples of serum, plasma, and DNA will be collected and processed according to a pre-specified protocol and stored in a freezer at -80 °C at the Blizard Institute (Queen Mary, University of London). All participants will also asked to donate a blood sample through a dried blood spot test and a saliva sample (in order to obtain DNA where phlebotomy is not possible).

Samples will be tested to measure the expression of NfL (Neurofilament light) , a Nf-subunit showing consistent and reproducible measurements in plasma/serum using a newly developed sensitive methodology exploiting single molecule trapping and measurement by ELISA (SIMOA) (89-91). The use of both NfL and NfH (heavy) allows for the determination of different markers whose dynamic of release into bio fluids following injury vary according to their particular chemical structure. NfL has shown good levels of linearity on dilution experiments, suggesting that confounders like aggregation or immune response may not interfere with its measurements. NfH different phosphorylation states in blood may in turn provide more information on the systemic biological changes induced by trauma. The relative low abundance of these proteins in blood, in absence of CSF, which is normally more enriched in by-products of neuronal destruction, will not represent a major obstacle, as analytical sensitivity has evolved with the development of novel assays which cover the lower end of these protein dynamic range. SIMOA, the proposed methodology for the target biomarkers, is now emerging as the core technique for the measurement of structural proteins, down to the fempto-molar end of the spectrum. A novel SIMOA-based assay for the measurement of Tau in blood is now available. This development

For peer review only - http://bmjopen.bmj.com/site/about/guidelines.xhtml

offers a wide range of opportunities based on the possibility of overcoming the limitations imposed by CSF collection by lumbar puncture.

GWAS will be conducted at UCL in the lab led by Prof John Hardy.

Multimedia database set up and management

A multimedia database will be established as part of this project. The final dataset will include questionnaire and tests data (see Figure 1), in addition to the video-recorded neurological examination. This data will be also linked to the stored blood samples through a unique identifier.

The data will be stored in mirror, anonymised datasets at LSHTM and QMUL on secure servers, and access will be regulated by the study PIs who are ultimately responsible for maintaining confidentiality and data protection. Only the researcher(s) employed on this study will have access to subject identifiable information. Identifiable information (name, date of birth, and address) will be removed prior to analysis, and subjects will be identified by pseudoanonymised codes. Personal data and key codes for pseudoanonymisation will be stored in a database with restricted access. Before conducting the statistical analyses, checks will undertaken to ensure data quality.

Statistical analysis

Descriptive statistical methods (geometric means, geometric standard deviations, and 95% Confidence Intervals (95% C.I.) will be used to summarise the rugby history and lifestyle exposure data. Analyses linking a history of head trauma with neurological symptoms (obtained by questionnaire and neurobehavioural tests) will then be performed. Parameters of physical and cognitive capability, alongside clinical neurological intermediate endpoints, will initially be dichotomised (yes/no symptoms), and analysed with prevalence odds ratios using logistic regression (92). We will also analyse continuous outcome measures using multiple linear regression. In addition to analysing individual symptoms, we will assess associations with domains of symptoms (i.e. physical and cognitive capability). For this purpose we will use cut-points previously published in the literature (93), particularly related to the 1946 Birth Cohort whose testing protocol was used. For analyses involving repeated measurements (neurobehavioural testing) assessing acute and chronic effects, we will use multilevel models. Analyses will be adjusted for age, sex, and alcohol consumption.

For peer review only - http://bmjopen.bmj.com/site/about/guidelines.xhtml

Finally, we will compare neurological outcomes assessed by questionnaire with those assessed by computer-administered tests using Kappa-statistics (and in case of continuous outcomes linear regression analyses). We will also compare the strengths of the associations with exposure between both outcome measures.

Study size and power

We aim to invite 205 participants to participate, approximately half of whom have been exposed to concussion during their career (M Cross, personal communication). We conservatively estimate that about 150 former players will participate. Based on previous studies, the standard deviations of the psychometric tests are in the range of 8-15% of the absolute value; assuming a conservative figure of 15% overall, the study will have more than 95% power to detect a 10% difference, and 80% power to detect a 7% difference, in psychometric test scores, between exposed (to concussion) and non-exposed participants.

Limitations

The main limitation of this study is the cross-sectional design. Data on lifetime exposure (history of concussion) and health outcomes are collected at one time point, making data subject to recall bias. Participants with a poorer health, or sentiment of discontent with their health status, may overestimate concussive exposure, if they attribute their poor health to it.

However, this cross-sectional study design will provide meaningful data on the burden of ill health and neurological health among retired rugby players, in a relatively short two-year time frame. Depending on results (and availability of additional funding), this study could inform a, more detailed, and time intensive cohort study, aimed at assessing the association between concussion in rugby players and health outcomes prospectively.

A direct consequence of the cross-sectional design is an intrinsic risk of selection bias. Rugby players who have developed serious neurodegenerative conditions (or have died from them) in the meantime are less likely (or not able) to participate in the study thus limiting our sample to a subsample of healthier retired rugby players. On the other hand, it is possible that former players who experience some cognitive symptoms will be more keen to participate in the study, to seek reassurance and be examined in more detail.

For peer review only - http://bmjopen.bmj.com/site/about/guidelines.xhtml

Irrespective of these considerations, our investigation of the association between the exposure to sporting concussion and later-life general and neurological health outcomes remains valid, despite a smaller range of health outcomes diminishing the power for detecting an association as significant. Nonetheless, the power calculation for this study is calculated on the basis of continuous outcome measures (i.e. neurocognitive tests or grip strength measurement) making this study adequately powered to detect the potential associations, based on the expected number of recruited participants.

It is worth noting that results from this study will be not immediately apply to current rugby players, due to this cohort's former playing status, history of amateur playing exposure and alterations to rules and conditions, aimed at increasing plaer safety over recent years, which will have altered player's overall exposure to concussive events..

Ethics and dissemination

Ethical approval has been granted from the London School of Hygiene and Tropical Medicine (LSHTM) Ethics Committee. The study in general is focusing on subclinical problems (e.g. slightly lower than average scores in cognitive function tests) which would not usually require any medical attention. If any problems are identified, we will (with the permission of the study participant) refer participants to their primary care physician. The consent form includes the following statement:

- ☐ *I wish to inform my GP that I am taking part to this study*
- ☐ *I wish the study team to inform my GP of any unusual test result*

This study will provide unique insight into the physical and neurological health of retired rugby players who are now aged 50 years or more. This is valuable information *per se*, potentially comparable with other occupational cohorts or the general population (accepting the potential for bias discussed previously in comparing these samples). Moreover, within this study, data will be analysed as a function of the history of traumatic brain injury and concussion during their careers, and therefore assess associations between the history of reported concussive injuries and health-related outcomes.

Importantly, the neurological examination and collection of intermediate neurological outcomes, allows the detection of subtle preclinical changes that might be associated with future risk of developing a neurodegenerative condition. Studying to what extent these changes are also associated with history of

For peer review only - http://bmjopen.bmj.com/site/about/guidelines.xhtml

concussion will allow an estimation of the impact of concussion on the onset of neurodegenerative diseases in rugby players, thus providing the basis for future prospective studies.

A novel aspect of this project is the molecular component: clinical and neurocognitive findings will be particularly reinforced by the measurement of blood biomarkers. These will give a quantitative measurement of active neurodegeneration (NfL) and of possible CTE (tau), to be analysed conjunctly with the health-related outcomes and personal history of concussion.

Also, relying on a number characteristics of the rugby playing history collected (i.e. length of playing at elite level, position played, numbers of game played, age when started playing), it will be possible to identify potential categories of players at potentially increased risks of health-related outcomes.

This study may identify a small deviation from normality in any of the continuous measures collected via cognitive and other tests (i.e. grip strength, memory function, walking speed, etc). This will be mainly investigated in association with a history of frequent concussions, or other head traumas. The investigators will interpret the results based on the continuum of distribution of the test outcomes, which will not have specific clinical translation. However, it should be emphasised that although this study is unlikely to identify patterns suggesting a clinical disease in any of the participants, if these are identified, then the relevant participant(s) will be referred to appropriate medical services.

These data will be disseminated initially to players and sports governing bodies, in addition to through peer reviewed publication and conference presentation, in order to begin to establish an evidence basis for the association between exposure to sports-related concussion, and potential later life cognitive and neurological impairment.

For peer review only - http://bmjopen.bmj.com/site/about/guidelines.xhtml

References

1. Pearce N, Gallo V, McElvenny D. Head trauma in sport and neurodegenerative disease: an issue whose time has come? Neurobiol Aging. 2015;36(3):1383-9.
2. Lehman EJ. Epidemiology of neurodegeneration in American-style professional football players. Alzheimers Res Ther. 2013;5(4):34.
3. Chio A, Benzi G, Dossena M, Mutani R, Mora G. Severely increased risk of amyotrophic lateral sclerosis among Italian professional football players. Brain. 2005;128(Pt 3):472-6.
4. Belanger HG, Vanderploeg RD. The neuropsychological impact of sports-related concussion: a meta-analysis. J Int Neuropsychol Soc. 2005;11(4):345-57.
5. Kokmen E, Beard CM, O'Brien PC, Kurland LT. Epidemiology of dementia in Rochester, Minnesota. Mayo Clin Proc. 1996;71(3):275-82.
6. Nemetz PN, Leibson C, Naessens JM, Beard M, Kokmen E, Annegers JF, et al. Traumatic brain injury and time to onset of Alzheimer's disease: a population-based study. Am J Epidemiol. 1999;149(1):32-40.
7. Schofield PW, Tang M, Marder K, Bell K, Dooneief G, Chun M, et al. Alzheimer's disease after remote head injury: an incidence study. J Neurol Neurosurg Psychiatry. 1997;62(2):119-24.
8. Harris MA, Shen H, Marion SA, Tsui JK, Teschke K. Head injuries and Parkinson's disease in a case-control study. Occup Environ Med. 2013;70(12):839-44.
9. Rugbjerg K, Ritz B, Korbo L, Martinussen N, Olsen JH. Risk of Parkinson's disease after hospital contact for head injury: population based case-control study. BMJ. 2008;337:a2494.
10. Goldman SM, Tanner CM, Oakes D, Bhudhikanok GS, Gupta A, Langston JW. Head injury and Parkinson's disease risk in twins. Ann Neurol. 2006;60(1):65-72.
11. Seidler A, Hellenbrand W, Robra BP, Vieregge P, Nischan P, Joerg J, et al. Possible environmental, occupational, and other etiologic factors for Parkinson's disease: a case-control study in Germany. Neurology. 1996;46(5):1275-84.
12. Kuopio AM, Marttila RJ, Helenius H, Rinne UK. Environmental risk factors in Parkinson's disease. Mov Disord. 1999;14(6):928-39.
13. Bower JH, Maraganore DM, Peterson BJ, McDonnell SK, Ahlskog JE, Rocca WA. Head trauma preceding PD: a case-control study. Neurology. 2003;60(10):1610-5.
14. Lehman EJ, Hein MJ, Baron SL, Gersic CM. Neurodegenerative causes of death among retired National Football League players. Neurology. 2012;79(19):1970-4.
15. Savica R, Parisi JE, Wold LE, Josephs KA, Ahlskog JE. High school football and risk of neurodegeneration: a community-based study. Mayo Clin Proc. 2012;87(4):335-40.
16. Pupillo E, Messina P, Giussani G, Logroscino G, Zoccolella S, Chio A, et al. Physical activity and amyotrophic lateral sclerosis: a European population-based case-control study. Ann Neurol. 2014;75(5):708-16.
17. Belli S, Vanacore N. Proportionate mortality of Italian soccer players: is amyotrophic lateral sclerosis an occupational disease? Eur J Epidemiol. 2005;20(3):237-42.
18. Longstreth WT, McGuire V, Koepsell TD, Wang Y, van Belle G. Risk of amyotrophic lateral sclerosis and history of physical activity: a population-based case-control study. Arch Neurol. 1998;55(2):201-6.
19. Scarmeas N, Shih T, Stern Y, Ottman R, Rowland LP. Premorbid weight, body mass, and varsity athletics in ALS. Neurology. 2002;59(5):773-5.
20. DeKosky ST, Ikonomovic MD, Gandy S. Traumatic brain injury--football, warfare, and long-term effects. N Engl J Med. 2010;363(14):1293-6.

For peer review only - http://bmjopen.bmj.com/site/about/guidelines.xhtml

21. Decq P, Gault N, Blandeau M, Kerdraon T, Berkal M, ElHelou A, et al. Long-term consequences of recurrent sports concussion. Acta Neurochir (Wien). 2016;158(2):289-300.
22. Hume PA, Theadom A, Lewis GN, Quarrie KL, Brown SR, Hill R, et al. A Comparison of Cognitive Function in Former Rugby Union Players Compared with Former Non-Contact-Sport Players and the Impact of Concussion History. Sports Med. 2016.
23. McMillan TM, McSkimming P, Wainman-Lefley J, Maclean LM, Hay J, McConnachie A, et al. Long-term health outcomes after exposure to repeated concussion in elite level: rugby union players. J Neurol Neurosurg Psychiatry. 2016.
24. Dubois B, Hampel H, Feldman HH, Scheltens P, Aisen P, Andrieu S, et al. Preclinical Alzheimer's disease: Definition, natural history, and diagnostic criteria. Alzheimers Dement. 2016;12(3):292-323.
25. Teasdale G, Jennett B. Assessment of coma and impaired consciousness. A practical scale. Lancet. 1974;2(7872):81-4.
26. Levin HS, Diaz-Arrastia RR. Diagnosis, prognosis, and clinical management of mild traumatic brain injury. Lancet Neurol. 2015;14(5):506-17.
27. Jordan BD. The clinical spectrum of sport-related traumatic brain injury. Nat Rev Neurol. 2013;9(4):222-30.
28. McCrory P, Meeuwisse WH, Aubry M, Cantu B, Dvorak J, Echemendia RJ, et al. Consensus statement on concussion in sport: the 4th International Conference on Concussion in Sport held in Zurich, November 2012. J Am Coll Surg. 2013;216(5):e55-71.
29. Giza CC, Kutcher JS, Ashwal S, Barth J, Getchius TS, Gioia GA, et al. Summary of evidence-based guideline update: evaluation and management of concussion in sports: report of the Guideline Development Subcommittee of the American Academy of Neurology. Neurology. 2013;80(24):2250-7.
30. Sharp DJ, Jenkins PO. Concussion is confusing us all. Pract Neurol. 2015;15(3):172-86.
31. Malec JF, Brown AW, Leibson CL, Flaada JT, Mandrekar JN, Diehl NN, et al. The mayo classification system for traumatic brain injury severity. J Neurotrauma. 2007;24(9):1417-24.
32. Jordan BD. Chronic traumatic encephalopathy and other long-term sequelae. Continuum (Minneap Minn). 2014;20(6 Sports Neurology):1588-604.
33. NIH. Traumatic Brain Injury (TBI) Definition.
34. Johnson VE, Stewart W, Graham DI, Stewart JE, Praestgaard AH, Smith DH. A neprilysin polymorphism and amyloid-beta plaques after traumatic brain injury. J Neurotrauma. 2009;26(8):1197-202.
35. Kilbourne M, Kuehn R, Tosun C, Caridi J, Keledjian K, Bochicchio G, et al. Novel model of frontal impact closed head injury in the rat. J Neurotrauma. 2009;26(12):2233-43.
36. Kirchhoff C, Leidel BA, Kirchhoff S, Braunstein V, Bogner V, Kreimeier U, et al. Analysis of N-terminal pro-B-type natriuretic peptide and cardiac index in multiple injured patients: a prospective cohort study. Crit Care. 2008;12(5):R118.
37. Papa L, Ramia MM, Edwards D, Johnson BD, Slobounov SM. Systematic review of clinical studies examining biomarkers of brain injury in athletes after sports-related concussion. J Neurotrauma. 2015;32(10):661-73.
38. Zetterberg H, Blennow K. Fluid biomarkers for mild traumatic brain injury and related conditions. Nat Rev Neurol. 2016;12(10):563-74.
39. Shahim P, Tegner Y, Gustafsson B, Gren M, Arlig J, Olsson M, et al. Neurochemical Aftermath of Repetitive Mild Traumatic Brain Injury. JAMA Neurol. 2016;73(11):1308-15.
40. Shahim P, Zetterberg H, Blennow K. Neurofilament Protein and Antineurofilament Antibodies Following Traumatic Brain Injury-Reply. JAMA Neurol. 2017;74(3):363-4.
41. Shahim P, Zetterberg H, Tegner Y, Blennow K. Serum neurofilament light as a biomarker for mild traumatic brain injury in contact sports. Neurology. 2017;88(19):1788-94.

For peer review only - http://bmjopen.bmj.com/site/about/guidelines.xhtml

42. Julien JP, Mushynski WE. Neurofilaments in health and disease. Prog Nucleic Acid Res Mol Biol. 1998;61:1-23.
43. Perrone Capano C, Pernas-Alonso R, di Porzio U. Neurofilament homeostasis and motoneurone degeneration. Bioessays. 2001;23(1):24-33.
44. Lu CH, Allen K, Oei F, Leoni E, Kuhle J, Tree T, et al. Systemic inflammatory response and neuromuscular involvement in amyotrophic lateral sclerosis. Neurol Neuroimmunol Neuroinflamm. 2016;3(4):e244.
45. Posmantur R, Hayes RL, Dixon CE, Taft WC. Neurofilament 68 and neurofilament 200 protein levels decrease after traumatic brain injury. J Neurotrauma. 1994;11(5):533-45.
46. Oliver J, Jones M, Kirk M, Gable D, Repshas J, Johnson T, et al. Serum Neurofilament Light in American Football Athletes Over the Course of a Season. J Neurotrauma. 2015.
47. Gatson JW, Barillas J, Hynan LS, Diaz-Arrastia R, Wolf SE, Minei JP. Detection of neurofilament-H in serum as a diagnostic tool to predict injury severity in patients who have suffered mild traumatic brain injury. J Neurosurg. 2014;121(5):1232-8.
48. Shahim P, Tegner Y, Wilson DH, Randall J, Skillback T, Pazooki D, et al. Blood biomarkers for brain injury in concussed professional ice hockey players. JAMA Neurol. 2014;71(6):684-92.
49. Shahim P, Tegner Y, Marklund N, Hoglund K, Portelius E, Brody DL, et al. Astroglial activation and altered amyloid metabolism in human repetitive concussion. Neurology. 2017;88(15):1400-7.
50. Shahim P, Gren M, Liman V, Andreasson U, Norgren N, Tegner Y, et al. Serum neurofilament light protein predicts clinical outcome in traumatic brain injury. Sci Rep. 2016;6:36791.
51. Morris M, Maeda S, Vossel K, Mucke L. The many faces of tau. Neuron. 2011;70(3):410-26.
52. Panenka WJ, Gardner AJ, Dretsch MN, Crynen GC, Crawford FC, Iverson GL. Systematic Review of Genetic Risk Factors for Sustaining a Mild Traumatic Brain Injury. J Neurotrauma. 2017;34(13):2093-9.
53. Mc Fie S, Abrahams S, Patricios J, Suter J, Posthumus M, September AV. The association between harm avoidance personality traits and self-reported concussion history in South African rugby union players. J Sci Med Sport. 2017.
54. Naj AC, Schellenberg GD, Alzheimer's Disease Genetics C. Genomic variants, genes, and pathways of Alzheimer's disease: An overview. Am J Med Genet B Neuropsychiatr Genet. 2017;174(1):5-26.
55. Fuller GW, Kemp SP, Decq P. The International Rugby Board (IRB) Pitch Side Concussion Assessment trial: a pilot test accuracy study. British journal of sports medicine. 2015;49(8):529-35.
56. Hinton-Bayre AD, Geffen G, Friis P. Presentation and mechanisms of concussion in professional Rugby League Football. J Sci Med Sport. 2004;7(3):400-4.
57. Delaney JS, Al-Kashmiri A, Correa JA. Mechanisms of injury for concussions in university football, ice hockey, and soccer. Clin J Sport Med. 2014;24(3):233-7.
58. Davies MAM, A DJ, Delmestri A, S PTK, Stokes KA, Arden NK, et al. Health amongst former rugby union players: A cross-sectional study of morbidity and health-related quality of life. Sci Rep. 2017;7(1):11786.
59. Wadsworth M, Kuh D, Richards M, Hardy R. Cohort Profile: The 1946 National Birth Cohort (MRC National Survey of Health and Development). International journal of epidemiology. 2006;35(1):49-54.
60. Nofuji Y, Shinkai S, Taniguchi Y, Amano H, Nishi M, Murayama H, et al. Associations of Walking Speed, Grip Strength, and Standing Balance With Total and Cause-Specific Mortality in a General Population of Japanese Elders. J Am Med Dir Assoc. 2016;17(2):184 e1-7.
61. Cooper R, Kuh D, Hardy R. Objectively measured physical capability levels and mortality: systematic review and meta-analysis. BMJ. 2010;341:c4467.
62. Bohannon RW. Comfortable and maximum walking speed of adults aged 20-79 years: reference values and determinants. Age and ageing. 1997;26(1):15-9.

For peer review only - http://bmjopen.bmj.com/site/about/guidelines.xhtml

63. Schoon Y, Bongers K, Van Kempen J, Melis R, Olde Rikkert M. Gait speed as a test for monitoring frailty in community-dwelling older people has the highest diagnostic value compared to step length and chair rise time. Eur J Phys Rehabil Med. 2014;50(6):693-701.
64. Guralnik JM, Simonsick EM, Ferrucci L, Glynn RJ, Berkman LF, Blazer DG, et al. A short physical performance battery assessing lower extremity function: association with self-reported disability and prediction of mortality and nursing home admission. J Gerontol. 1994;49(2):M85-94.
65. Kuh D, Karunananthan S, Bergman H, Cooper R. A life-course approach to healthy ageing: maintaining physical capability. Proc Nutr Soc. 2014;73(2):237-48.
66. Roberts HC, Denison HJ, Martin HJ, Patel HP, Syddall H, Cooper C, et al. A review of the measurement of grip strength in clinical and epidemiological studies: towards a standardised approach. Age and ageing. 2011;40(4):423-9.
67. Jones CJ, Rikli RE, Beam WC. A 30-s chair-stand test as a measure of lower body strength in community-residing older adults. Res Q Exerc Sport. 1999;70(2):113-9.
68. Nilsdotter A, Bremander A. Measures of hip function and symptoms: Harris Hip Score (HHS), Hip Disability and Osteoarthritis Outcome Score (HOOS), Oxford Hip Score (OHS), Lequesne Index of Severity for Osteoarthritis of the Hip (LISOH), and American Academy of Orthopedic Surgeons (AAOS) Hip and Knee Questionnaire. Arthritis Care Res (Hoboken). 2011;63 Suppl 11:S200-7.
69. Collins NJ, Misra D, Felson DT, Crossley KM, Roos EM. Measures of knee function: International Knee Documentation Committee (IKDC) Subjective Knee Evaluation Form, Knee Injury and Osteoarthritis Outcome Score (KOOS), Knee Injury and Osteoarthritis Outcome Score Physical Function Short Form (KOOS-PS), Knee Outcome Survey Activities of Daily Living Scale (KOS-ADL), Lysholm Knee Scoring Scale, Oxford Knee Score (OKS), Western Ontario and McMaster Universities Osteoarthritis Index (WOMAC), Activity Rating Scale (ARS), and Tegner Activity Score (TAS). Arthritis Care Res (Hoboken). 2011;63 Suppl 11:S208-28.
70. Hamilton CB, Maly MR, Giffin JR, Clark JM, Speechley M, Petrella RJ, et al. Validation of the Questionnaire to Identify Knee Symptoms (QuIKS) using Rasch analysis. Health Qual Life Outcomes. 2015;13:157.
71. Folstein MF, Folstein SE, McHugh PR. "Mini-mental state". A practical method for grading the cognitive state of patients for the clinician. J Psychiatr Res. 1975;12(3):189-98.
72. Wechsler D. Wechsler Memory Scale - Revised Manual. Sant Antonio, TX: Psychological Corporation; 1987.
73. Papp KV, Amariglio RE, Dekhtyar M, Roy K, Wigman S, Bamfo R, et al. Development of a psychometrically equivalent short form of the Face-Name Associative Memory Exam for use along the early Alzheimer's disease trajectory. Clin Neuropsychol. 2014;28(5):771-85.
74. Rentz DM, Amariglio RE, Becker JA, Frey M, Olson LE, Frishe K, et al. Face-name associative memory performance is related to amyloid burden in normal elderly. Neuropsychologia. 2011;49(9):2776-83.
75. Aron AR, Monsell S, Sahakian BJ, Robbins TW. A componential analysis of task-switching deficits associated with lesions of left and right frontal cortex. Brain. 2004;127(Pt 7):1561-73.
76. Shallice T, Stuss DT, Picton TW, Alexander MP, Gillingham S. Multiple effects of prefrontal lesions on task-switching. Front Hum Neurosci. 2007;1:2.
77. Wechsler D. Wechsler Adult Intelligence Scale Sant Antonio, TX: Psychological Corporation; 1981.
78. Pertzov Y, Miller TD, Gorgoraptis N, Caine D, Schott JM, Butler C, et al. Binding deficits in memory following medial temporal lobe damage in patients with voltage-gated potassium channel complex antibody-associated limbic encephalitis. Brain. 2013;136(Pt 8):2474-85.
79. Parra MA, Abrahams S, Logie RH, Mendez LG, Lopera F, Della Sala S. Visual short-term memory binding deficits in familial Alzheimer's disease. Brain. 2010;133(9):2702-13.

For peer review only

For peer review only - http://bmjopen.bmj.com/site/about/guidelines.xhtml

80. Nelson HE, Willison J. The National Adult Reading Test (NART).
81. Say MJ, Jones R, Scahill RI, Dumas EM, Coleman A, Santos RC, et al. Visuomotor integration deficits precede clinical onset in Huntington's disease. Neuropsychologia. 2011;49(2):264-70.
82. Wechsler D. The Wechsler abbreviated scale of intelligence. San Antonio, TX: The Psychological Corporation; 1999.
83. Forster S, Lavie N. Entirely irrelevant distractors can capture and captivate attention. Psychon Bull Rev. 2011;18(6):1064-70.
84. Bremander AB, Dahl LL, Roos EM. Validity and reliability of functional performance tests in meniscectomized patients with or without knee osteoarthritis. Scand J Med Sci Sports. 2007;17(2):120-7.
85. Giovannoni G, van Schalkwyk J, Fritz VU, Lees AJ. Bradykinesia akinesia inco-ordination test (BRAIN TEST): an objective computerised assessment of upper limb motor function. J Neurol Neurosurg Psychiatry. 1999;67(5):624-9.
86. Doty RL, Shaman P, Dann M. Development of the University of Pennsylvania Smell Identification Test: a standardized microencapsulated test of olfactory function. Physiol Behav. 1984;32(3):489-502.
87. Doty RL, Frye RE, Agrawal U. Internal consistency reliability of the fractionated and whole University of Pennsylvania Smell Identification Test. Percept Psychophys. 1989;45(5):381-4.
88. Stiasny-Kolster K, Mayer G, Schafer S, Moller JC, Heinzel-Gutenbrunner M, Oertel WH. The REM sleep behavior disorder screening questionnaire--a new diagnostic instrument. Mov Disord. 2007;22(16):2386-93.
89. Gaiottino J, Norgren N, Dobson R, Topping J, Nissim A, Malaspina A, et al. Increased neurofilament light chain blood levels in neurodegenerative neurological diseases. PLoS One. 2013;8(9):e75091.
90. Kuhle J, Barro C, Andreasson U, Derfuss T, Lindberg R, Sandelius A, et al. Comparison of three analytical platforms for quantification of the neurofilament light chain in blood samples: ELISA, electrochemiluminescence immunoassay and Simoa. Clin Chem Lab Med. 2016;54(10):1655-61.
91. Rohrer JD, Woollacott IO, Dick KM, Brotherhood E, Gordon E, Fellows A, et al. Serum neurofilament light chain protein is a measure of disease intensity in frontotemporal dementia. Neurology. 2016;87(13):1329-36.
92. Pearce N. Effect measures in prevalence studies. Environ Health Perspect. 2004;112(10):1047-50.
93. Kaukiainen A, Hyvarinen HK, Akila R, Sainio M. Symptoms of chronic solvent encephalopathy: Euroquest questionnaire study. Neurotoxicology. 2009;30(6):1187-94.

For peer review only

For peer review only - http://bmjopen.bmj.com/site/about/guidelines.xhtml

Table 1: Glossary of definitions, adapted from (32)

**Chronic traumatic brain injury (TBI)**	A spectrum of disorders associated with long-term consequences of single or repetitive TBI
**Chronic traumatic encephalopathy (CTE)^**	Prototypic chronic TBI, long-term neurologic consequences of repetitive mild TBI
**Dementia pugilistica**	A subtype of CTE that is typically reserved for cases of severe end-stage dementia secondary to a long boxing career
**Chronic post-concussion syndrome^**	A condition in those athletes in whom post-concussion symptoms do not appear to resolve
**Chronic neurocognitive impairment^**	A rather diverse classification of chronic neurocognitive signs and symptoms secondary to head impact exposures and recurrent concussions that is theoretically distinctive from CTE
**Post-traumatic dementia***	Cases in which the athlete meets clinical criteria for dementia secondary to a single moderate to severe TBI
**Post-traumatic cognitive impairment***	Individuals who sustain long-term neurocognitive deficits from a single moderate to severe brain injury and do not meet clinical criteria for dementia, but instead mild cognitive impairment
**Post-traumatic parkinsonism**	A parkinsonian-like syndrome secondary to a single moderate to severe or repetitive TBI, occurring solely or as a component of CTE

^ the most clinically pertinent examples of sports-related chronic TBI ; *consequence of a single brain injury

For peer review only

Figure 1: Framework for data collection in the BRAIN study by domain

Footnote of Figure1:

*not to be collected if the participant is seen at home; ^subject to suitable chair availability for the participants seen at home; ~including knee bending test; #subject to space availability of the participant is seen at home; ≮to be administered as last item in all cases (<u>after all 'Core' and Additional Optional module tests</u>)

For peer review only

Authors' contributions: state how each author was involved in writing the protocol.

Author's contributions

Valentina Gallo – co-Principal Investigator. Drafted the protocol, collected comments, and act as corresponding author

Damien McElvenny – co-Principal Investigator. Contributed designing the protocol, reviewed intellectual content of this paper

Huw Morris – Co-investigator. Advised on cognitive testing, biomarkers, and clinical definitions, reviewed this paper for intellectual content

Sebastian Crutch – Co-investigator. Advised on cognitive testing, and clinical definitions, reviewed this paper for intellectual content

Simon Kemp – Co-investigator. Advised on recruitment strategy, patient and public involvement (PPI) and reviewed this paper for intellectual content

Catherine Hobbs – Research assistant of the BRAIN Study. Recruits and collects informations on the study participants

Donna Davoren - Project administrator of the BRAIN Study. Coordinates the logistics of the study

Nigel K. Arden – Co-investigator. Advised on recruitment strategy, patient and public involvement (PPI) and reviewed this paper for intellectual content

Andrea Malaspina – Co-investigator. Advised on biomarkers

Matt Cross - Co-investigator. Advised on recruitment strategy, patient and public involvement (PPI) and reviewed this paper for intellectual content

Madeleine A.M.Davies - Co-investigator. Advised on recruitment strategy, patient and public involvement (PPI), and reviewed this paper for intellectual content

Henrik Zetterberg – Co-investigator. Advised on biomarkers and reviewed this paper for intellectual content

Nick Fox - Co-investigator. Reviewed this paper for intellectual content

Neil Pearce – co-Principal Investigator. Contributed designing the protocol, reviewed intellectual content of this paper

Funding statement:

This study is funded by a grant from the Drake Foundation (www.drakefoundation.org/) to the London School of Hygiene and Tropical Medicine, with subcontracts to Queen Mary University of London (QMUL) and the Institute of Medicine (IoM).

For peer review only

Competing interest statement

None

Data sharing statement

The full protocol including all tests is available upon request to researchers intending conducting a similar study

For peer review only

For peer review only - http://bmjopen.bmj.com/site/about/guidelines.xhtml

	Questionnaire	Physical ability	Cognitive ability	Neurological examination	Intermediate neurological outcomes	Blood sample
**'Core' Module**	•Lifestyle and confounders questionnaire<br>•Concussion history questionnaire≠	•Height<br>•Weight<br>•Grip strength<br>•Chair rise^<br>•Walking speed#<br>•Photograph of hands	•MMSE<br>•WMS-R logical memory<br>•FNAME-12A<br>•Task-set shifting/Response inhibition<br>•WAIS-R digit symbol<br>•Visual short-term memory binding<br>•NART	•Video-recording~	•BRAIN test	•DNA*<br>•Plasma*
**Additional Optional Module**	•Pain mannequin (hand)	• HOOS<br>•KOOS<br>•QuIKS	•Visuomotor integration<br>•Matrix reasoning (WASI)<br>•Irrelevant Distractor Paradigm	•UPDRS-II	•Smell test<br>•RBD questionnaire	

Framework for data collection in the BRAIN study by domain

1122x640mm (120 x 120 DPI)

review only

STROBE Statement—Checklist of items that should be included in reports of ***cross-sectional studies***

	Item No	Recommendation	
**Title and abstract**	1	(*a*) Indicate the study's design with a commonly used term in the title or the abstract	OK
		(*b*) Provide in the abstract an informative and balanced summary of what was done and what was found	OK
**Introduction**			
Background/rationale	2	Explain the scientific background and rationale for the investigation being reported	OK, pp. 3-9
Objectives	3	State specific objectives, including any prespecified hypotheses	OK, pp. 9-10
**Methods**			
Study design	4	Present key elements of study design early in the paper	OK, pp. 10-11
Setting	5	Describe the setting, locations, and relevant dates, including periods of recruitment, exposure, follow-up, and data collection	OK, p. 10 as part of study design
Participants	6	(*a*) Give the eligibility criteria, and the sources and methods of selection of participants	OK, p.9
Variables	7	Clearly define all outcomes, exposures, predictors, potential confounders, and effect modifiers. Give diagnostic criteria, if applicable	OK, pp.10-18
Data sources/ measurement	8*	For each variable of interest, give sources of data and details of methods of assessment (measurement). Describe comparability of assessment methods if there is more than one group	OK, pp.10-18
Bias	9	Describe any efforts to address potential sources of bias	
Study size	10	Explain how the study size was arrived at	OK, pp.19-20
Quantitative variables	11	Explain how quantitative variables were handled in the analyses. If applicable, describe which groupings were chosen and why	OK, pp18-19
Statistical methods	12	(*a*) Describe all statistical methods, including those used to control for confounding	pp.18-19
		(*b*) Describe any methods used to examine subgroups and interactions	pp.18-19
		(*c*) Explain how missing data were addressed	TBD
		(*d*) If applicable, describe analytical methods taking account of sampling strategy	NA
		(*e*) Describe any sensitivity analyses	TBD
**Results**			
Participants	13*	(a) Report numbers of individuals at each stage of study—eg numbers potentially eligible, examined for eligibility, confirmed eligible, included in the study, completing follow-up, and analysed	NA
		(b) Give reasons for non-participation at each stage	NA
		(c) Consider use of a flow diagram	NA
Descriptive data	14*	(a) Give characteristics of study participants (eg demographic, clinical, social) and information on exposures and potential confounders	NA
		(b) Indicate number of participants with missing data for each variable of interest	NA
Outcome data	15*	Report numbers of outcome events or summary measures	NA

For peer review only

Main results	16	(*a*) Give unadjusted estimates and, if applicable, confounder-adjusted estimates and their precision (eg, 95% confidence interval). Make clear which confounders were adjusted for and why they were included	NA
		(*b*) Report category boundaries when continuous variables were categorized	NA
		(*c*) If relevant, consider translating estimates of relative risk into absolute risk for a meaningful time period	NA
Other analyses	17	Report other analyses done—eg analyses of subgroups and interactions, and sensitivity analyses	NA
**Discussion**			
Key results	18	Summarise key results with reference to study objectives	NA
Limitations	19	Discuss limitations of the study, taking into account sources of potential bias or imprecision. Discuss both direction and magnitude of any potential bias	pp.19-20
Interpretation	20	Give a cautious overall interpretation of results considering objectives, limitations, multiplicity of analyses, results from similar studies, and other relevant evidence	NA
Generalisability	21	Discuss the generalisability (external validity) of the study results	P. 21
**Other information**			
Funding	22	Give the source of funding and the role of the funders for the present study and, if applicable, for the original study on which the present article is based	P.29

*Give information separately for exposed and unexposed groups.

Note: An Explanation and Elaboration article discusses each checklist item and gives methodological background and published examples of transparent reporting. The STROBE checklist is best used in conjunction with this article (freely available on the Web sites of PLoS Medicine at http://www.plosmedicine.org/, Annals of Internal Medicine at http://www.annals.org/, and Epidemiology at http://www.epidem.com/). Information on the STROBE Initiative is available at www.strobe-statement.org.

For peer review only
